# Prediction of Solute Descriptors for Linear Solvation Energy Relationships Using K‑Nearest Neighbors, Group Contributions, and Graph-Convolutional Neural Networks

**DOI:** 10.1021/acsenvironau.6c00063

**Published:** 2026-06-10

**Authors:** Nadin Ulrich, Valentin Istomin, Anton Kudria, Alexander Böhme, Karsten Voigt

**Affiliations:** † Department of Exposure Science, 28342 Helmholtz Centre for Environmental Research − UFZ, Permoserstrasse 15, Leipzig D-04318, Germany; ‡ PAULY, Essener Straße 100, Leipzig D-04357, Germany

**Keywords:** Linear Solvation Energy Relationship (LSER) models, Graph-Convolutional Neural Network, Group Contribution Approach, k-Nearest Neighbors Approach, Environmental Fate Modeling

## Abstract

Linear solvation energy relationship (LSER) models are nowadays often used to predict physicochemical properties of chemicals, such as partition coefficients, retention factors in chromatography, and solubilities. Due to their mechanistic foundation and transferability across phases, LSER models are particularly valuable for predicting partition coefficients in data-poor systems where experimental data sets are not available. However, their broader applicability is currently constrained by the limited availability of experimentally determined solute descriptors. We developed three different models, based on three different approaches, to predict the solute descriptors *S*, *E*, *A*, *B*, and *L* for LSER applications: (1) a group contribution model that includes an initial screening algorithm to identify functional groups as structural patterns, (2) a k-nearest neighbors model, and (3) a graph-convolutional neural network. All models were developed on the same curated data set for each solute descriptor. An independent test set was used to evaluate the overall model performance, with *rmse* values ranging from 0.08–0.13 for *A*, 0.10–0.15 for *B*, 0.17–0.23 for *S*, 0.09–0.19 for *E*, and 0.25–0.45 for *L* across the three approaches. By enabling the prediction of these descriptors directly from molecular structure, our modeling framework addresses a key bottleneck that has so far limited the scalability and broader application of the LSER models. In addition, we investigated whether a consensus approach would enhance overall prediction quality. Additionally, the predicted solute descriptors were directly used to derive environmentally and analytically relevant partition coefficients, demonstrating that reliable LSER-based property predictions are feasible even for chemicals lacking experimental descriptor data or large property-specific training sets. By enabling descriptor generation at scale, this work improves the practical applicability of LSER modeling and strengthens its role as a transferable and data-efficient tool for environmental fate and risk assessment.

## Graphical abstract

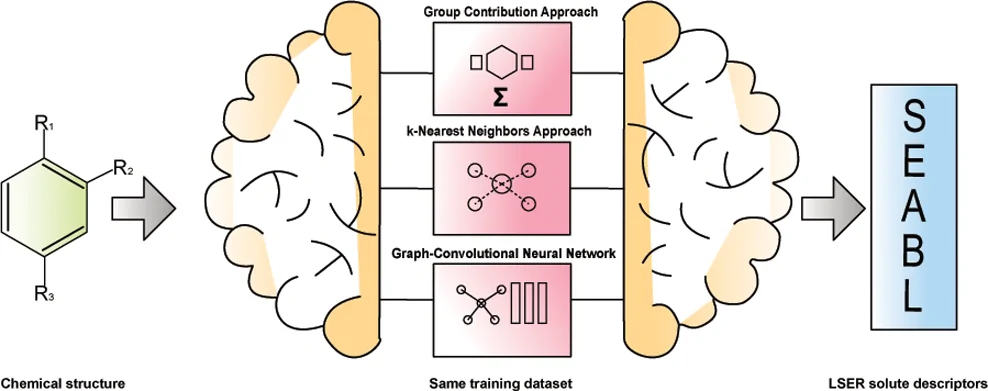

## 1 Introduction

Predicting physicochemical properties has become increasingly important across fields such as environmental science,^1,2^ pharmaceutical science,^3,4^ and materials design.^5^ Artificial neural networks (ANNs) and deep learning are powerful tools for developing quantitative structure–property relationship (QSPR) models,^6,7^ allowing to predict water solubility,^8,9^ the octanol–water partition coefficient,^10,11^ melting point,^12^ boiling point,^13,14^ and viscosity.^15^ For these parameters, a large amount of experimental data is available, which is needed for training ANNs.^16^ Compared to other approaches, such as group-contribution (GCA) or k-nearest neighbor (kNN) models, the performance of ANNs is often improved.^10,17^ However, experimental data sets for physicochemical properties are typically scarce, limiting the overall applicability of these models to only a few parameters.

Here, linear solvation energy relationships (LSERs) can be used to develop models for physicochemical properties using small sets of experimental data.^18–20^ These models are mainly used to describe partition coefficients for two-phase systems. More than 140 LSER equations for solvent–water systems and 130 LSER equations for solvent-air systems are available.^21^ Further, these models were applied to describe solubilities of organic compounds in certain solvents,^19^ to characterize technical sorbents like polydimethylsiloxane PDMS (in the form of PDMS-water and PDMS-air partition coefficients)^22^ or polymers like polyethylene (PE, in the form of PE-water partition coefficients),^23^ biological systems like storage lipids^24,25^ or vitellogenin,^26^ and even retention times in gas^27–29^ and liquid chromatography applications.^30,31^

The models are usually developed based on a set of 35–200 experimentally determined partition coefficients or solubilities for chemicals, for which the corresponding solute descriptors are available.^26,31–34^ The LSER equations to predict the solute property (SP) are composed of five different terms and a system constant *c* (eqs 1 and 2), describing potential interactions between a chemical (represented by the solute descriptor as an upper-case letter) and the corresponding two-phase system (in case of the partition coefficients, represented by the corresponding phase parameter as a lower-case letter).^35^

1 SP=eE+sS+vV+aA+bB+c

2 SP=eE+sS+lL+aA+bB+c

The *eE* term describes van-der-Waals interactions between the chemical and the surrounding environment, whereas the solute descriptor *E* is the excess molar refraction of the solute (covering weak dispersion interactions, e.g.). The *sS* term describes polarizability and dipolarity; the *aA* and *bB* terms depict hydrogen-bond interactions with the hydrogen-bond donor strength *A* and the hydrogen-bond acceptor strength *B* for the solute; and, for the corresponding phase parameters *a* and *b*, it is vice versa. The *vV* term includes the McGowan characteristic volume as a solute descriptor^36^ and cavity formation as a phase parameter. Note that eq 1 is commonly used to describe solvent–water systems. In the case of solvent-air systems (eq 2), the *vV* term is replaced by the *lL*, where the solute descriptor *L* represents the hexadecane-air partition coefficient. Note that the solute descriptors are dimensionless.

To date, about 8,000 experimentally determined solute descriptors are available.^21,29,37^ However, often not a full set of solute descriptors is available, limiting the number of chemicals covered to ∼6,300. Different models have been developed in recent years to predict these descriptors. Brown et al. redeveloped a GCA originally implemented in LSERD,^21,38,39^ a database that provides solute descriptors and phase parameters. Ulrich and Ebert established an ANN^40^ based on the Absolv data set implemented in LSERD. Furthermore, Zhang and Zhang developed a deep neural network DNN using the PaDEL framework.^41^ At the same time, Lang and Lee trained a large language model (LLM) to predict solute descriptors and refined phase parameters.^42^

This study aimed to develop three models to predict solute descriptors: a GCA based on a functional-group detection method established by Ertl,^43,44^ a kNN approach based on the method of Kühne et al.,^45^ and a graph convolutional neural network (GNN). These three models were compared, enabling the identification of potential strengths and weaknesses in predicting solute descriptors. For this, we selected an independent test set of chemicals with experimental solute descriptors to evaluate predictive performance. Moreover, we characterized the complete set of experimental solute descriptors with respect to the chemical space that they cover. Finally, we applied a consensus approach based on the three models developed and evaluated whether it allows for more accurate predictions of solute descriptors.

## 2 Methods and Materials

### 2.1 Solute Descriptor Data Set

The Absolv database of Michael Abraham, which is implemented as Abraham Absolv in the online database LSERD,^21^ was used to develop the three models. A further curation of the database was done in a previous publication.^40^ The up-to-date data set (May 2026) is provided in the corresponding GitHub repository https://github.com/nadinulrich/solute_descriptor_prediction. In total, 6,364 chemicals were included in the data, where the number of chemicals for the corresponding descriptors differs, ranging from 5,572 (*L* descriptor) to 6,364 (*S* descriptor). First, 10% of the corresponding descriptors were randomly split for each descriptor data set as an independent test set to allow for a direct comparison of the different models developed. The remaining subsets were divided in 70% for training (of the total set) and 20% for validation. The split into training and validation sets was performed 5-fold for each descriptor to enable cross-validation. Note that for kNN model development 90% of the data were used as this is a nonparametric, instance-based method where the predictions are directly derived from local neighbors in the training set.

### 2.2 Group Contribution Approach GCA

The GCA approach was implemented in Python (3.10.15), using NumPy (1.24.4),^46^ RDKit (2024.9.5),^47^ and NetworkX (3.4.2)^48^ for the algorithmic framework. The training and parameter optimization were performed by the SciPy (1.14.0)^49^ and lmfit (1.3.2)^50^ libraries. The approach has been developed by adopting the functional group definition from Ertl^44^ via its implementation by Colmenarejo.^51^ Here, the functional groups are considered as optimizable parameters whose linear combination allows for a calculation of a system’s properties. Two additional parameters are employed to represent the carbon backbone and aromaticity. Also, since Ertl’s definition does not limit the size of the functional groups and therefore theoretically allows for an infinite number of them, the large and rarely occurring groups are decomposed into smaller more common groups.

### 2.3 K-Nearest Neighbor Approach kNN

The kNN model was developed in Python (3.13.1) using NumPy (2.2.2),^46^ pandas (2.2.3),^52,53^ RDKit (2024.09.4),^47^ and python-igraph (0.11.8).^54^ Additionally, scikit-learn (1.6.1)^55^ and Optuna (4.4.0)^56^ were used for parameter optimization, and Matplotlib (3.10.0)^57^ for evaluation of test results. The model is a modified implementation of the read-across approach from Kühne et al.^45^ It uses Jaccard distance in the space of ACF (atom-centered fragment) counts to identify the nearest neighbors. Separate kNN predictions are made based on different ACF orders, which are then combined as a weighted sum.

In the current implementation, some of the parameters fixed in Kühne et al.^45^ were treated as hyperparameters, and their values were optimized via parameter sweeping. Additional hyperparameters compared to the model of Kühne et al.^45^ include, for example, the number of ACF orders considered, as well as some variable atom and bond properties for different neighbor orders and ACF orders. All hyperparameters were optimized separately for each solute descriptor.

### 2.4 Graph-Convolutional Neural Networks GNN

The GNN models were developed in Python 3.11.8 using TensorFlow 2.15.0 ^58^ and Keras 2.15.0.^59^ Additionally, the DeepChem version 2.7.2 library was applied.^60,61^ In brief, the chemical’s SMILES was used to generate molecular graphs, where atoms were embedded as nodes and bonds as edges in the input layer. For each descriptor, the optimal setup was determined by hyperparameter optimization, yielding the same setup and parameters across all GNNs trained. Two hidden layers, with 32 and 64 neurons, respectively, were included in the GNN. A learning rate of 0.001, the ReLU activation function, and an L1 Loss function were used. A dropout rate of 0.1 was applied, and we selected a batch size of 50 chemicals. The number of epochs varied for the different descriptors: 160 epochs for the *S* descriptor, 100 epochs for the *E* descriptor, 50 epochs for the *A* descriptor, 140 epochs for the *B* descriptor, and 85 epochs for the *L* descriptor.

## 3 Results and Discussion

### 3.1 Coverage of the Chemical Space by Chemicals with Experimental Solute Descriptors

As experimental solute descriptors are available for about 6,300 chemicals, we investigated which chemical space they cover. We therefore generated a UMAP plot showing the first two components of a nonlinear manifold projection (Figure 1, SI1–1). We used the PubChemLite database^62,63^ to represent the chemical space. However, we acknowledge that this database does not contain all commercially available chemicals, nor can it depict all potential energetically stable structures. There are other data sets available, such as the ZINC database^64^ or the GDB data sets;^65^ however, the latter has limitations regarding the elements present in the corresponding structures and their sizes. ZINC has the restriction that mainly drug-like structures and ligands are included. To cover a more heterogeneous chemical space, we thus used PubChemLite.^63^

**Figure 1:**
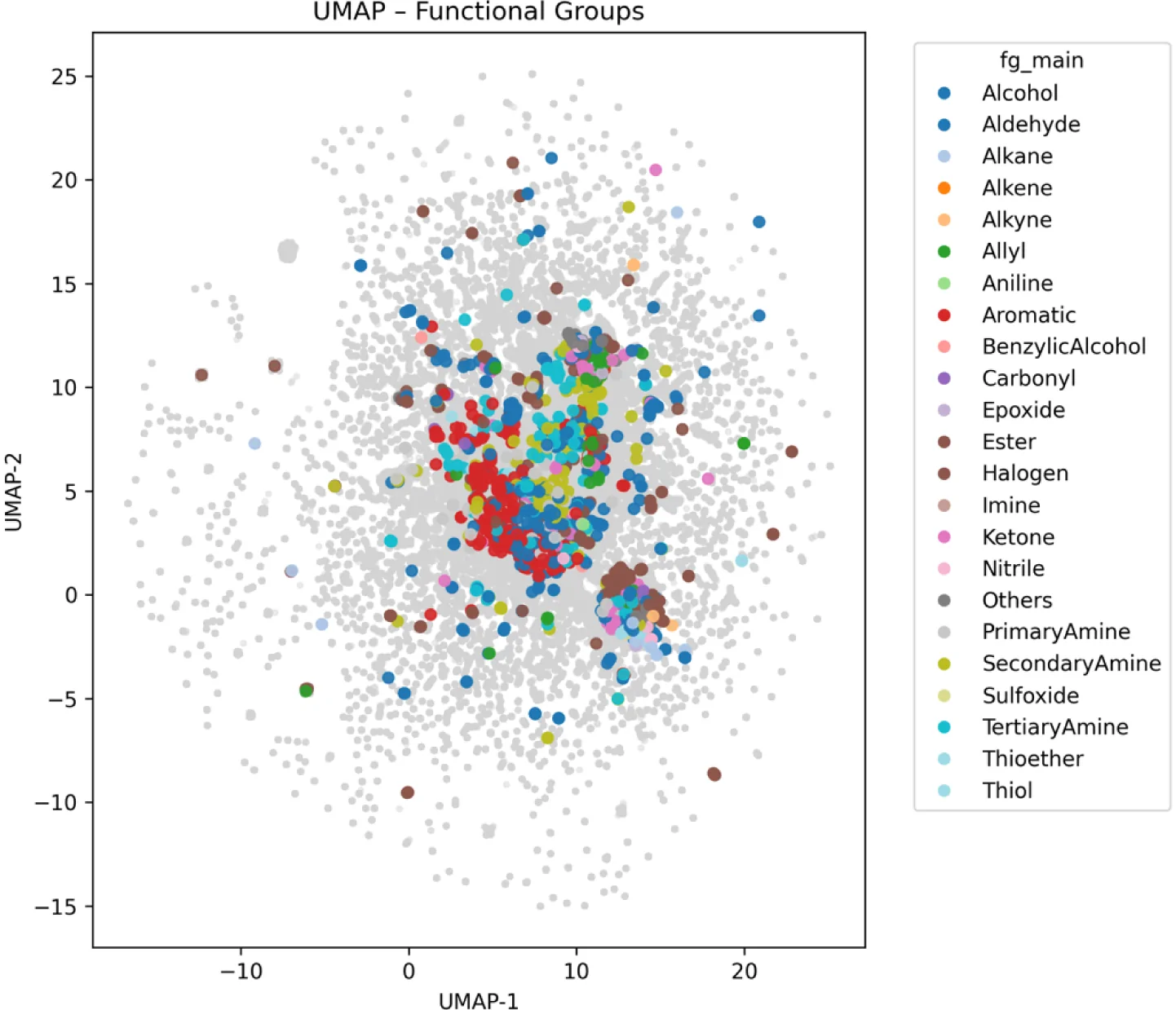
In the UMAP plot, the PubChemLite database represents the chemical space in light gray; the solute descriptor *S* is used to demonstrate the coverage of the experimental solute descriptor values relative to the overall chemical space. Note that this is a nonlinear manifold projection; distances reflect similarities in the corresponding chemical structure.

In Figure 1, the distances in the embedded space reflect molecular similarity relationships. Note that these distances should be interpreted with respect to physicochemical properties. We depicted the chemicals in PubChemLite in light gray and used colored marks for those with available experimental descriptors. We marked present functional groups with different colors to indicate that chemicals belong to specific clusters in the chemical space plot. As shown in Figure 1, most chemicals with experimental descriptors are clustered in the center of the chemical space plot. As experimental descriptors for relatively simple chemicals with one or two functional groups are most commonly available, this is reasonable. Also, more complex chemicals with multiple functional groups and heterogeneous composition can be found at the edges of the chemical space plot, as there are not many similar structures in PubChemLite. Figure 1, therefore, points out some drawbacks of the empirical-based LSER approach. As the experimental descriptors mainly cover simple chemical structures, the approach is limited for more complex structures. Especially for chemicals that involve intramolecular interactions (e.g., hydrogen bonds), the LSER approach tends to be more prone to error. These interactions depend on the different conformers of a molecule in the liquid phase, which are not accounted for in the approach.

Furthermore, the assumption of thermodynamic linearity, in which complex surface interactions are reduced to a few parameters, might be another shortcoming. Especially the *A* and *B* descriptors, which cover the overall H-bond capacity, may not fully capture site-specific effects or steric hindrance. Similarly, the *S* descriptor aggregates various dipolar and electrostatic interactions into a single term.

Additionally, ionic chemicals are not covered by the approach at all. Because Coulomb interactions strongly affect physicochemical properties such as solubilities and partition coefficients, the LSER approach is not suitable for describing such chemicals.

However, the UMAP representation can be interpreted with respect to the applicability-domain and confidence-oriented analysis of the chemical space. Compounds located in sparsely populated regions may correspond to more extrapolative prediction scenarios with potentially increased uncertainty, which is particularly relevant for structurally complex compounds with limited experimental reference data.

### 3.2 Solute Descriptor Data Set and Data Density

The Absolv data set contains a different number of chemicals for each descriptor. The smallest number of data points is available for the *L* descriptor (5,572 chemicals), followed by the *B* descriptor (6,111 chemicals). For the *E* descriptor, 6,347 data points are available; for the *A* descriptor, 6,358 chemicals; and for the *S* descriptor, 6,364 chemicals. To depict the distribution of the corresponding values for the different solute descriptors, we used density plots (Figure 2).

**Figure 2:**
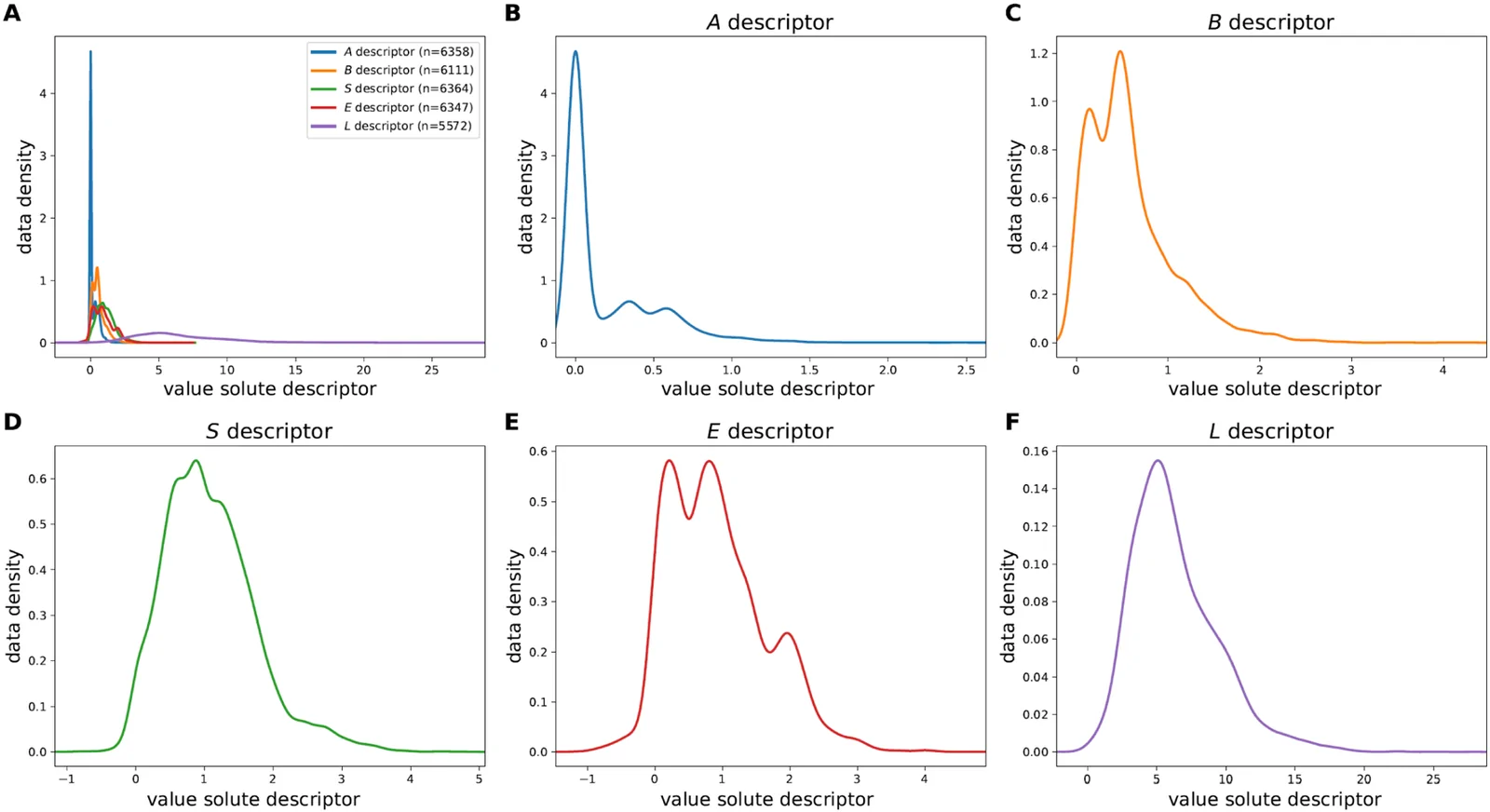
Density plots of the different solute descriptors. (A) All solute descriptors are included to show the variance of the data densities, data density of the values of the (B) *A* descriptor, (C) *B* descriptor, (D) *S* descriptor, (E) *E* descriptor, and (F) *L* descriptor.

The value ranges differ for the solute descriptors (see Figure 2A): *A* ranges from 0 to 2.50, *B* from 0 to 4.26, and *E* from −1.18 to 4.62. The *S* descriptor ranges from −0.89 to 4.80, and *L* shows the largest range, from −0.82 to 27.44. It should be noted that negative solute descriptors are often assigned to perfluorinated chemicals. Furthermore, there is a significant value imbalance in the *A* descriptor data set, with 60% of data points having a value of zero. This can be explained by the fact that the presence of functional groups that act as hydrogen bond donors is the only metric influencing the *A* descriptor, and the absence of them directly results in the *A* descriptor dropping to zero.

Based on these data sets, we developed three independent models to predict the solute descriptors of chemicals. Each data set was split into a training set (70%), validation set (20%), and test set (10%). The test set is used as an independent data set to evaluate the overall performance of the developed models and covers the same chemicals across all models. This test set was also used previously by Ulrich and Ebert,^40^ allowing for a direct comparison of the models’ performance. The splits into training and validation sets were automatically generated in the corresponding modeling workflows and therefore independently for each model developed. For the GNN models and GCA models, five random splits were applied. For the kNN models, this split was usually not used for model development, but 90% of the corresponding data set was used as the training set (to include the maximum number of chemicals). Only the test set is used to evaluate the model’s performance.

### 3.3 Model Development of the Group Contribution Approach

The first approach applied was a GCA model, in which chemical functional groups were identified using the Ertl method.^43,44^ These functional groups were used as fragments during model development. They were complemented with two additional parameters, selected to best represent the aliphatic or aromatic carbon backbone of the corresponding molecules. Additionally, large and uncommon functional groups are decomposed into smaller, more common groups. Such a decomposition is possible under the assumption that any large functional group can be viewed as a linear combination of the contributions of smaller groups it incorporates.^44^ As this is only a rough approximation, all predictions where such a decomposition occurred are marked with a “moderate” confidence level. In contrast, predictions where all functional groups have an optimized parameter are considered to have “high” confidence. Finally, if functional groups are encountered that neither have trained parameters available nor can be fully decomposed, the prediction for this molecule is marked with a “low” confidence level.

Five independent models were developed based on five different splits of the training and validation sets, enabling 5-fold cross-validation. In the end, all training results for all five models were collected, and, carefully adhering to the parameter limits determined during training, the uncertain parameters were optimized one final time using the test set as guidance. In the discussion that follows, this final model will be referred to as the “consensus model”. Details on the results can be found in Figure 3, Table 1, S1–2, and in SI2–1.

**Table 1:** Comparison of the *RMSE* Values of the Different Approaches Applied to Predict the Solute Descriptors for the Independent Test Set

Solute descriptor	Range of experimental value	GCA consensus	kNN	GNN consensus
*A*	0.00–2.50	0.119	0.126	0.075
*B*	0.00–4.26	0.142	0.151	0.095
*S*	–0.89–4.80	0.234	0.216	0.169
*E*	–1.18–4.62	0.188	0.131	0.086
*L*	–0.82–27.44	0.420	0.454	0.246

**Figure 3:**
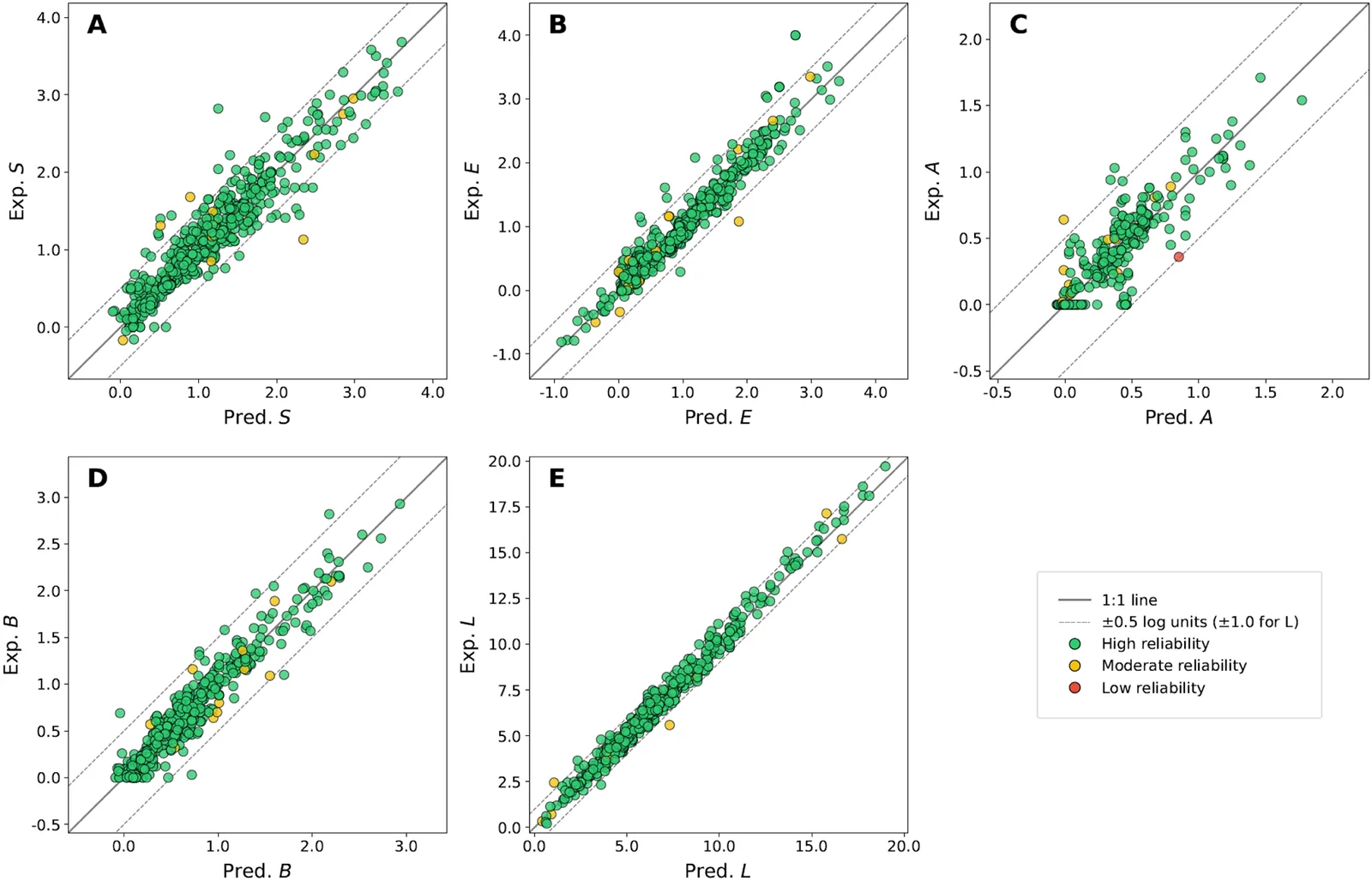
Experimental solute descriptors are plotted against the predicted ones based on the GCA approach for the (A) *S* descriptor, (B) *E* descriptor, (C) *A* descriptor, (D) *B* descriptor, and (E) *L* descriptor. Note that the quality and reliability of the prediction are depicted by the color: green: high reliability, yellow: moderate reliability, and red: low reliability.

The five models developed to predict the *S* descriptor show overall *r* ^2^ values of 0.834–0.863 and *rmse* values of 0.25–0.28 across the five random splits. The *r* ^2^ values for the test set across the five models range from 0.871 to 0.877, and the *rmse* values range from 0.24 to 0.25. The results from the validation and test sets are consistent across all the models developed. For the *E* descriptor, the corresponding *r* ^2^ values in the validation sets range from 0.924 to 0.934; the test set *r* ^2^ values range from 0.934 to 0.937. The *rmse* values of the five validation sets and the test set range from 0.19 to 0.20. The models for predicting *A* showed *r* ^2^ values of 0.819–0.860 for the validation sets and 0.826–0.844 for the test sets, with *rmse* values of 0.11–0.13 for the validation sets and 0.12–0.13 for the test sets. The validation set *rmse* values for the *B* descriptor range from 0.14 to 0.15, with *r* ^2^ values from 0.893 to 0.902. The corresponding *rmse* values for the test set are 0.15 for each model, with *r* ^2^ values from 0.917 to 0.922. For the *L* descriptor, the *rmse* values range from 0.45 to 0.49 on the validation set, with *r* ^2^ values of 0.976 to 0.979; the test set *rmse* values are 0.44 to 0.45, and the corresponding *r* ^2^ values are 0.982 to 0.983.

In general, the results for the five validation set predictions and the independent test set predictions are comparable, demonstrating the robustness of the models developed. Further, the results for the validation and test sets are consistent. The results of the consensus model (in this case, the consensus GCA and consensus GNN models) are provided in Table 1. In total, the *rmse* values could be improved for the independent test sets of the five solute descriptors, with *rmse* values of 0.12, 0.14, 0.23, 0.19, and 0.42 for the *A*, *B*, *S*, *E*, and *L* descriptors, respectively. The plots of the predicted consensus values versus the experimental solute descriptors are shown in Figure 3.

### 3.4 Model Development of the K-Nearest Neighbors Approach

The kNN model is based on Kühne et al.^45^ and is, in essence, a combination of several kNN submodels. Our implementation of their approach is as follows: The *n* ^th^ submodel first transforms all training molecules into a vector of the counts of their atom-centered fragments’ (ACFs’) of the *n* ^th^ order. To do that, it first converts a molecule’s SMILES string into a graph where nodes represent atoms and edges represent bonds. The graph is then split into as many ACFs as it has nodes, with each node being the center of one ACF. An ACF of the degree *n* with the center atom *a* is a subgraph consisting of *a* and all the neighbors that are a maximum of *n* bonds away from it. We can enumerate all unique ACFs in the training set, and each training and query molecule is represented as a vector, with its i^th^ component being the count of the i^th^ unique ACF in that molecule. Then, for a query molecule, a standard kNN algorithm is applied using the Jaccard distance on the described space, where only the training set neighbors of the query within a certain distance threshold are considered, and their values are combined as a weighted sum of their distances to the query. This way, we have *n* predictions from *n* submodels, combined using a weighted sum to obtain the final prediction. Our implementation can be seen as an extension of the algorithm of Kühne et al.^45^ to any number *n* of submodels, that is, to any maximal ACF order. This involves optimizing the following set of model hyperparameters: the maximal ACF order, the number of nearest neighbors, similarity thresholds, the weights for the submodels, the properties of nodes and edges, and the weight of the logarithmic counting, as described in the original paper.

A kNN model with fixed hyperparameters does not require a validation set, since no parameters are further adjusted. Hence, one can use the same data both for hyperparameter optimization and for training. For hyperparameter optimization, the remaining 90% of the data, after selecting the test set, is temporarily split again into 80% for training the model with candidate hyperparameters and 20% for evaluation. After selecting the optimal hyperparameters, we use the entire training and temporary validation set for model training to reach the best performance of the kNN model. Of course, the approach has, therefore, a slight advantage with respect to the number of training data compared to the other approaches. To evaluate the model, it is applied to the test set (Table 1, Figure 4, SI2–2).

**Figure 4:**
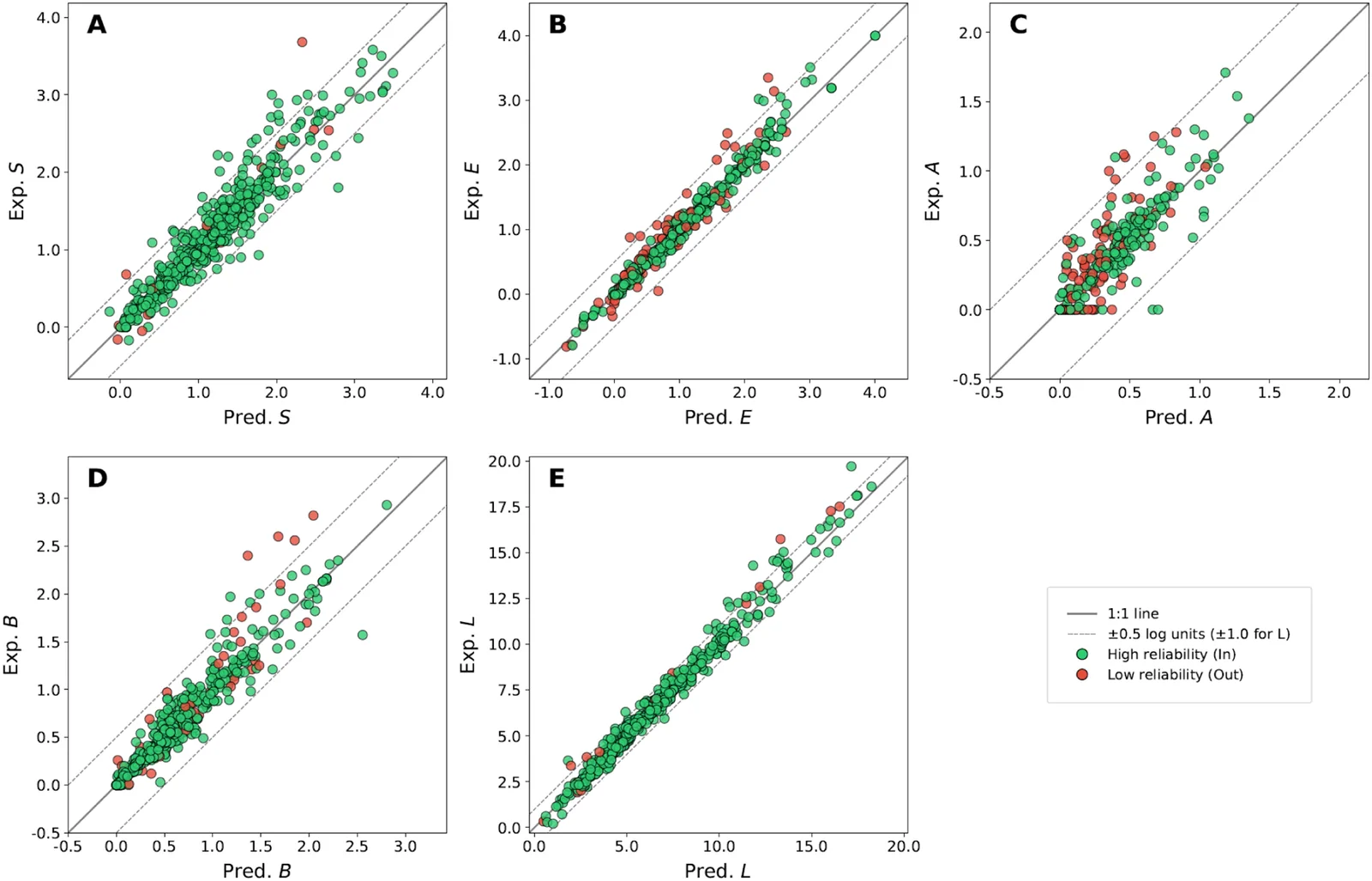
Experimental solute descriptors are plotted against the predicted ones based on the kNN approach for the (A) *S* descriptor, (B) *E* descriptor, (C) *A* descriptor, (D) *B* descriptor, and (E) *L* descriptor. The predictions considered reliable by our approach are marked green, and the unreliable ones are marked red.

To estimate the model’s predictive quality, the approach from Aniceto et al.^66^ was applied after being adjusted for our model. Briefly, this method combines a density approach with information about the bias and spread of submodel predictions. It accounts for the density distribution in the training set by assigning each training compound an individual radius based on the distribution of mean distances to its *k* nearest neighbors. The radii are then adjusted using a correction factor based on the standard deviation of the submodels’ predictions for the corresponding training compounds (spread) and the deviation of the predictions’ mean from the experimental value (bias). A prediction for a query is considered reliable if it is within at least one of those radii. The predictions for the training set were obtained via leave-one-out cross-validation (SI2–2). As the model calculates separate distances for different ACF orders, they were combined into a single distance value via a weighted sum. The relative standard deviations were calculated for each ACF order as the relative standard deviation of the nearest neighbors’ values, then combined via a weighted sum. The biases of separate ACF orders were also combined via weighted sum. At the same time, the weights are the same as those used by the model to combine its submodels predictions. In the original paper, the *k* value is a parameter that must be optimized. As our model uses only a specified number of nearest neighbors for its predictions, this same number was also used for the reliability assessment, rather than optimizing *k*.

The prediction results for the independent test sets of the solute descriptors are shown in Figure 4 and Table 1. The *rmse* values for the *A* descriptor and *B* descriptor are 0.13 and 0.15, respectively. For *S*, an *rmse* value of 0.22 was achieved. The *rmse* of the *E* descriptor is 0.13, and the *rmse* of the *L* descriptor is 0.45.

### 3.5 Model Development of the Graph-Convolutional Neural Network

The GNN model was developed analogously to the GCA model, using five random splits of the training and validation sets. First, several hyperparameters, such as the number of neurons per hidden layer, learning rate, activation functions, and loss functions, were tested. Therefore, the *rmse* values of validation set predictions, compared to the *rmse* of the training set predictions, were plotted for 200 epochs of training, varying the number of neurons per layer and the learning rate. Second, different loss and activation functions were used to determine the best hyperparameters (details can be found in SI1–3). Note that the total number of parameters did not exceed 20% of the data in the training set to avoid over-parameterization and overfitting. Compared to our former model,^40^ we did a Keras implementation in the code for our revised GNN model, the general structure was kept the same. Additionally, we applied the 5-fold split of training and validation set here, to allow for a cross-validation and to obtain a more robust model.

The results of the model performances can be found in SI2–3, where, for each descriptor, the performance of the training, validation, and independent test sets is evaluated for the five different models developed. The *r* ^2^ as well as the *rmse*, bias, and the maximum positive and negative errors, are given. In brief, the *rmse* values for the *S* descriptors range from 0.177 to 0.199, with an average of 0.185 (±0.008) for the validation set, and an average *r* ^2^ of 0.924 (±0.006). The *rmse* values for the test set range from 0.175 to 0.210, with an average *rmse* of 0.187 (±0.012) and an average *r* ^2^ of 0.927 (±0.010). For the *E* descriptor, the *rmse* values across the different validation sets range from 0.086 to 0.107, with an average of 0.096 (±0.007). The average *r* ^2^ is 0.982 (±0.002) for the validation set and 0.083 (±0.001) for the test set. The corresponding *rmse* values range from 0.095 to 0.106 with an average of 0.100 (±0.004). For the *A* descriptor, the average *r* ^2^ of the validation set is 0.910 (±0.007) with *rmse* values ranging from 0.082 to 0.096. The average *rmse* value is 0.087 (±0.005). The test set shows *rmse* values between 0.084 and 0.087 with an average value of 0.086 (±0.001) and an average *r* ^2^ of 0.922 (±0.003). As the training set for the *A* descriptor is imbalanced, we included an additional analysis, whether this imbalance has an impact during model development (which would demand for oversampling, e.g., during model development or tailored loss functions). Details are provided in S1–4. Based on these findings, we could not identify issues related to the imbalanced data set; the corresponding accuracy (distinguishing between *A* = 0 and *A* ≠ 0) is 94.3%. For the *B* descriptor, the validation set *rmse* values range from 0.096 to 0.109 with an average of 0.100 (±0.007) and an average *r* ^2^ of 0.954 (±0.005). The *rmse* values of the test set are between 0.103 and 0.110 with an average *rmse* of 0.106 (±0.003) and an average *r* ^2^ of 0.958 (±0.002). The *rmse* values for the *L* descriptor range from 0.272 to 0.289 in the validation set, with an average of 0.281 (±0.006) and an average *r* ^2^ of 0.992 (±0.001). For the test set, the *rmse* values range from 0.279 to 0.324 (average 0.292 ± 0.017), and the average *r* ^2^ is 0.992 (±0.001). All *rmse* values across the different validation sets for all descriptors are comparable, with a low standard deviation, indicating that the models are robust and there is no dependency on the split used to obtain the corresponding training and validation sets. Furthermore, the results across the different validation sets for the five descriptors are consistent with those obtained for the corresponding independent test set.

The results for the consensus GNN approach are better than those of the individual models, with *rmse* values of 0.08, 0.09, 0.17, 0.09, and 0.25 for the *A*, *B*, *S*, *E*, and *L* descriptors, respectively.

In Figure 5, plots of predicted (consensus) versus experimental solute descriptors are shown. We used the standard deviation (SD) of each consensus value to indicate whether the corresponding value is of high reliability (SD ≤ 0.20; green), moderate reliability (SD ≤ 0.40, yellow), or low reliability (SD > 0.40; red). Due to the *L* descriptor’s larger range, we set SD ≤ 0.30 for high reliability, SD ≤ 0.70 for moderate reliability, and SD > 0.70 for low reliability. As shown in Figure 5, only two values are indicated as having low quality for the *L* descriptor. Most predictions are marked green, indicating they are of high quality and reliability. We showed an interval of ±0.5 to depict deviations from the 1:1 line; most predictions fall within this interval. There are some cases, e.g., in Figure 5E, where data points marked in green are outside the ±0.5 interval; nevertheless, these are only a few cases that demonstrate that the SD serves as a good indicator for the quality of the predictions.

**Figure 5:**
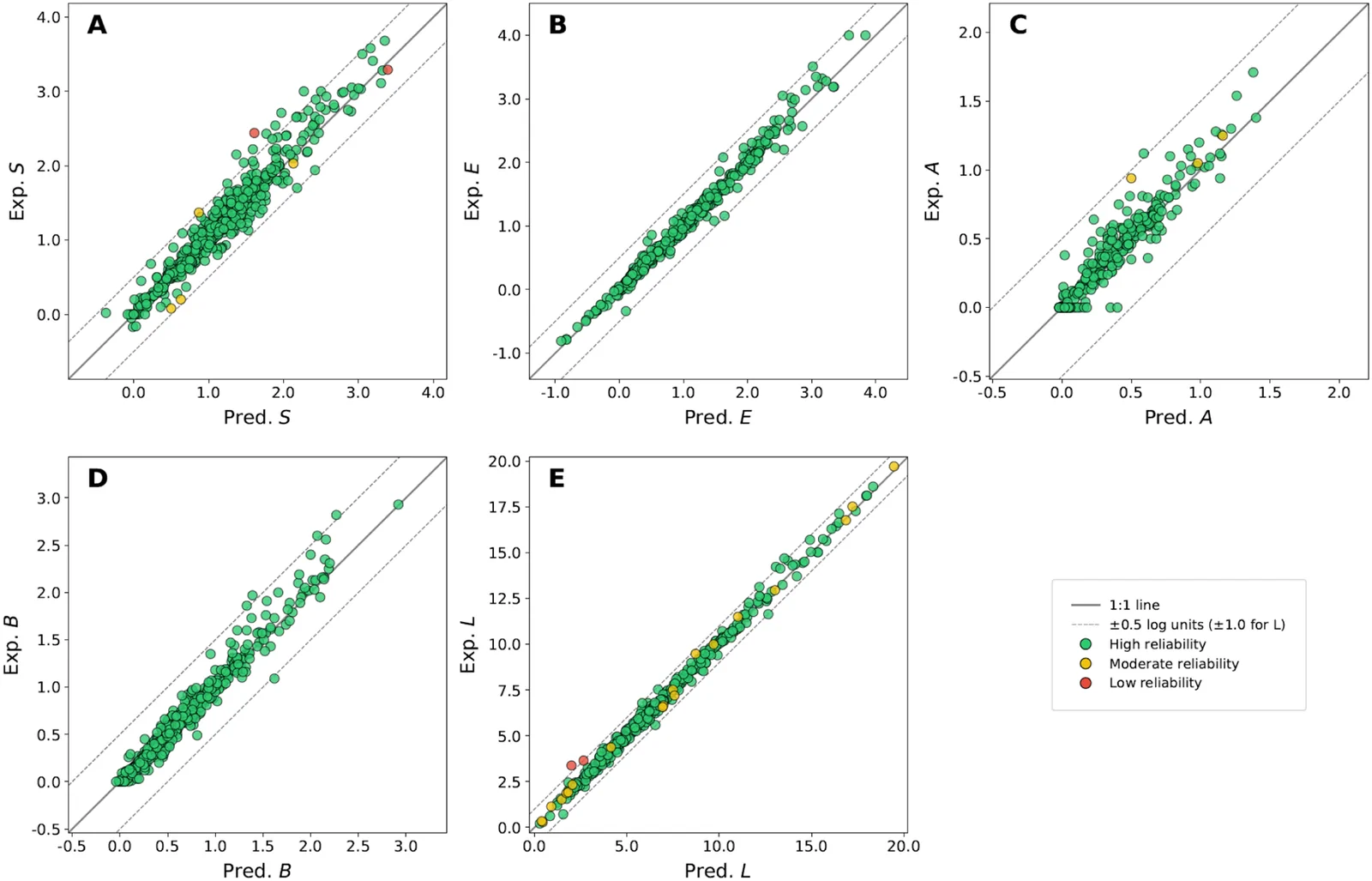
Experimental solute descriptors are plotted against the predicted ones based on the GNN approach for the (A) *S* descriptor, (B) *E* descriptor, (C) *A* descriptor, (D) *B* descriptor, and (E) *L* descriptor. Note that the quality and reliability of the prediction are depicted by the color: green: high reliability, yellow: moderate reliability, and red: low reliability.

### 3.6 Comparison of the Performance of the Three Different Approaches

We used the test set predictions for all descriptors to compare the performance of the three models. Results can be found in Table 1; more details on the individual test set predictions are provided in SI2–4. It should be noted that the *rmse* values reported are derived from the consensus predictions of the five models developed for GCA and GNN.

In general, the results from the GCA and kNN predictions are comparable. In the cases of the *A*, *B*, and *L* descriptors, the corresponding GCA model performs slightly better than the kNN model (*rmse* of 0.12 vs 0.13 for the *A* descriptor, 0.14 vs 0.15 for the *B* descriptor, and 0.42 vs 0.45 for the *L* descriptor). For the *S* and *E* descriptors, the kNN approach performs slightly better than the GCA approach (0.22 vs 0.23 for the *S* descriptor, and 0.13 vs 0.19 for the *E* descriptor). However, for most of the solute descriptors (*S*, *A*, and *B*), the difference might not be significant in the application of the LSER models. The predictive performance of the GNN models for each descriptor is better than that of kNN and GCA. Especially for the *L* descriptor, the *rmse* could be significantly reduced to 0.25 compared to 0.42 (GCA) and 0.45 (kNN). Furthermore, the corresponding *rmse* values for the other descriptors are lower as well, with *rmse* values < 0.1 for *A*, *B*, and *E* descriptors and an *rmse* value of 0.17 for the *S* descriptor.

We conducted a deeper analysis on the test set predictions for all three approaches to ensure their physical consistency. We investigated correlations of the *E* and *L* descriptor as well as *E* and *S* descriptor (SI1–5). Additionally, we applied trend analyses for specific functional groups for the *A* and *B* descriptors (SI1–6), and for homologous series for the *L* and *E* descriptor (SI1–7). The trends observed in the experimental data are correctly captured by all three models, demonstrating that the predictions follow the expected intermolecular interaction patterns and are not merely a result of statistical fitting. This leads to consistent predictions for chemicals with various functional groups and homologous series. On top, we applied a subgroup analysis for the test set chemicals, details are provided in SI2–5. The kNN and GNN models show a decrease in prediction performance for the subgroup of more heterogeneous chemicals across all descriptors. Moreover, for the kNN model, N- and/or O-containing chemicals are predicted slightly worse than those containing only C and H, and halogens are predicted worse than other groups. As these simple structures are overrepresented in the data set, this is plausible. For the GCA approach, different trends can be seen: For *A*, *B*, and *S*, the *rmse* is slightly higher for chemicals containing C, H, N, and O than for those containing only C and H. This is due to the fact that such functional groups (e.g., NH_2_, OH, CHO) often exhibit specific intermolecular interactions and neighboring-group effects, which strongly affect the *A*, *B*, and *S* descriptors. Such interactions cannot be captured by the additive scheme of the GCA approach. In contrast, when only C and H atoms are present, the corresponding descriptor values are usually zero (or close to zero), which is a trend easily reproducible by the GCA approach, thus leading to a decreased *rmse* for these compounds.

For the *E* and *L* descriptors, where the size of the molecule strongly affects the descriptors, the GCA model performs better for systems with CHNO functional groups, where the size of a system can be described by a simple additive scheme. If the system only contains C and H atoms, the molecule often folds and branches in complex ways, which cannot be captured by GCA and thus leads to increased *rmse*. For the halogenated compounds, the GCA approach shows a decreased *rmse* for every single descriptor except *L*, where it is on par with the overall *rmse*.

Comparing the results of the test set to the outcomes of our former study,^40^ it can be seen that the consensus-based GNN approach performs significantly better compared to the former GNN models (*rmse* values of 0.12 (*E*), 0.22 (*S*), 0.11 (*A*), 0.14 (*B*), and 0.42 (*L*)). However, the *rmse* values are in the same range for the GCA and kNN models. Note that the same test sets were used. Further, the QSPR method implemented in LSERD and ACD/Absolv did not show significant differences in the overall performance for the old model, with *rmse* values of 0.14 (*E*), 0.28 (*S*), 0.09 (*A*), 0.13 (*B*), and 0.52 (*L*) for the QSPR model implemented in LSERD and 0.10 (*E*), 0.23 (*S*), 0.09 (*A*), 0.16 (*B*), and 0.44 (*L*) for the QSPR model implemented in ACD/Absolv.^40^

### 3.7 Consensus-Based Approach of the Three Different Models

For the consensus-based approach, we integrated the predictions from GCA, kNN, and GNN. It should be clarified that for both GCA and GNN, we utilized the preconsolidated consensus outputs of five distinct model iterations. To evaluate whether a consensus-based approach across the three types of models developed would improve overall prediction of solute descriptors, we first calculated consensus values for the test set chemicals and the five solute descriptors, along with their corresponding standard deviations (SDs).

We used boxplots (Figure 6) to depict the results of the different test sets for each model and the consensus results. Details are provided in SI2–6 and SI1–8. The median absolute difference between the experimental and predicted values for the *S* descriptor is lowest for the GNN model (0.06), followed by the consensus model (0.07). The kNN model shows a slightly higher median of 0.08, and the GCA model shows a median of 0.10. The median absolute differences for the *E* descriptor are 0.03 for the GNN and the kNN model, the GCA model shows a median absolute difference of 0.08, and the consensus approach shows a median absolute difference of 0.04. For the solute descriptor *A*, the median absolute difference between the experimental and the predicted value is 0.01 for the GNN, the kNN, and the consensus model. The GCA model shows a slightly higher median difference of 0.02. The median absolute differences for the *B* descriptor are 0.03 for the GNN model, 0.04 for the kNN and consensus approach, and 0.07 for the GCA model. For the *L* descriptor, the median absolute differences are higher with 0.11 (GNN), 0.18 (kNN), 0.22 (GCA), and 0.15 (consensus approach). In general, the median absolute difference of the consensus approach is not lower; often, the GNN model shows more accurate results. This is due to the higher error caused by the GCA and/or the kNN model.

**Figure 6:**
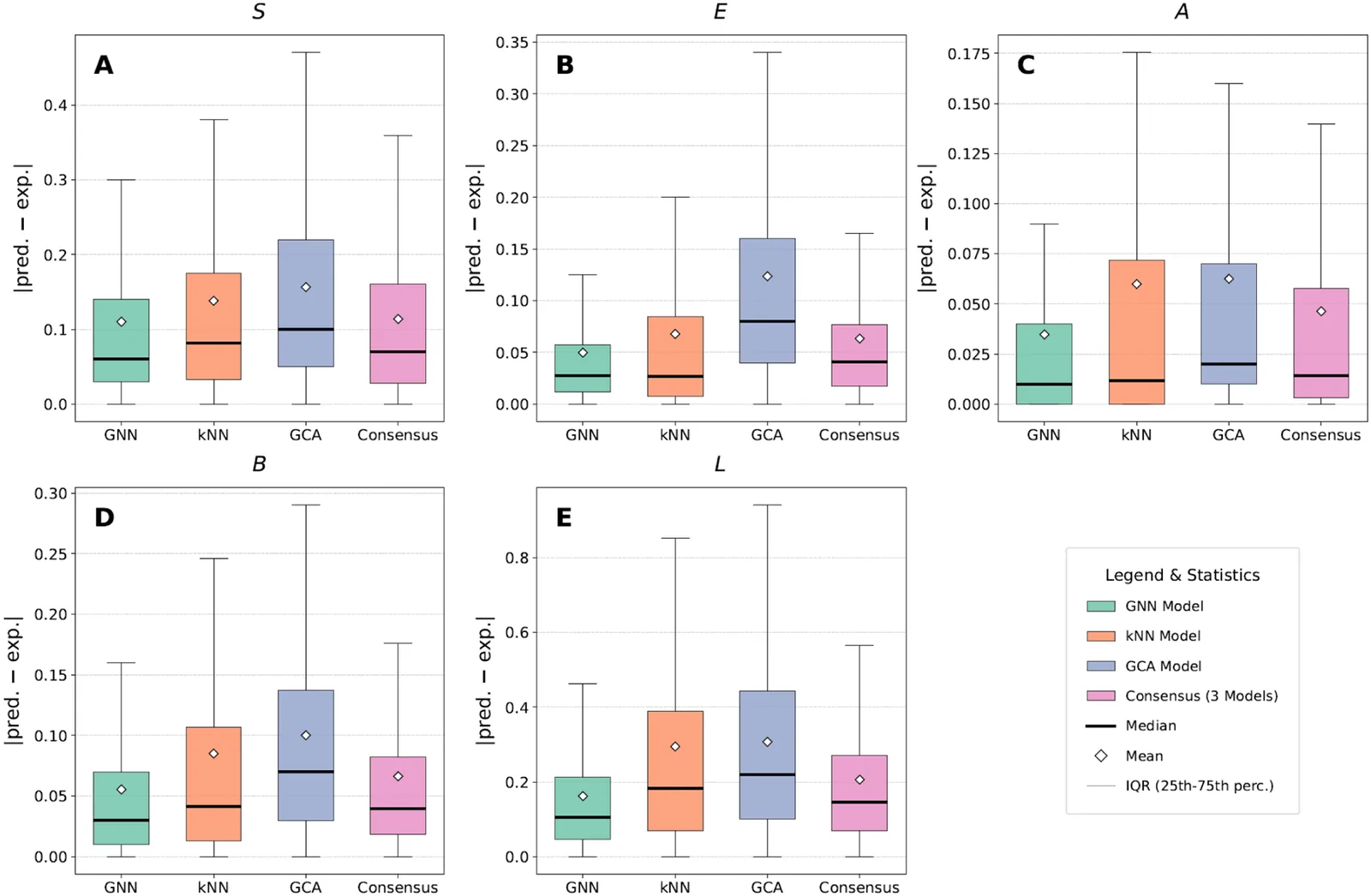
Boxplots to compare the absolute differences between predicted and experimental values for the three models and the consensus approach for (A) the *S* descriptor, (B) the *E* descriptor, (C) the *A* descriptor, (D) the *B* descriptor, and (E) the *L* descriptor.

Furthermore, the SD of the consensus value could serve as an additional indicator to determine whether a consensus approach would improve overall prediction of the corresponding solute descriptor (Figure 7). We therefore used the 33rd percentile of the SD of the consensus values to define high-confidence values, the 66th percentile to define mid-confidence values, and the remaining values as low-confidence values. Details on the outcomes for the individual solute descriptors are available in SI2–7. In brief, for the *S* descriptor, 210 of 635 consensus predictions are declared high confidence, with an *rmse* of 0.08 (compared to 0.168 for all). For *E*, the number of high-confidence predictions is 209 (out of 634) with an *rmse* of 0.039 (compared to 0.103). For the *A* descriptor, the *rmse* of the 228 high-confidence predictions (out of 634) is 0.009 (compared to 0.087), and for the *B* descriptor, 0.052 (compared to 0.106, 200 out of 606 chemicals). For *L*, 181 high-confidence consensus predictions were performed (out of 548), with an *rmse* of 0.144 (compared to 0.290).

**Figure 7:**
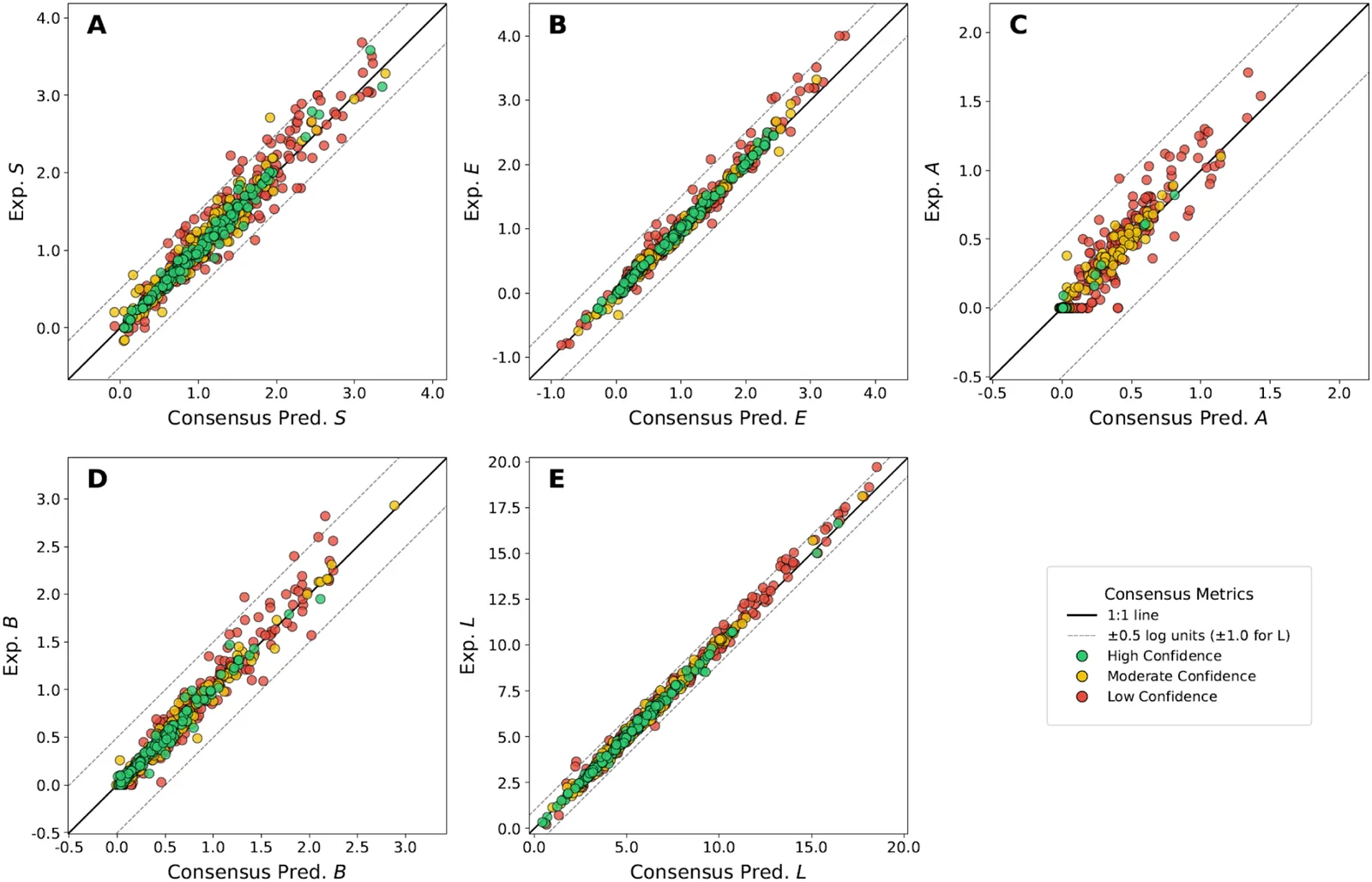
Indication of high-confidence consensus predictions for the test set chemicals. The experimental solute descriptor is plotted against the predicted consensus solute descriptor. The solute descriptors *S* (A), *E* (B), *A* (C), *B* (D), and *L* (E) are shown separately.

Currently, a consensus-based approach (including different types of models) is often preferred.^67^ In the case of solute descriptor predictions (for our independent test set), it becomes clear that the errors made by each model in the consensus approach strongly affect its overall performance. In our case, only ∼30% of the chemicals could be predicted with high confidence. However, it is hard to judge whether the prediction improves for a given chemical. Further, it should be noted that there may still be errors in the experimental solute descriptor, which could lead to misinterpretation of the predictive outcomes of one or more models. Thus, clear trends indicating a favorable use of one model over the others or a consensus approach cannot be observed.

We therefore decided to apply solute descriptor predictions in LSER equations to investigate the overall effects of individual differences in prediction performance and to see whether a consensus approach would enhance the prediction of physicochemical properties. We selected three partition coefficients, which are relevant for the risk assessment of chemicals: log *K*_ow_, the octanol-air partition coefficient (log *K*_oa_), and the water–air partition coefficient (log *K*_wa_), where experimental data were available to compare the outcomes of the LSER predictions. Further, we included two retention indices: the Kovats retention index (*KRI*) for gas chromatography applications^28^ and the chromatographic hydrophobicity index *CHI* for reversed-phase liquid chromatography applications.^30^ The experimental data sets were already used in our former study^40^ and enable direct comparisons with results from ACD Absolv and LSERD solute descriptor predictions, as well as with our former GNN model (DNN_taut_). The results are shown in Table 2.

**Table 2:** Solute Descriptor Predictions are Used to Estimate Partition Coefficients and Retention Indices Using LSER Equations^a^

Parameter	*N*	Consensus	GNN	GCA	kNN	DNN_(taut)_	QSPR LSERD	ACD Absolv
log *K*_ow_	12,010	1.03	1.09	1.06	1.65	1.04	0.91	0.87
log *K*_oa_	270	0.37	0.36	0.57	0.59	0.63	0.49	0.59
log *K*_wa_	696	1.32	1.13	1.91	1.30	1.36	1.28	1.39
*KRI*	454	129	109	157	160	129	104	120
*CHI*	204	3.80	3.64	5.38	4.41	4.49	5.52	3.19

a The *rmse* values for the predictions of the octanol-water partition coefficient (log *K*_ow_), octanol-air partition coefficient (log *K*_oa_), air-water partition coefficient (log *K*_wa_), and the Kovats retention index (*KRI*), as well as the chromatographic hydrophobicity index (*CHI*), are predicted. Note that log *K* values are reported as dimensionless. Although units cancel out in the base calculation (e.g., c_octanol_/c_water_), it still accounts for the respective phase volumes. Both retention indices are dimensionless. For solute descriptor calculation, the GCA, kNN, GNN, and the consensus approach are applied. Additionally, the prediction results of our former study are shown for comparison.

From our perspective, it is crucial to account for the experimental error in the corresponding physicochemical properties. Usually, one would expect an experimental error for log *K*_ow_ determination in the range of 0.2–0.4 log units. The experimental errors for log *K*_oa_ and log *K*_wa_ might be in the same range or even higher, as log *K*_wa_ and log *K*_oa_ cover a larger range of log values. Having a closer look at the predictions from the log *K*_ow_, consensus, GNN, and GCA approaches, as well as the former DNN model, the results are comparable. The QSPR integrated in LSERD and ACD Absolv are slightly better. Only the kNN model predictions seem worse, which is reasonable given the approach itself, since the total number of training set chemicals used in the model is >50% of the chemicals included in the experimental log *K*_ow_ data set. For log *K*_oa_, the consensus approach and the GNN perform best, whereas the kNN and GCA approaches are in line (also with the DNN model and ACD Absolv; only the QSPR model of LSERD is slightly better). For the log *K*_wa_, the GNN model performs best, whereas the GCA approach delivers results marginally worse than those of all other approaches. For the retention indices *KRI* and *CHI*, the GNN performs better than the GCA and kNN approaches. Again, the consensus approach is also slightly better than the GCA and kNN approaches. For *KRI*, the lowest rmse was obtained with the QSPR approach of LSERD, and for *CHI*, the ACD Absolv approach. Nevertheless, there is no clear trend of one model performing better than the others, although the consensus approach seems to deliver stable, relatively accurate predictions.

## 4 Conclusions

We developed three models to predict solute descriptors using GCA, kNN, and GNN approaches. The three models for the prediction of LSER descriptors are implemented in the PAULY software (https://i-am-pauly.com/). The three approaches can be applied individually or combined as a consensus approach. Nevertheless, one should be aware that the models included in the consensus approach affect prediction quality through their individual prediction errors, which might lead to shifts or higher errors in consensus predictions. Using our independent test set, we demonstrated that one should be aware of the corresponding SDs of the predicted values, which can serve as a quality indicator for the calculated solute descriptors. Especially for the consensus approach, it is essential to consider the SDs to avoid larger prediction errors introduced by one of the three models. Still, the applicability domain is one of the LSER approach’s most considerable disadvantages, as it primarily covers relatively simple chemical structures, given the available experimental solute descriptors. This should be considered when applying the respective solute descriptors in LSER equations to receive partition coefficients, for example.

On top of that, many LSER equations are developed using fewer than 200 chemicals, further narrowing their applicability domain. Another shortcoming is the lack of a consistent approach to ionogenic chemicals, as ionic interactions are not accounted for in most equations used to predict physicochemical properties. Thus, direct predictions of solvation energies^68^ or activity coefficients^69,70^ by NNs might be useful alternatives, especially for such chemicals. Furthermore, physics-based simulations offer high mechanistic accuracy and intrinsic transferability, while the LSER approach provides a more efficient alternative for high-throughput screening for applications in environmental science. Within this framework, temperature dependencies are explicitly captured through the reparameterization of the phase parameters (*e*, *s*, *a*, *b*, *l*, *v*, *c*), while the solute descriptors are treated as temperature-independent molecular constants. Nevertheless, our experience with the LSER approach has shown that reliable predictions of physicochemical properties are possible.

## Supplementary Material





## Data Availability

All models are implemented in the PAULY software found at https://i-am-pauly.com/. The original data set is available on GitHub: https://github.com/nadinulrich/solute_descriptor_prediction.

## Notes

Coverage of the chemical space (SI1–1); parameter ranges of the consensus training of the GCA approach (SI1–2); hyperparameter optimization of the GNN models (SI1–3); *A* descriptor prediction by the GNN model (SI1–4); correlation of the solute descriptors for the test set predictions (SI1–5); trends in *A* and *B* descriptor prediction (SI1–6); homologous series predictions (SI1–7); consensus approach predictions (SI1–8) (PDF)
Individual results of the GCA models (SI2–1); leave-one-out cross-validation of the kNN approach (SI2–2); individual results of the GNN models (SI2–3); test set predictions done by all models (SI2–4); details on test set predictions (SI2–6); consensus boxplot results (SI2–7); analysis of the consensus results (SI2–8) (XLSX)

## #

ETH Zürich, Disordered Materials, Vladimir-Prelog-Weg 1-5/10, 8093 Zürich, Switzerland
