# Supplementary material for: Prediction of Solute Descriptors for Linear Solvation Energy Relationships Using K‑Nearest Neighbors, Group Contributions, and Graph-Convolutional Neural Networks
Source: ACS Environ Au. 2026 Jun 10;6(5):851–63. doi: 10.1021/acsenvironau.6c00063 (PMC13602332; doi:10.1021/acsenvironau.6c00063)
Supplement: Supplementary file 1 [file vg6c00063_si_001.pdf]

SI1 - Prediction of solute descriptors for linear solvation energy relationships using k-nearest neighbors, group contributions, and graph-convolutional neural networks

Nadin Ulrich[1,2,*], Valentin Istomin[1,2,3], Anton Kudria[1,2], Alexander Böhme[1], Karsten Voigt[2]

[1]Department of Exposure Science, Helmholtz Centre for Environmental Research – UFZ, Permoserstrasse 15, D-04318 Leipzig, Germany

[2]PAULY, Essener Straße 100, D-04357 Leipzig, Germany

[3]Present address: ETH Zürich, Disordered Materials, Vladimir-Prelog-Weg 1-5/10, 8093 Zürich, Switzerland

*Corresponding Author: Department of Exposure Science, Helmholtz Centre for Environmental Research – UFZ, Permoserstrasse 15, D-04318 Leipzig, Germany

E-mail: nadin.ulrich@ufz.de

Table of Content

SI1-1. Application of models and coverage of the chemical space. 2

SI1-2. Details on parameter ranges of the consensus training of the GCA approach. 3

SI1-3. Hyperparameter optimization for the GNN models.. 5

SI1-4. Detailed investigation of the GNN model predictions of the *A* descriptor. 21

SI1-5. Detailed analysis of test set predictions: correlation of solute descriptors...... 22

SI1-6. Detailed analysis of test set predictions: trends in *A* and *B* descriptors for specific functional groups. 23

SI1-7. Detailed analysis of test set predictions: trends for homologous series for *L* and *E* descriptor.. 24

SI1-8. Consensus approach for the prediction of solute descriptors......................... 25

References 25

SI1-1. Application of models and coverage of the chemical space.

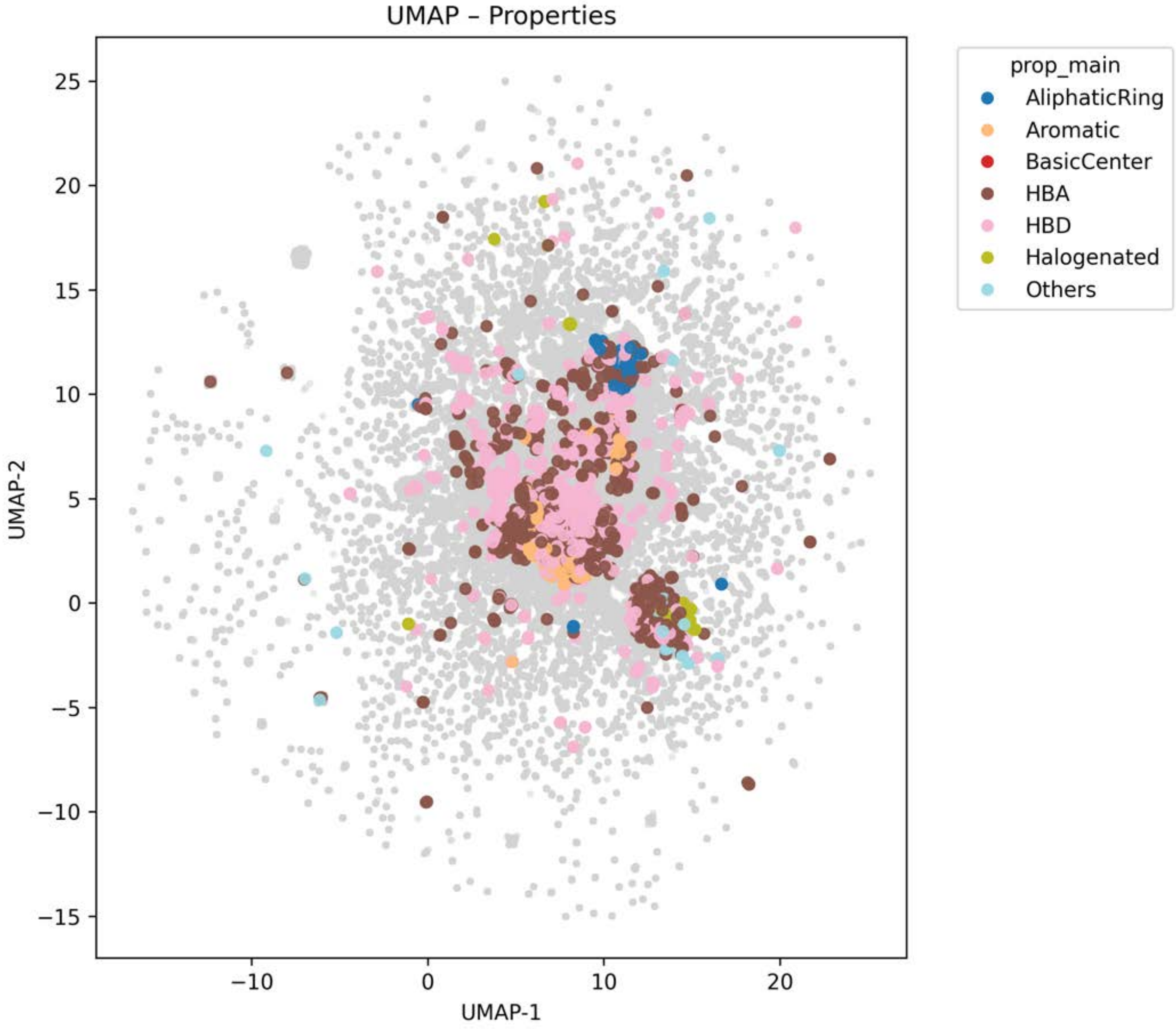

Figure SI1-1. UMAP plot, the PubChemLite database represents the chemical space in light grey, the solute descriptor S is used to demonstrate the coverage of the experimental solute descriptor values in comparison to the overall chemical space. Note that this is a non-linear manifold projection, distances reflect similarities in the corresponding chemical structure. Different chemical properties are marked according to the color legend.

SI1-2. Details on parameter ranges of the consensus training of the GCA approach.

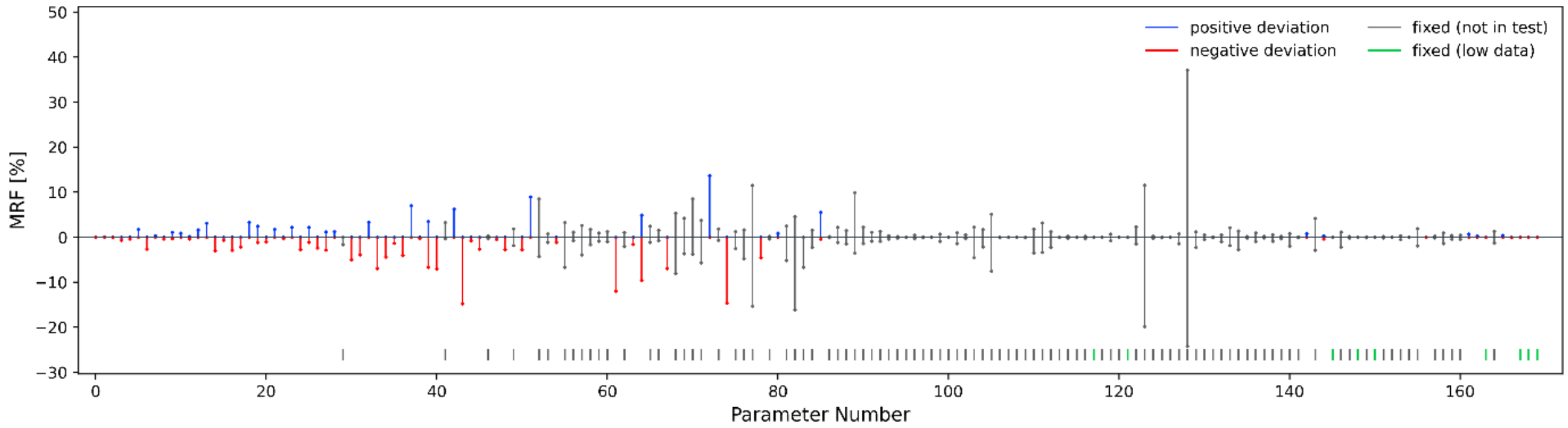

Figure SI1-1. Details on the parameter ranges of the consensus training of the GCA model for the solute descriptor *A*.

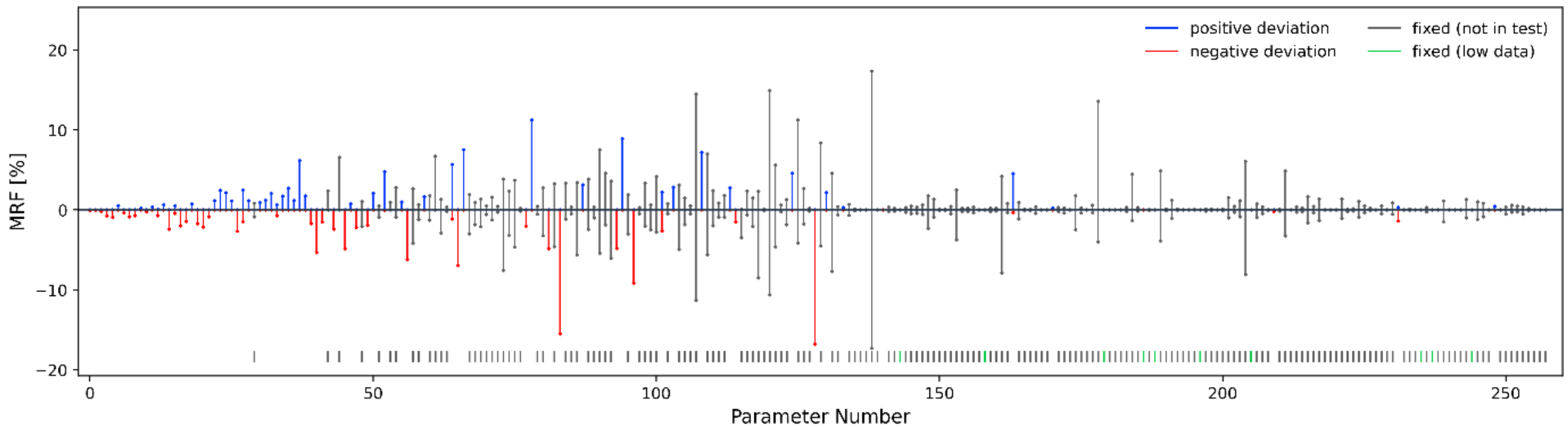

Figure SI1-2. Details on the parameter ranges of the consensus training of the GCA model for the solute descriptor *B*.

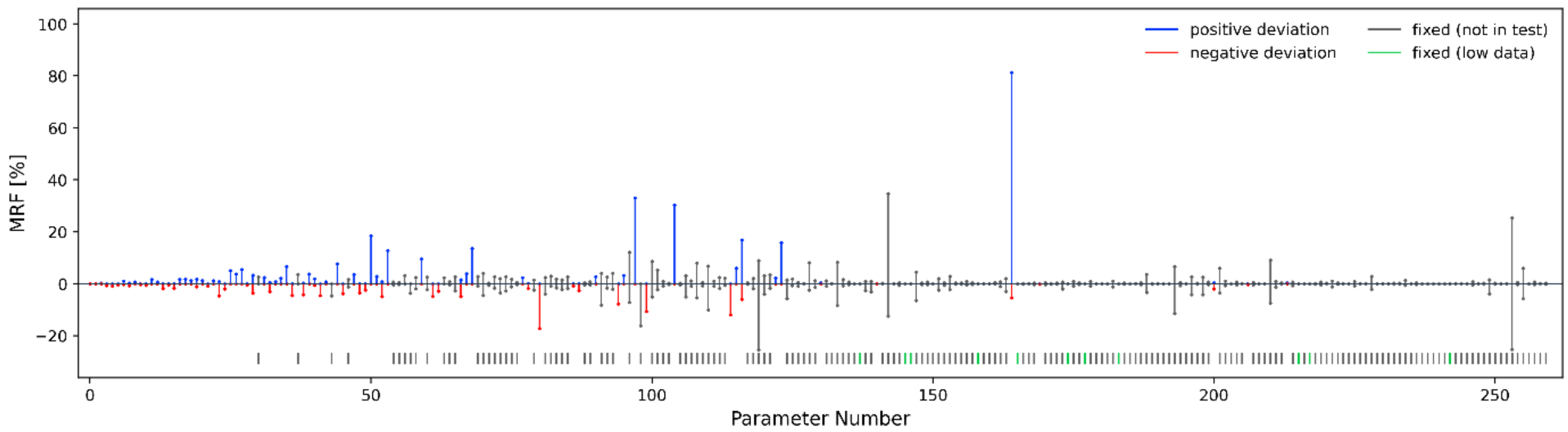

Figure SI1-3. Details on the parameter ranges of the consensus training of the GCA model for the solute descriptor *S*.

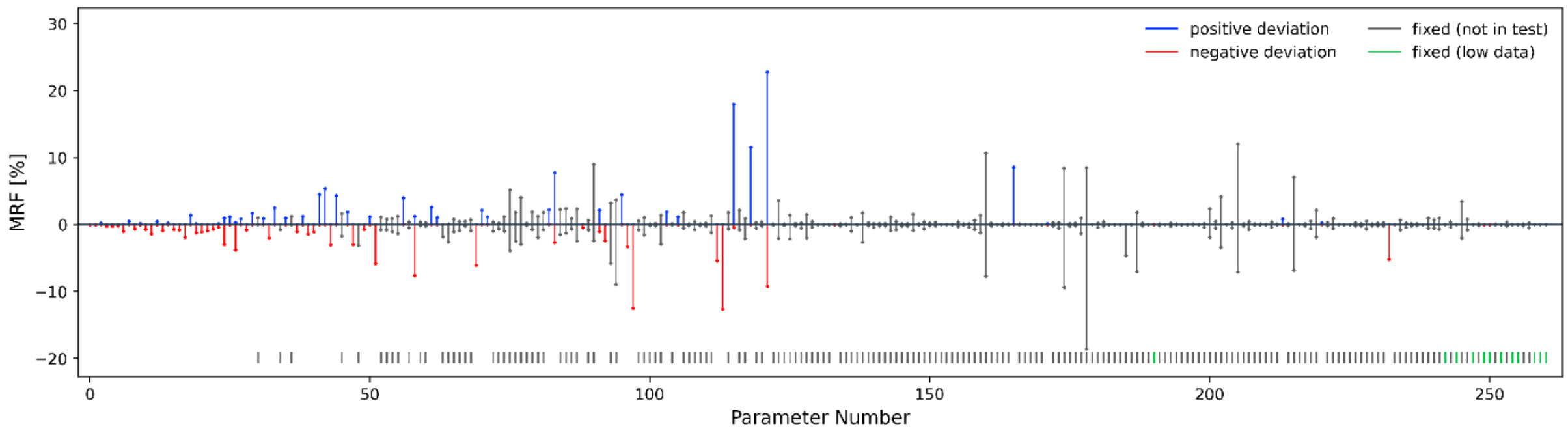

Figure SI1-4. Details on the parameter ranges of the consensus training of the GCA model for the solute descriptor *E*.

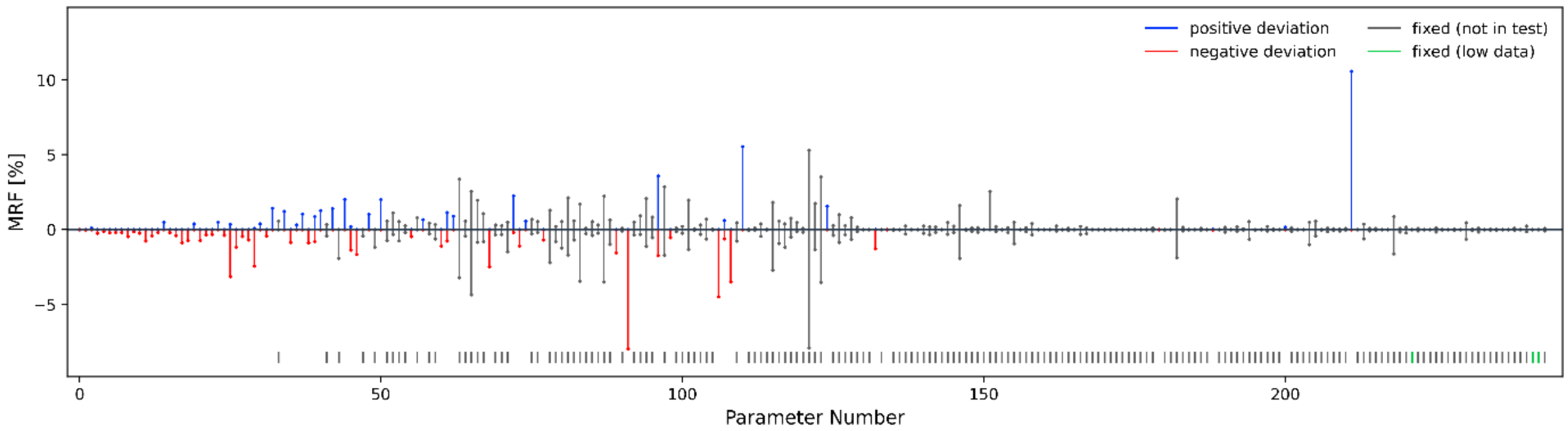

Figure SI1-5. Details on the parameter ranges of the consensus training of the GCA model for the solute descriptor *L*.

SI1-3. Hyperparameter optimization for the GNN models.

For hyperparameter optimization, we initially limited the GNN's parameter count to 20% of the input features. The number of input features was increased using a data augmentation strategy. We, therefore, generated all potential tautomeric forms and SMILES variants for the training set chemicals. Doublings were removed. In our case, we used two hidden layers in our architecture and tested setups with different neurons per hidden layer. In detail, we included the following combinations: (64,64), (64,32), (32,64), and (32,32) neurons, and applied different learning rates for each combination. Additional combinations with lower numbers were not tested, as we had already identified the optimal number of neurons per layer for each of the individual GNNs developed. We further selected different activation and loss functions to identify the optimal activation and loss functions for the corresponding GNN. We applied this approach to each descriptor individually; however, the optimal setup was almost identical across the different descriptors, while the number of training epochs varied. We want to point out that a multitask model was not convincing (we tested this previously)[1], as the dataset was severely limited by the scarcity of data for the *L* descriptor. We included some of the graphs generated from our hyperparameter optimization below for all solute descriptors.

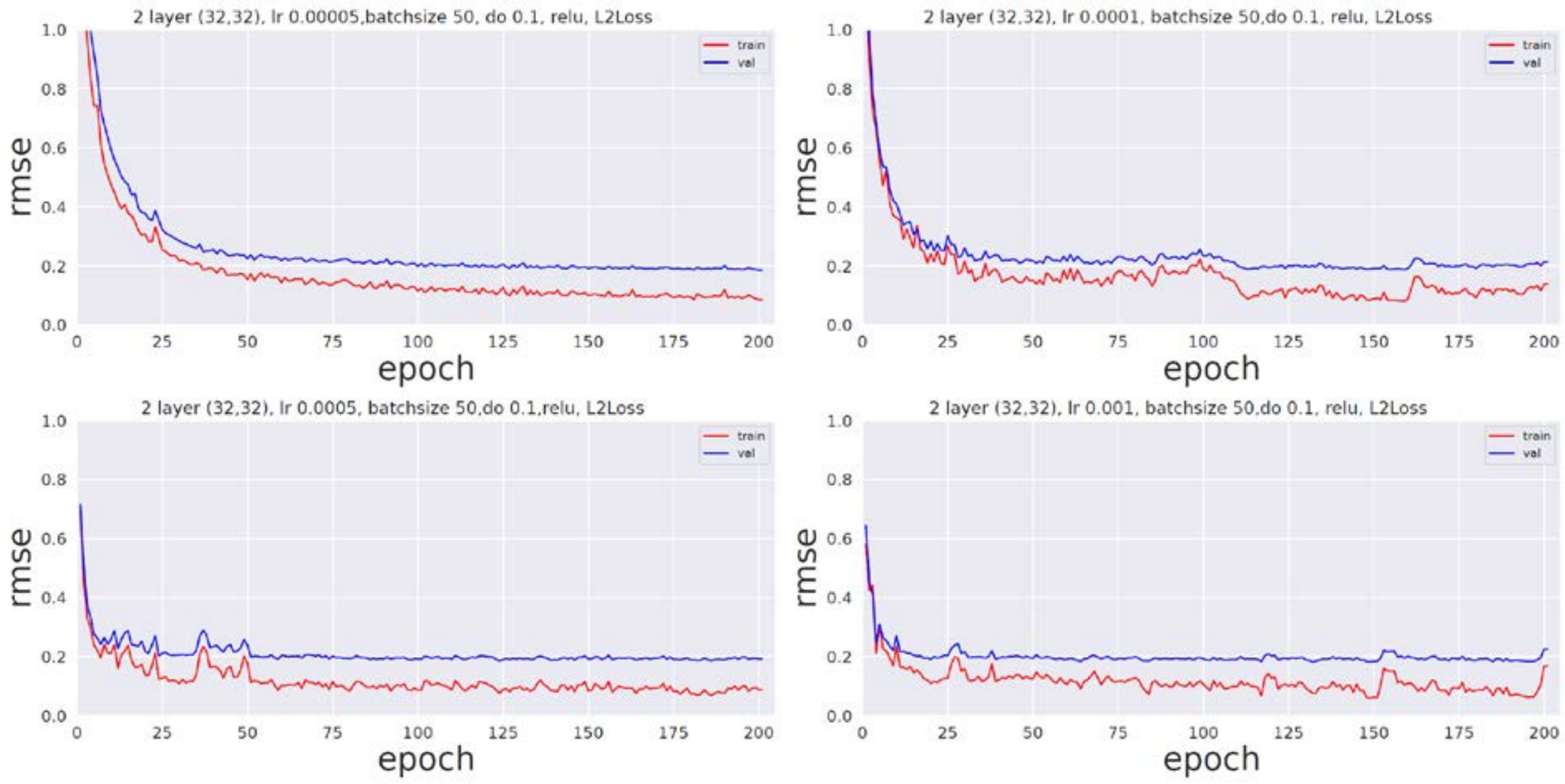

Figure SI1-6. Details on the hyperparameter optimization to find the optimal architecture and setup for the GNN to predict the *S* descriptor. Here, 32 neurons per layer were included, and the learning rate was varied. ReLU and L2Loss functions were set to allow a direct comparison of learning rates. Note that we tested more learning rates than depicted here.

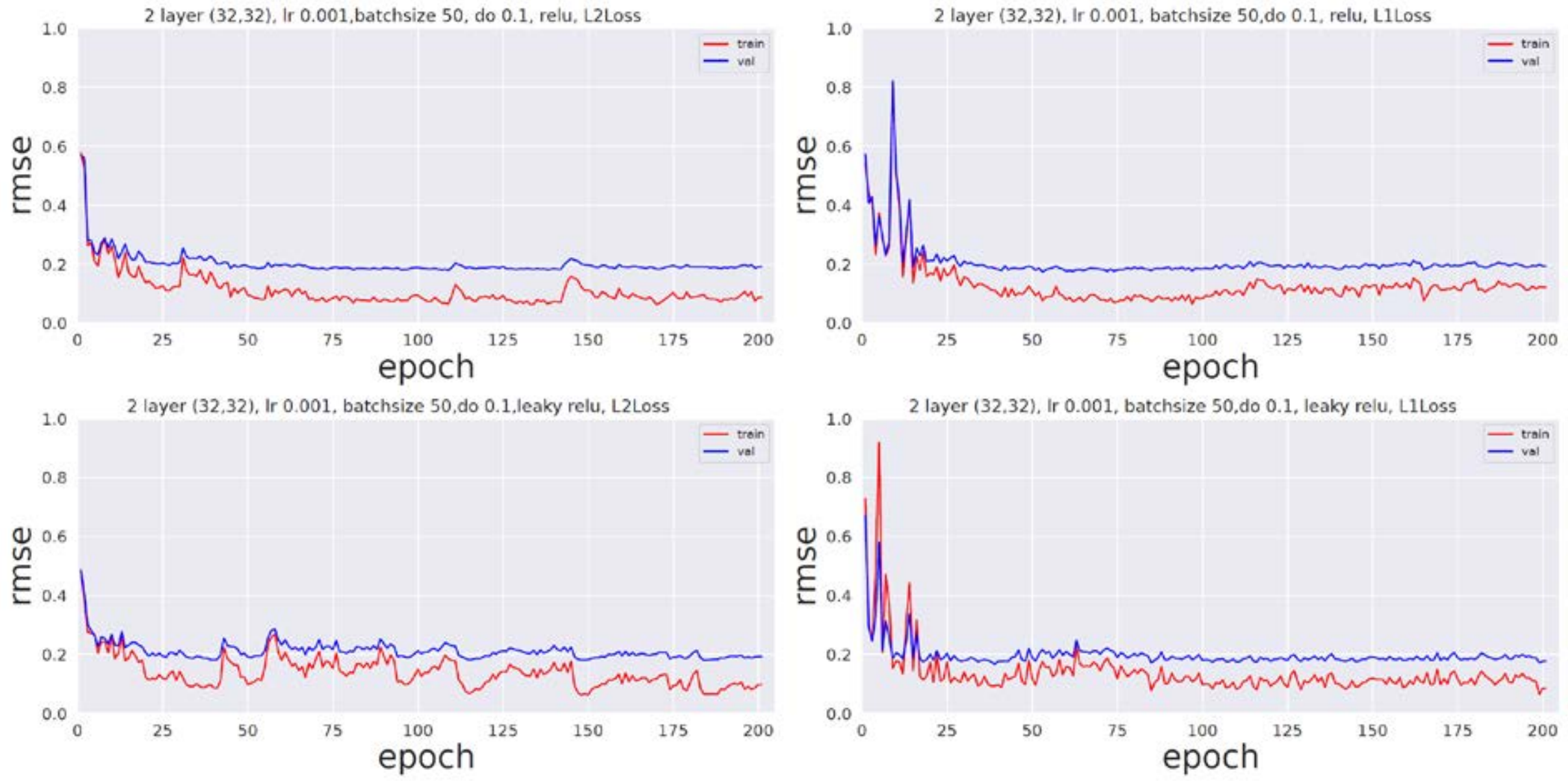

Figure SI1-7. Details on the hyperparameter optimization to find the optimal architecture and setup for the GNN to predict the *S* descriptor. For the GNN including 32 neurons per layer and the identified optimal learning rate of 0.001 different activation and loss functions were tested.

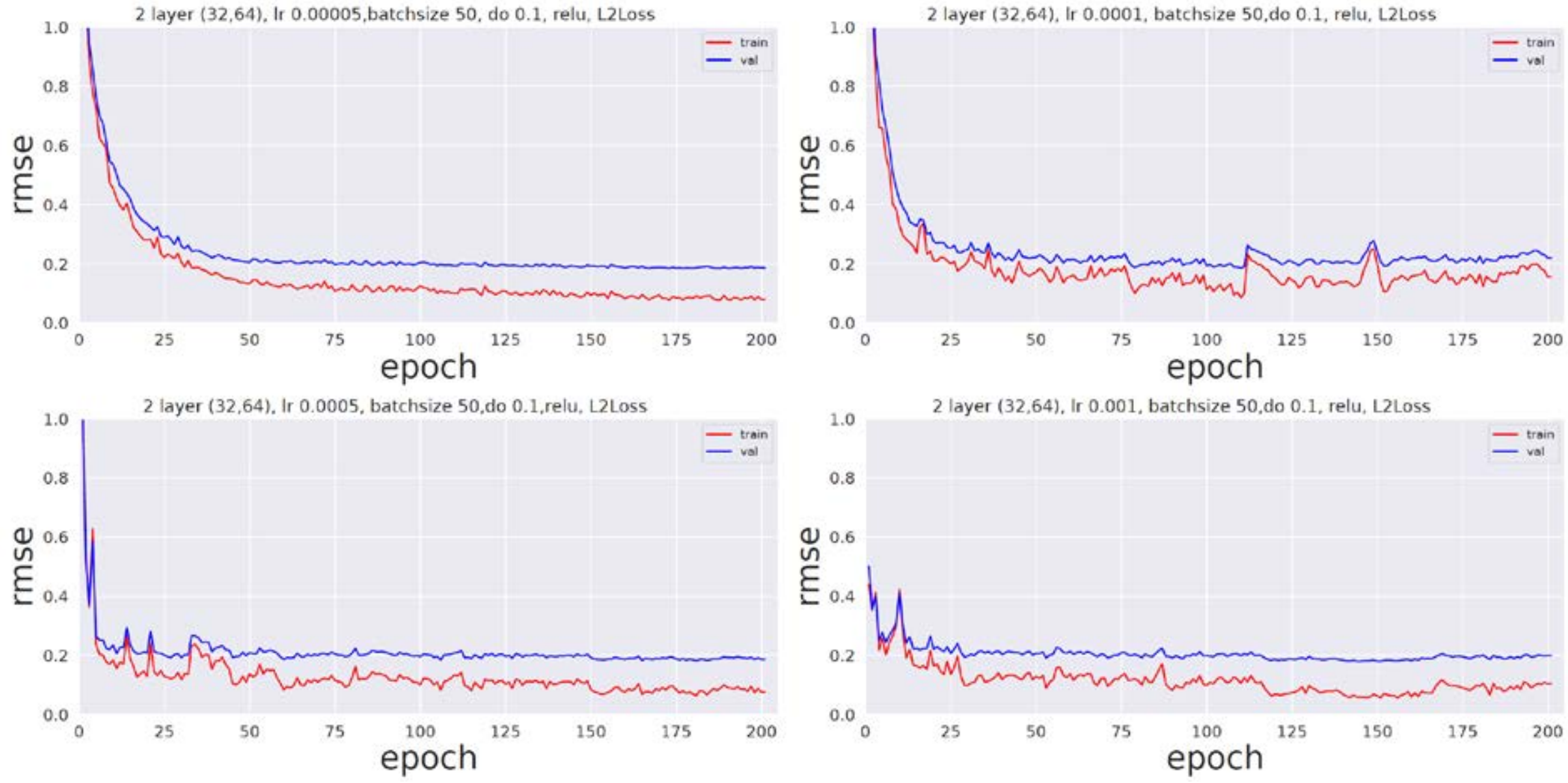

Figure SI1-8. Details on the hyperparameter optimization to find the optimal architecture and setup for the GNN to predict the *S* descriptor. Here, 32 and 64 neurons are included in the two hidden layers and the learning rate was varied. ReLU and L2Loss functions were set to allow a direct comparison of learning rates. Note that we tested more learning rates than depicted here.

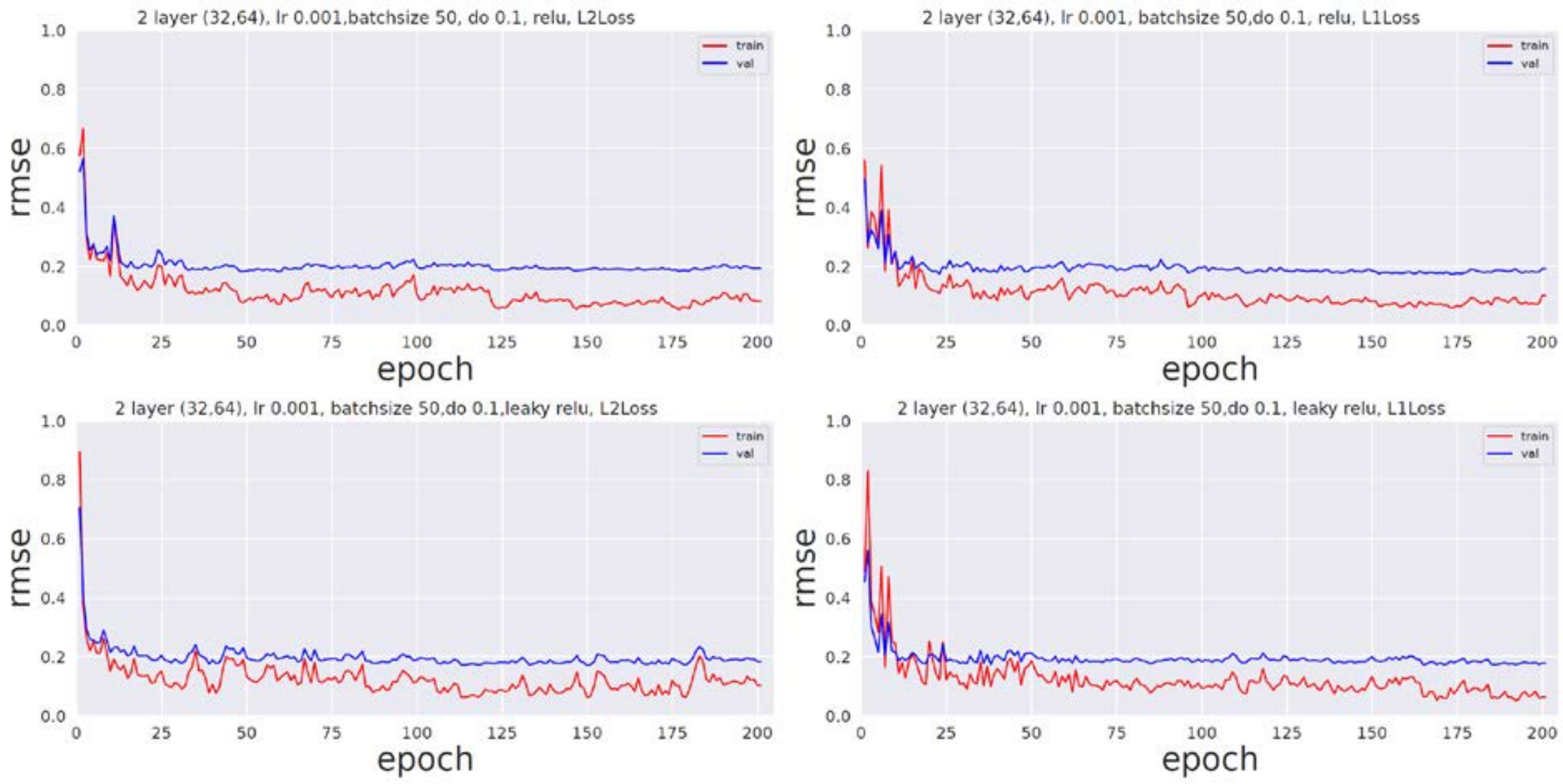

Figure SI1-9. Details on the hyperparameter optimization to find the optimal architecture and setup for the GNN to predict the *S* descriptor. For the GNN including 32 and 64 neurons per layer, respectively, and the identified optimal learning rate of 0.001, different activation and loss functions were tested.

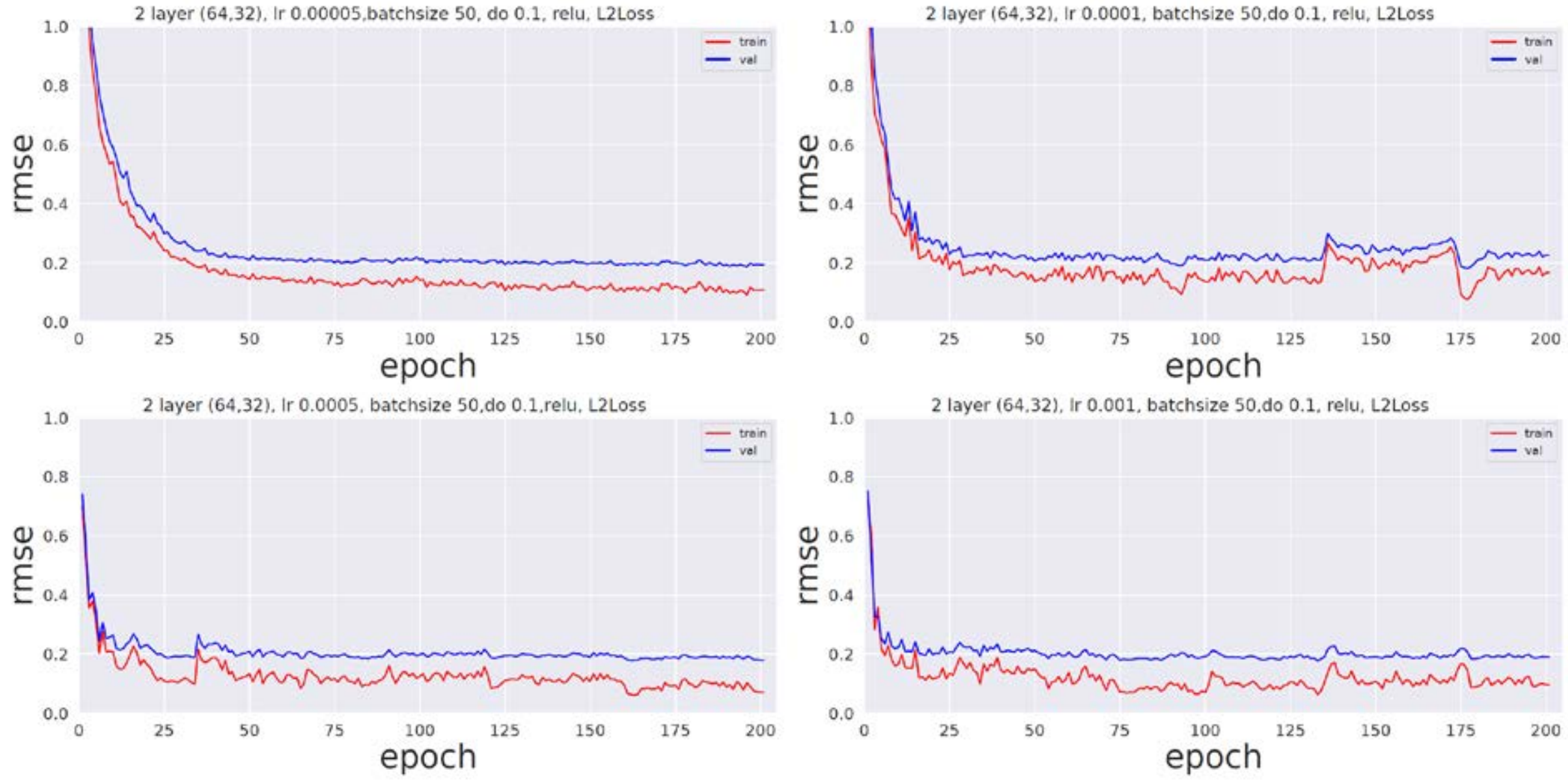

Figure SI1-10. Details on the hyperparameter optimization to find the optimal architecture and setup for the GNN to predict the *S* descriptor. Here, 64 and 32 neurons are included in the two hidden layers and the learning rate was varied. ReLU and L2Loss functions were set to allow a direct comparison of learning rates. Note that we tested more learning rates than depicted here.

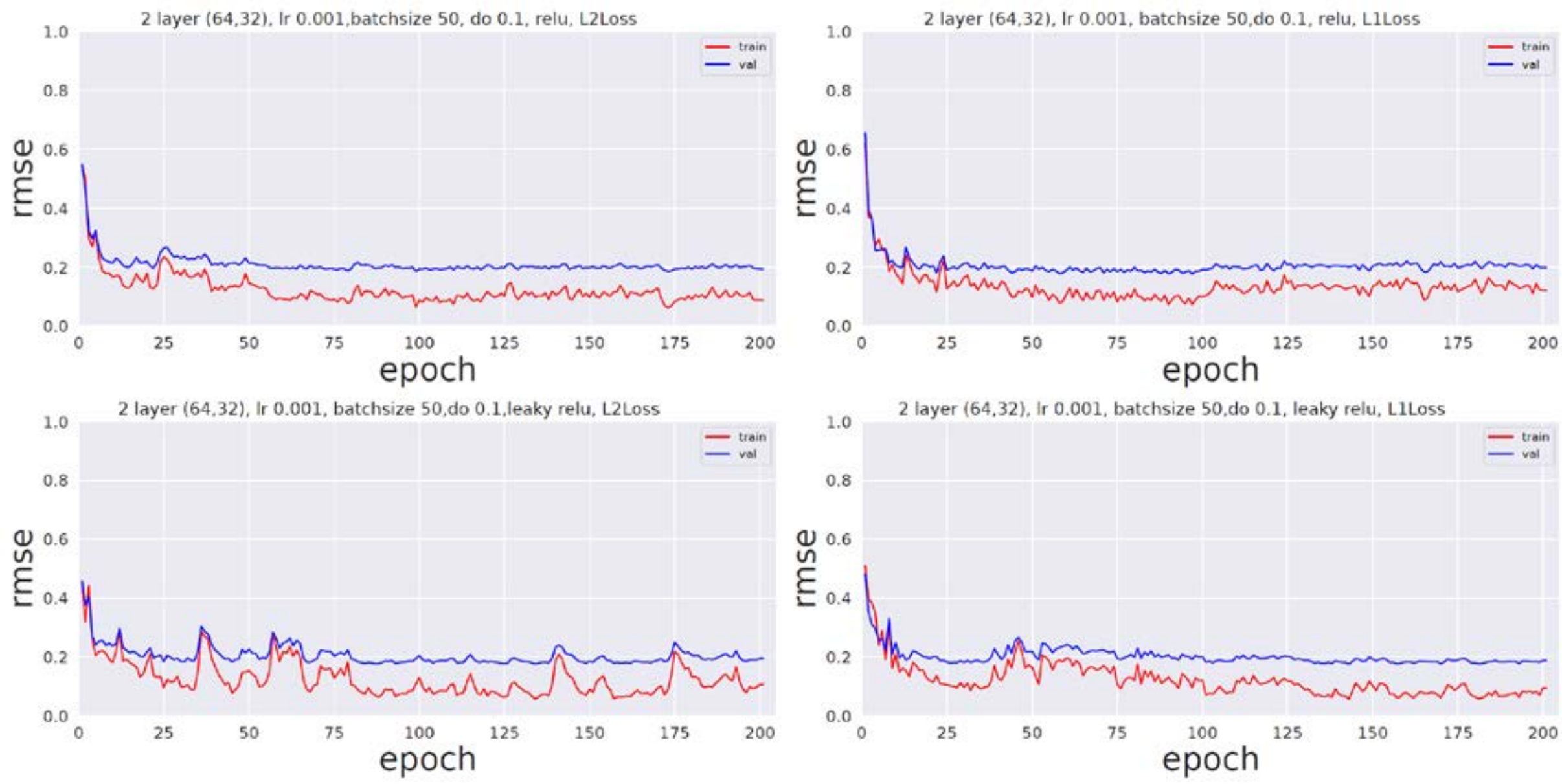

Figure SI1-11. Details on the hyperparameter optimization to find the optimal architecture and setup for the GNN to predict the *S* descriptor. For the GNN including 64 and 32 neurons per layer, respectively, and the identified optimal learning rate of 0.001, different activation and loss functions were tested.

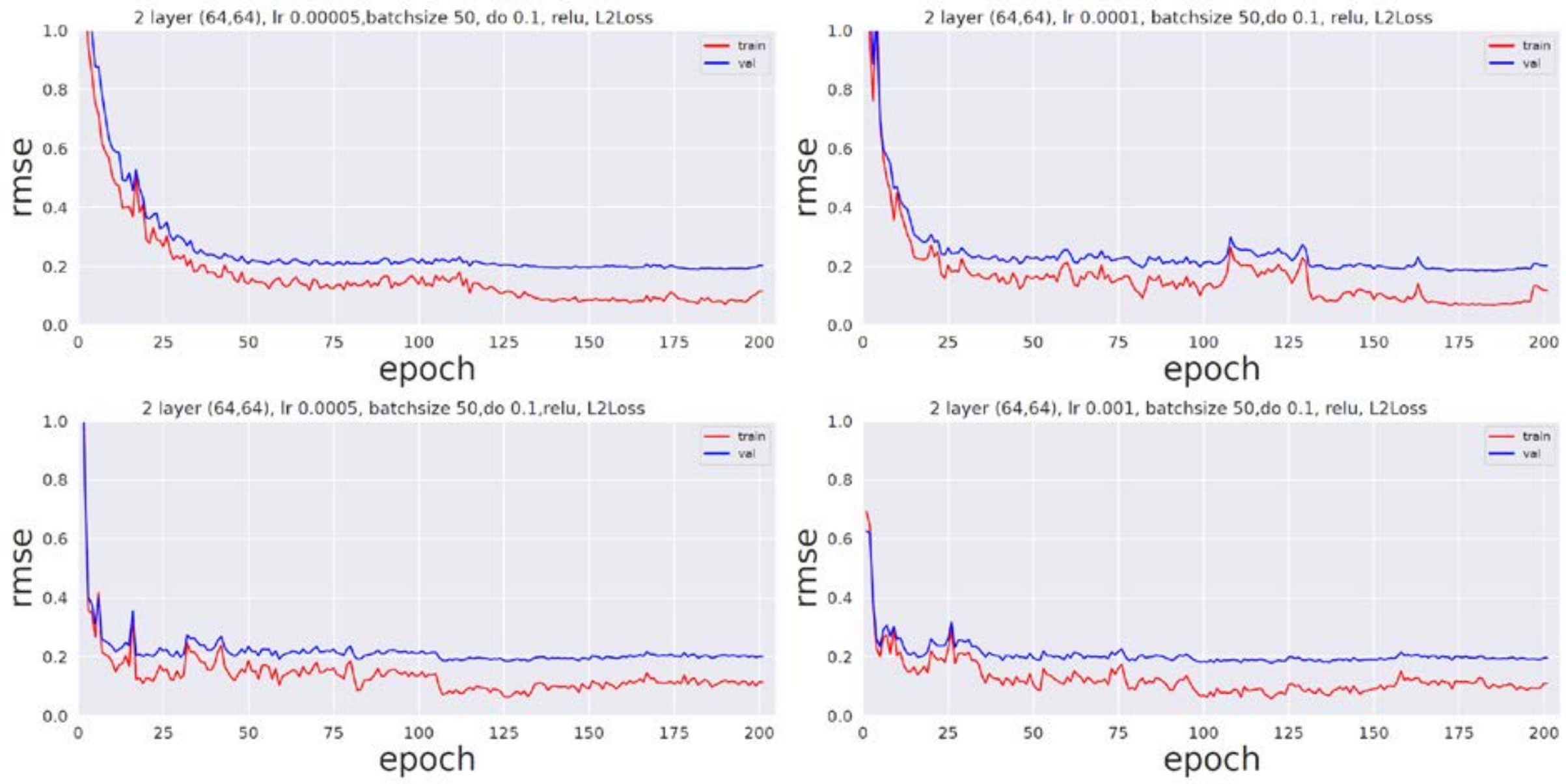

Figure SI1-12. Details on the hyperparameter optimization to find the optimal architecture and setup for the GNN to predict the *S* descriptor. Here, 64 neurons are included per layer and the learning rate was varied. ReLU and L2Loss functions were set to allow a direct comparison of learning rates. Note that we tested more learning rates than depicted here.

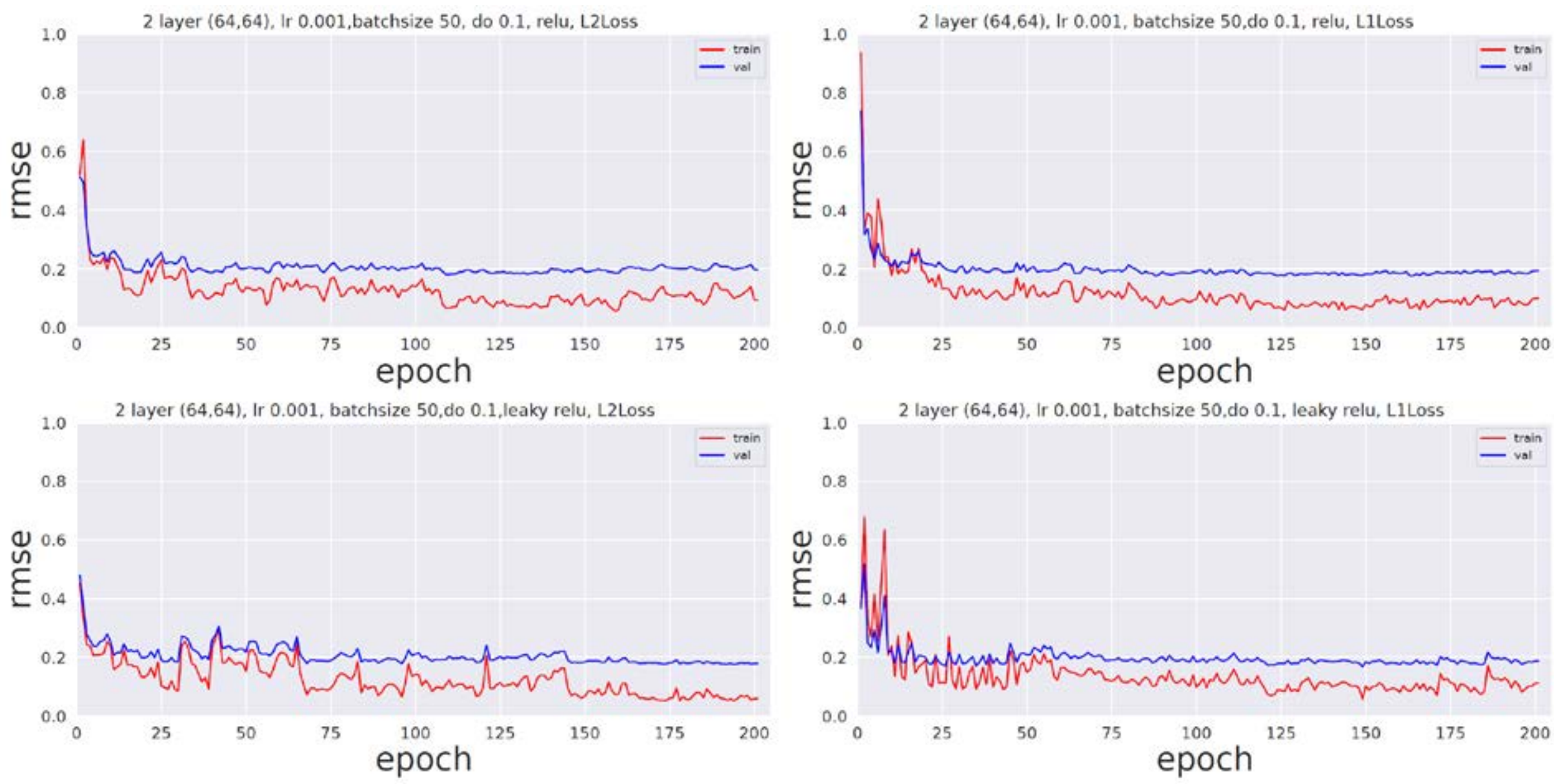

Figure SI1-13. Details on the hyperparameter optimization to find the optimal architecture and setup for the GNN to predict the *S* descriptor. For the GNN including 64 neurons per layer and the identified optimal learning rate of 0.001 different activation and loss functions were tested.

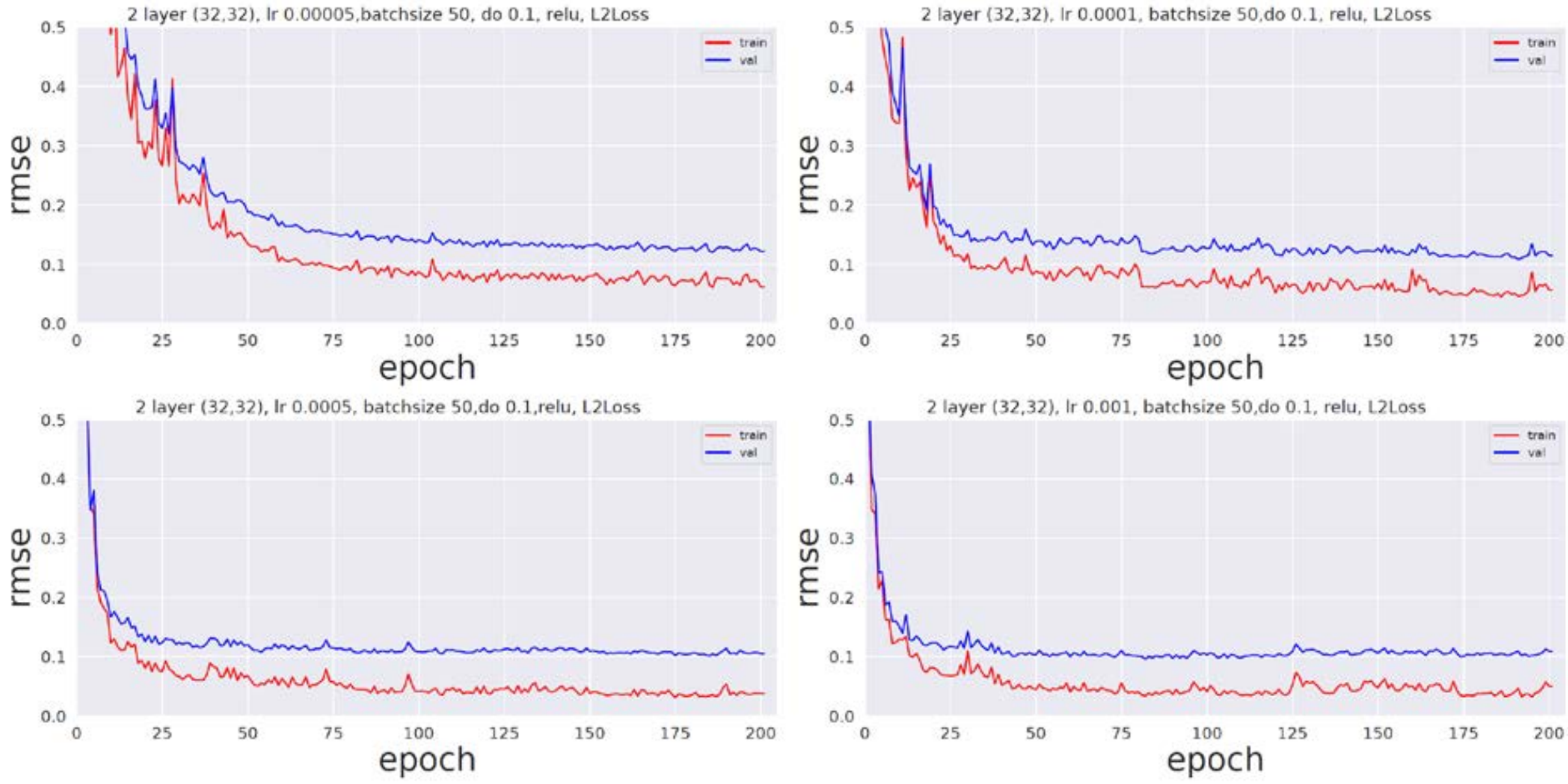

Figure SI1-14. Details on the hyperparameter optimization to find the optimal architecture and setup for the GNN to predict the *B* descriptor. Here, 32 neurons are included per layer and the learning rate was varied. ReLU and L2Loss functions were set to allow a direct comparison of learning rates. Note that we tested more learning rates than depicted here.

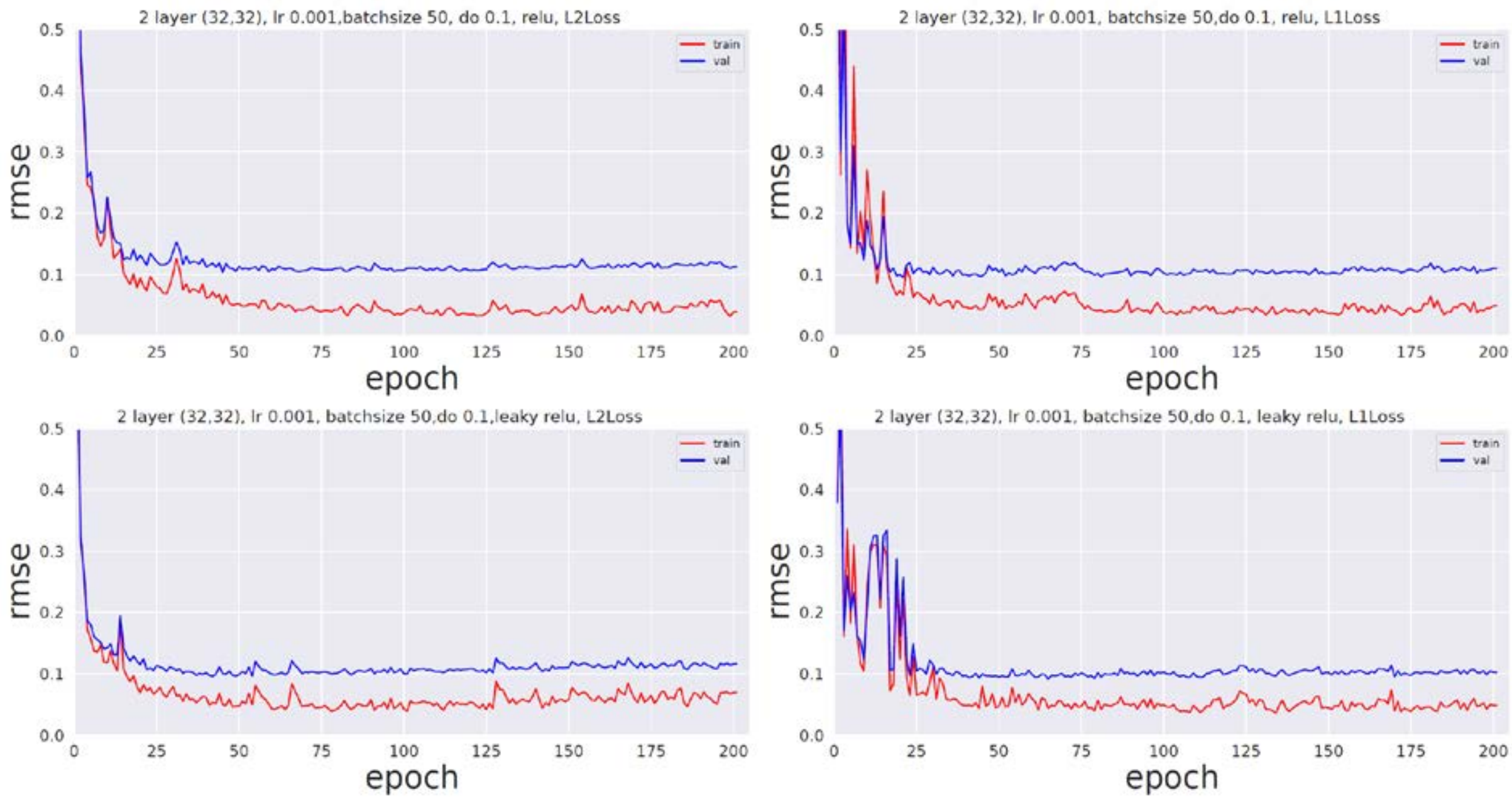

Figure SI1-15. Details on the hyperparameter optimization to find the optimal architecture and setup for the GNN to predict the *B* descriptor. For the GNN including 32 neurons per layer and the identified optimal learning rate of 0.001 different activation and loss functions were tested.

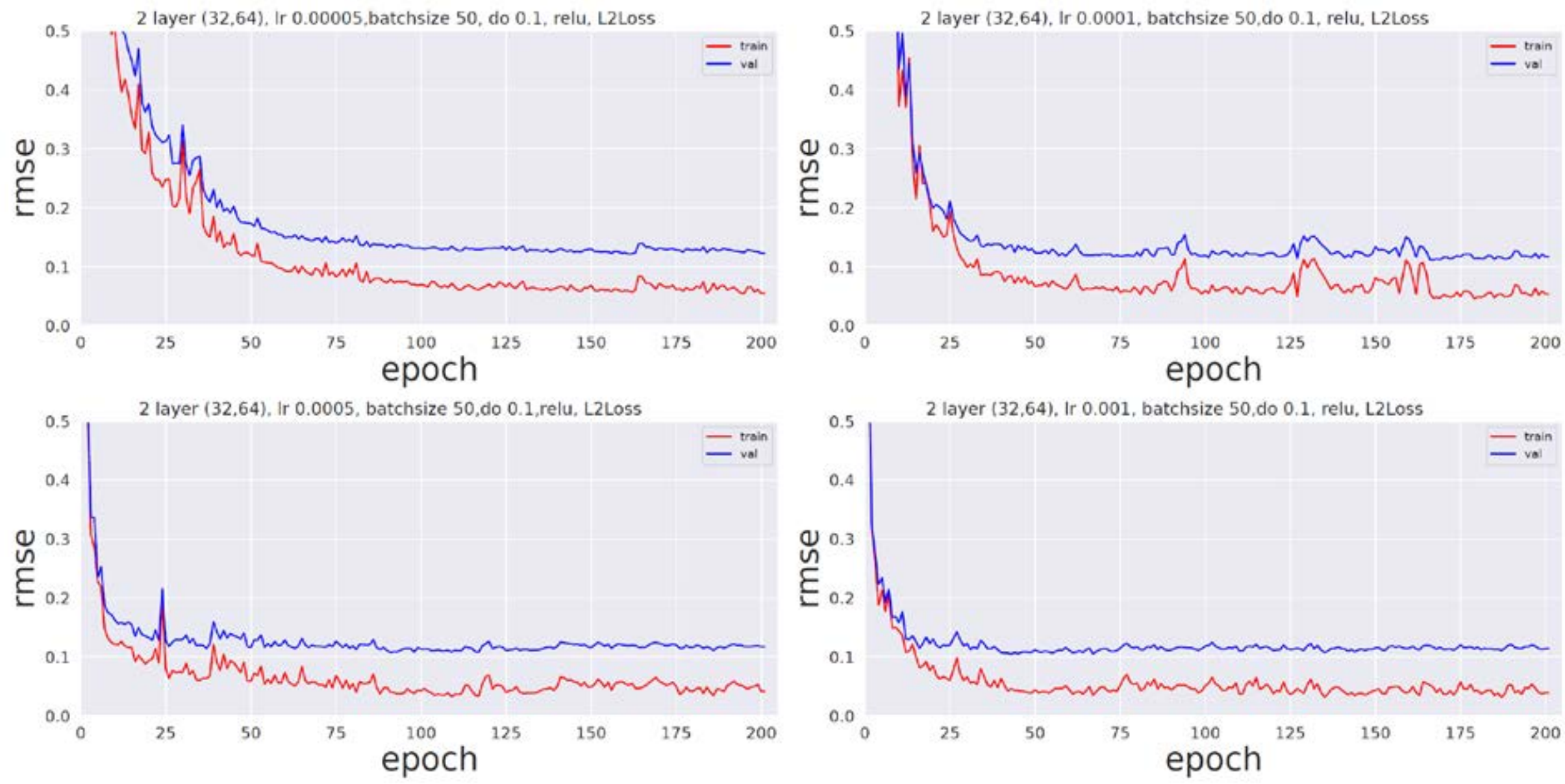

Figure SI1-16. Details on the hyperparameter optimization to find the optimal architecture and setup for the GNN to predict the *B* descriptor. Here, 32 and 64 neurons are included in the two hidden layers and the learning rate was varied. ReLU and L2Loss functions were set to allow a direct comparison of learning rates. Note that we tested more learning rates than depicted here.

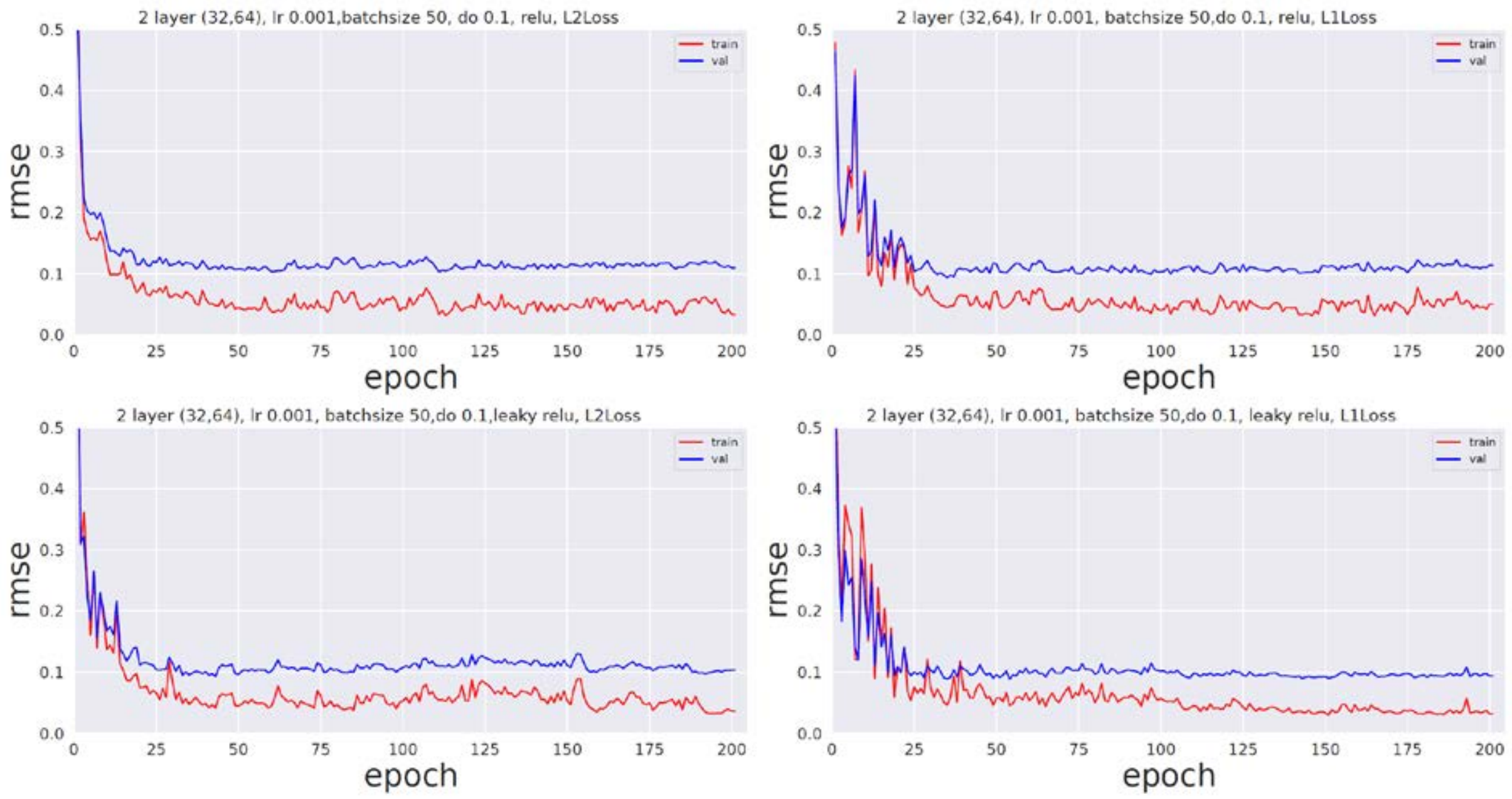

Figure SI1-17. Details on the hyperparameter optimization to find the optimal architecture and setup for the GNN to predict the *B* descriptor. For the GNN including 32 and 64 neurons per layer, respectively, and the identified optimal learning rate of 0.001, different activation and loss functions were tested.

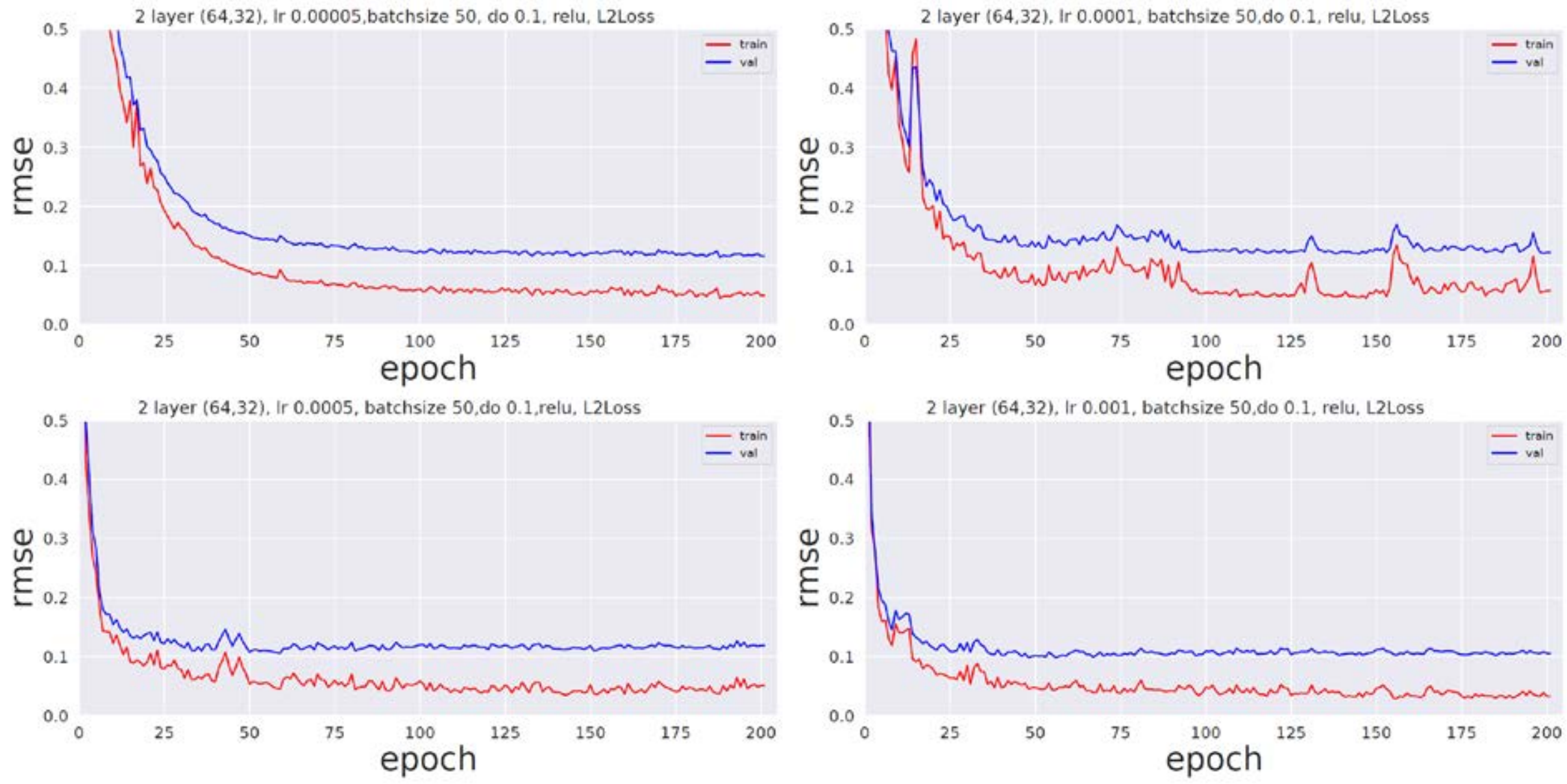

Figure SI1-18. Details on the hyperparameter optimization to find the optimal architecture and setup for the GNN to predict the *B* descriptor. Here, 64 and 32 neurons are included in the two hidden layers and the learning rate was varied. ReLU and L2Loss functions were set to allow a direct comparison of learning rates. Note that we tested more learning rates than depicted here.

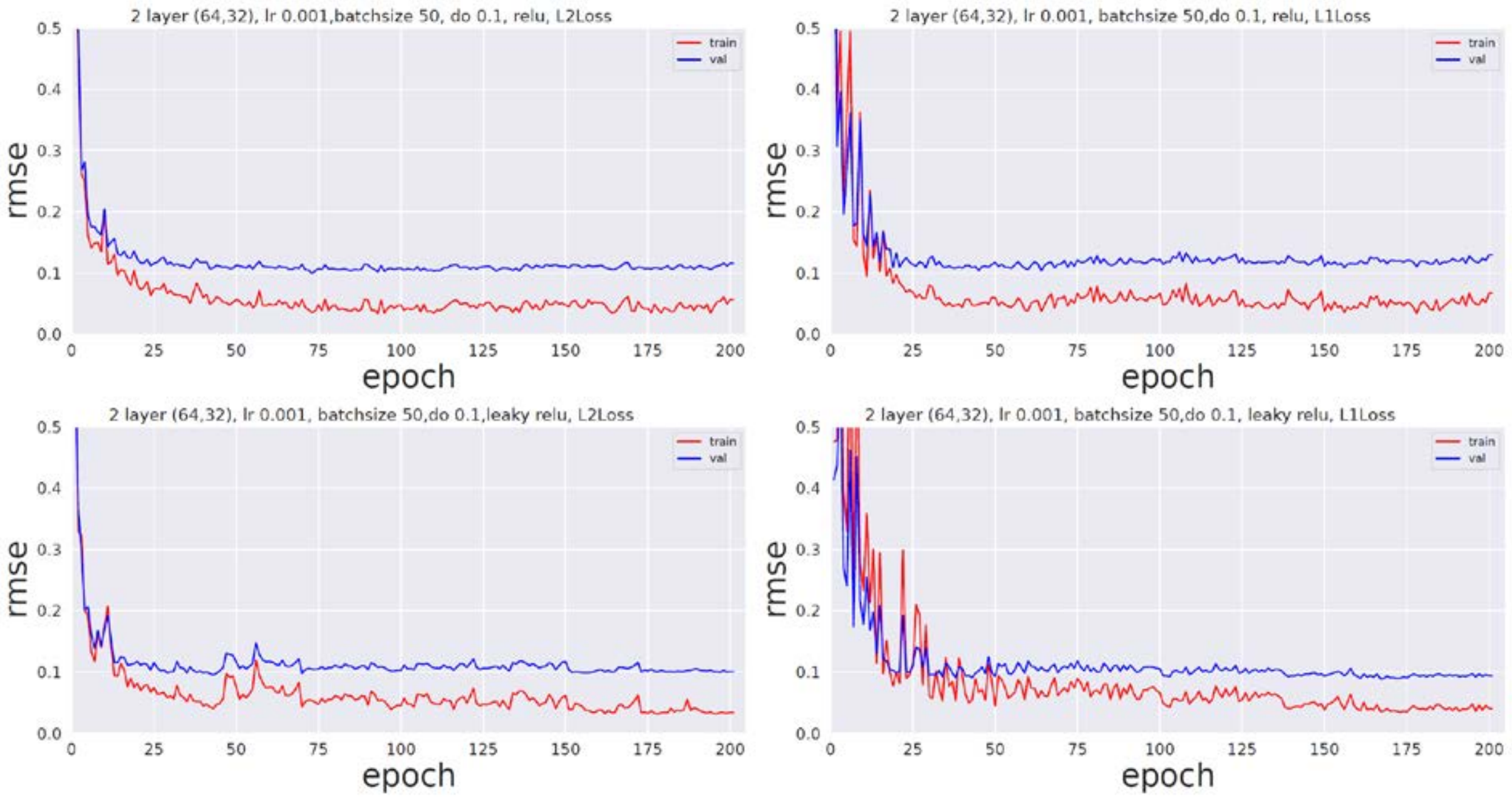

Figure SI1-19. Details on the hyperparameter optimization to find the optimal architecture and setup for the GNN to predict the *B* descriptor. For the GNN including 64 and 32 neurons per layer, respectively, and the identified optimal learning rate of 0.001, different activation and loss functions were tested.

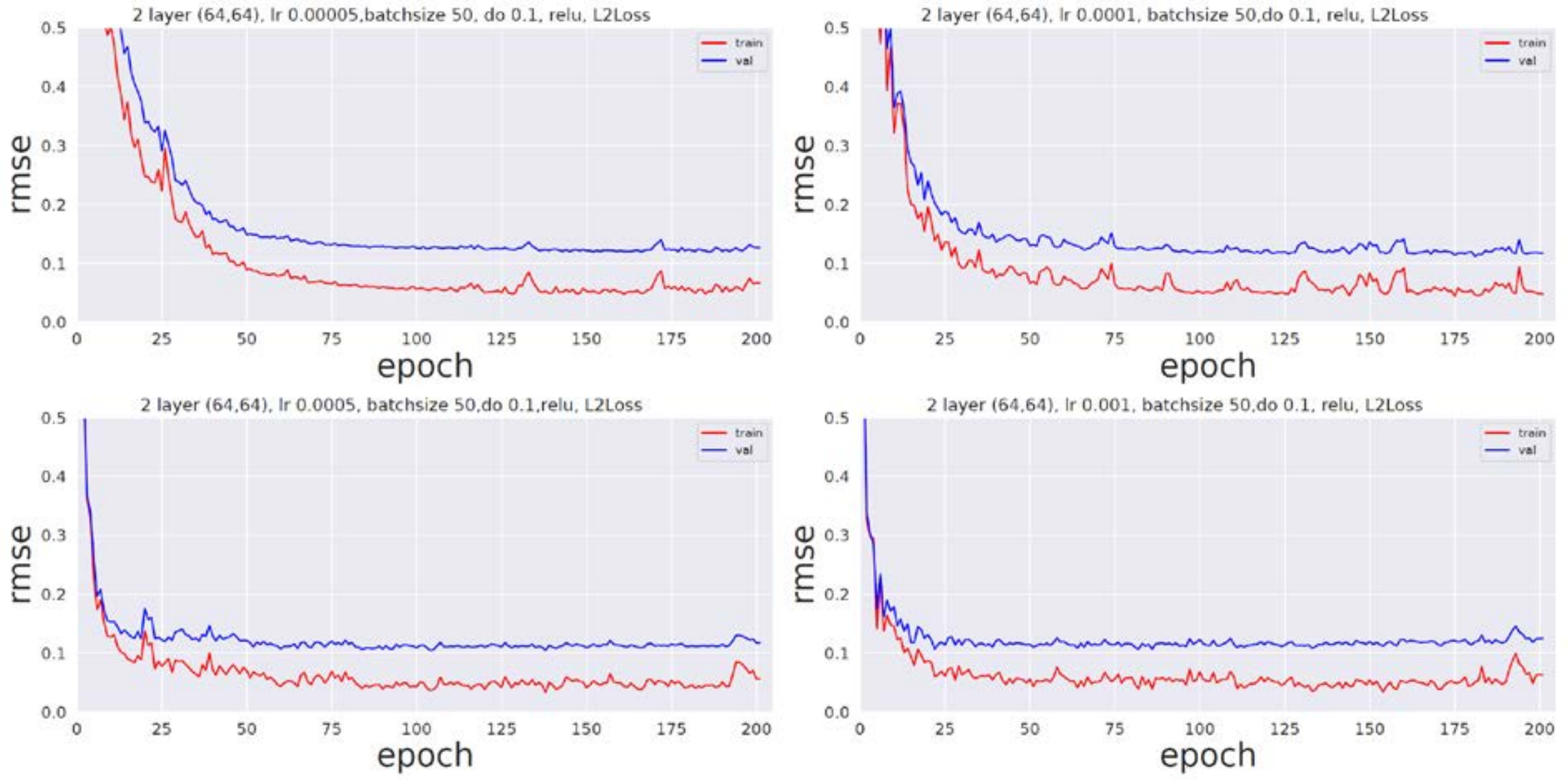

Figure SI1-20. Details on the hyperparameter optimization to find the optimal architecture and setup for the GNN to predict the *B* descriptor. Here, 64 neurons are included per layer and the learning rate was varied. ReLU and L2Loss functions were set to allow a direct comparison of learning rates. Note that we tested more learning rates than depicted here.

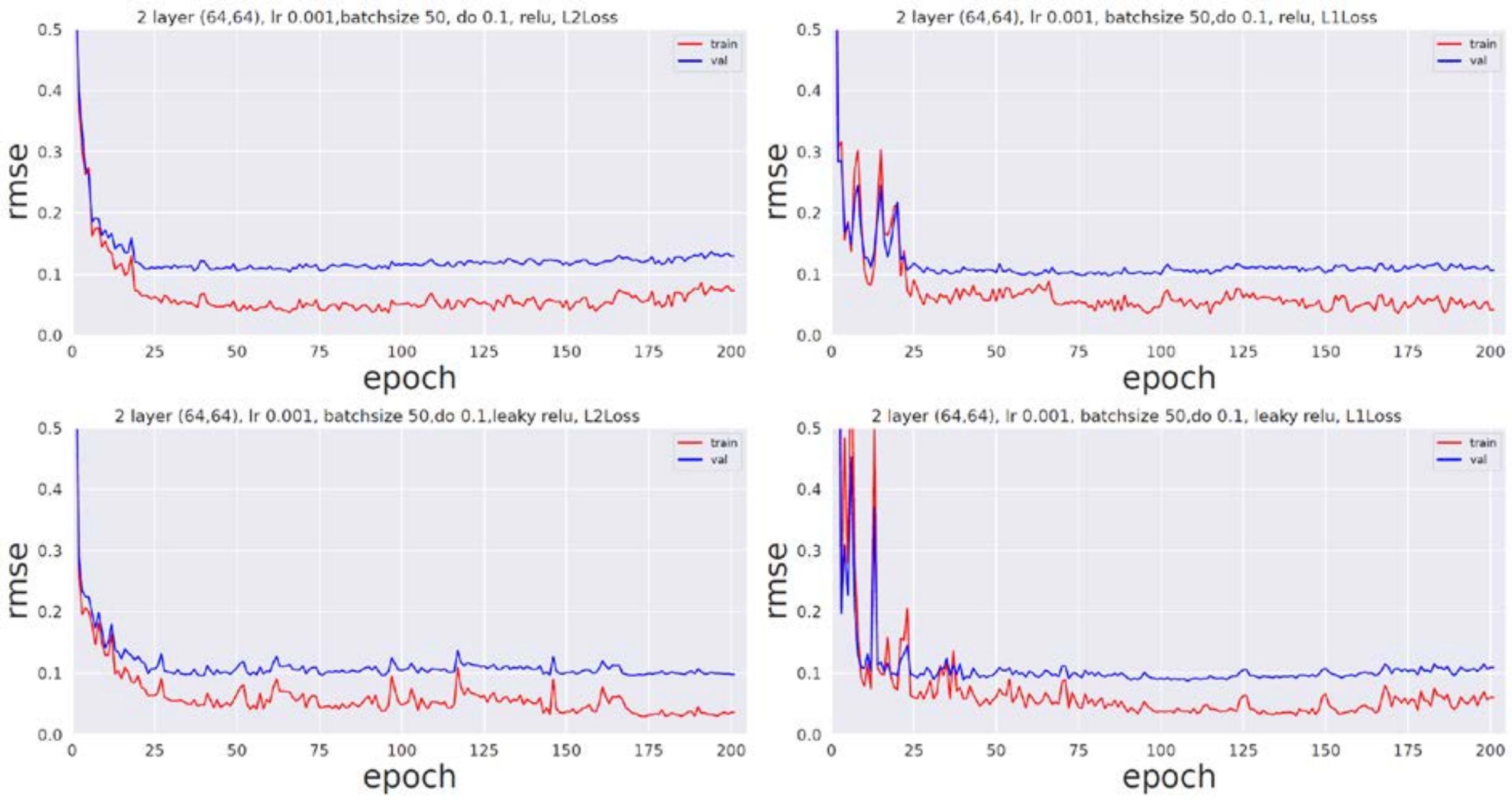

Figure SI1-21. Details on the hyperparameter optimization to find the optimal architecture and setup for the GNN to predict the *B* descriptor. For the GNN including 64 neurons per layer and the identified optimal learning rate of 0.001 different activation and loss functions were tested.

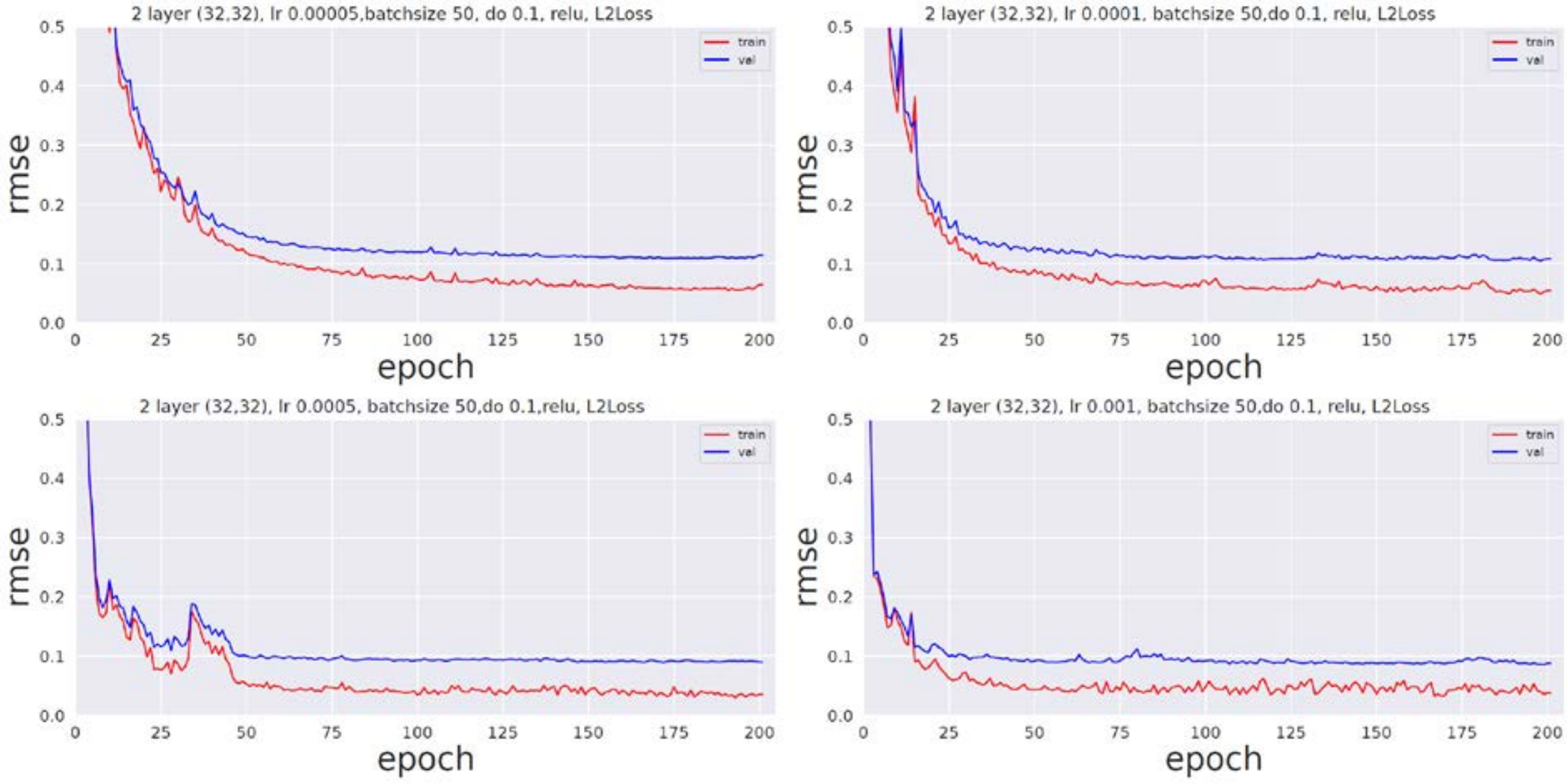

Figure SI1-22. Details on the hyperparameter optimization to find the optimal architecture and setup for the GNN to predict the *A* descriptor. Here, 32 neurons are included per layer and the learning rate was varied. ReLU and L2Loss functions were set to allow a direct comparison of learning rates. Note that we tested more learning rates than depicted here.

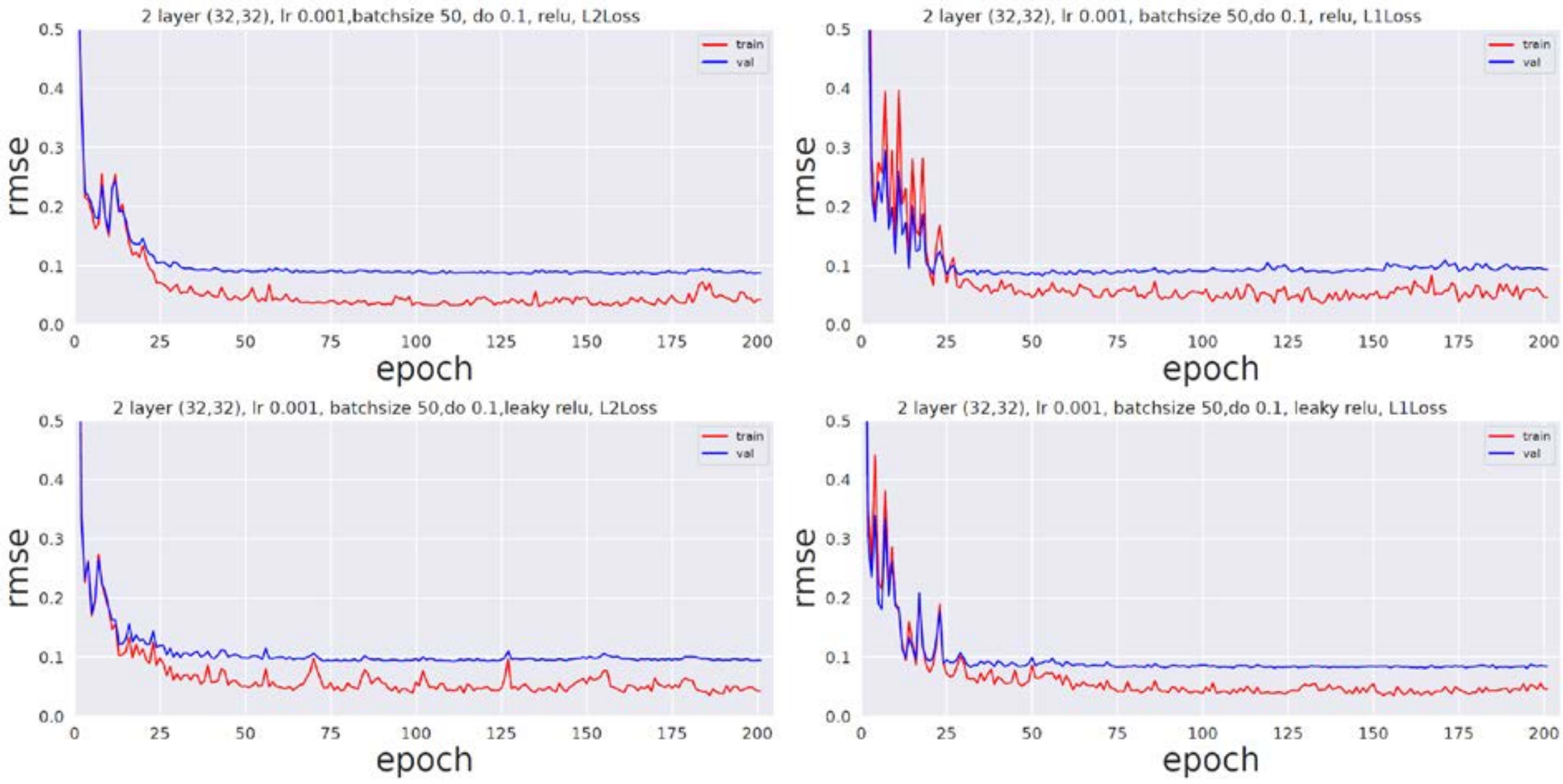

Figure SI1-23. Details on the hyperparameter optimization to find the optimal architecture and setup for the GNN to predict the *A* descriptor. For the GNN including 32 neurons per layer and the identified optimal learning rate of 0.001 different activation and loss functions were tested.

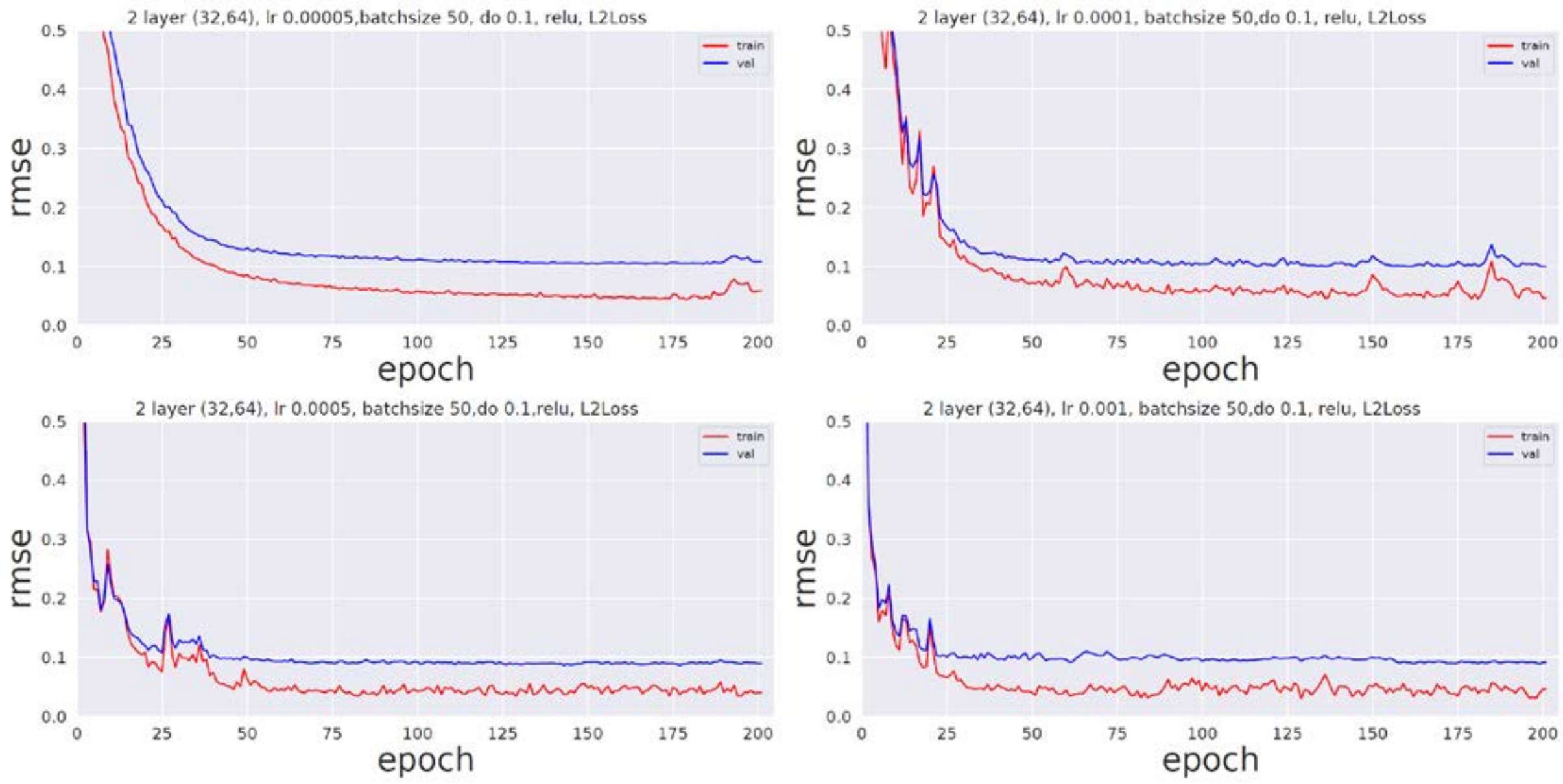

Figure SI1-24. Details on the hyperparameter optimization to find the optimal architecture and setup for the GNN to predict the *A* descriptor. Here, 32 and 64 neurons are included in the two hidden layers and the learning rate was varied. ReLU and L2Loss functions were set to allow a direct comparison of learning rates. Note that we tested more learning rates than depicted here.

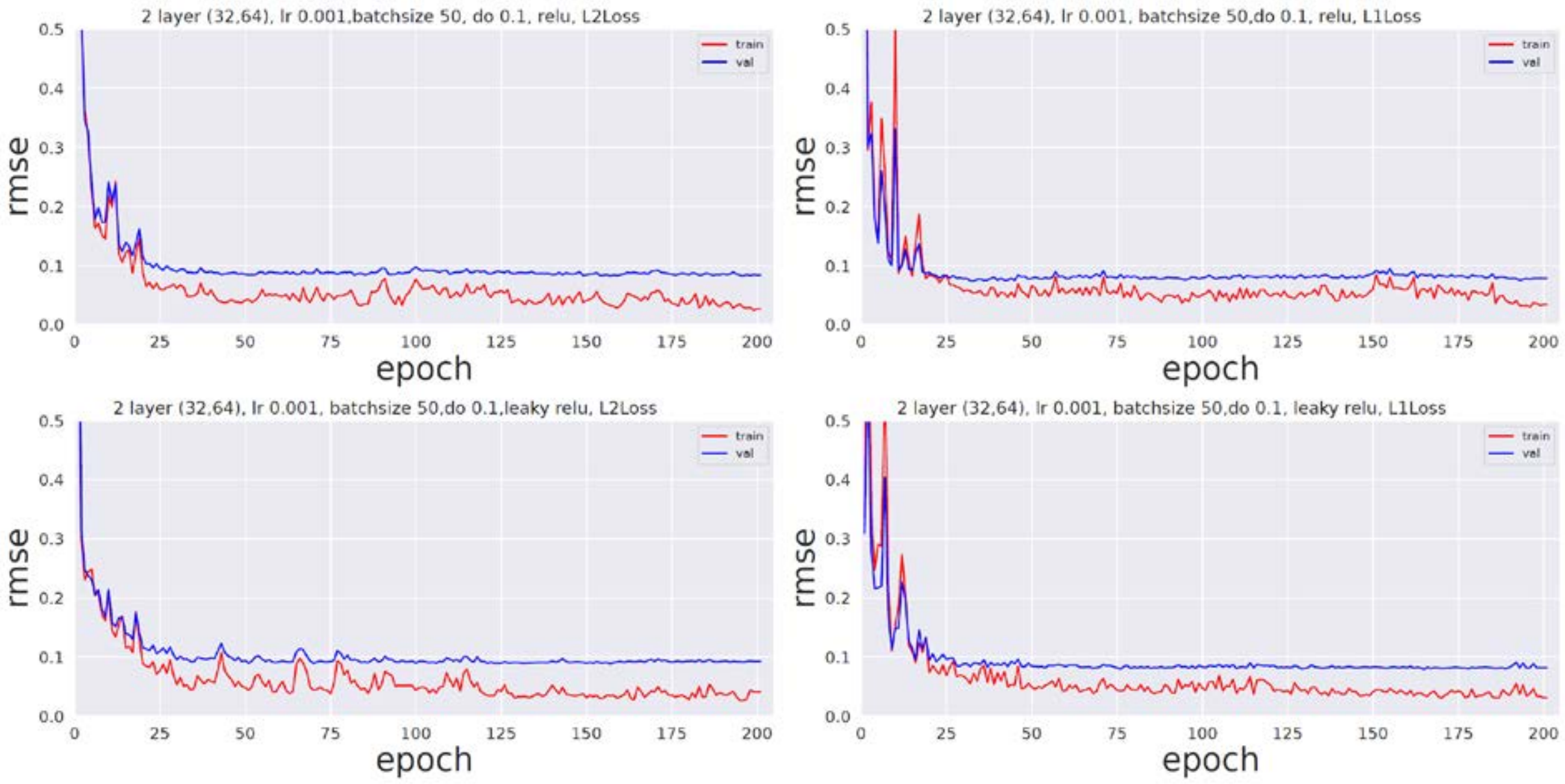

Figure SI1-25. Details on the hyperparameter optimization to find the optimal architecture and setup for the GNN to predict the *A* descriptor. For the GNN including 32 and 64 neurons per layer, respectively, and the identified optimal learning rate of 0.001, different activation and loss functions were tested.

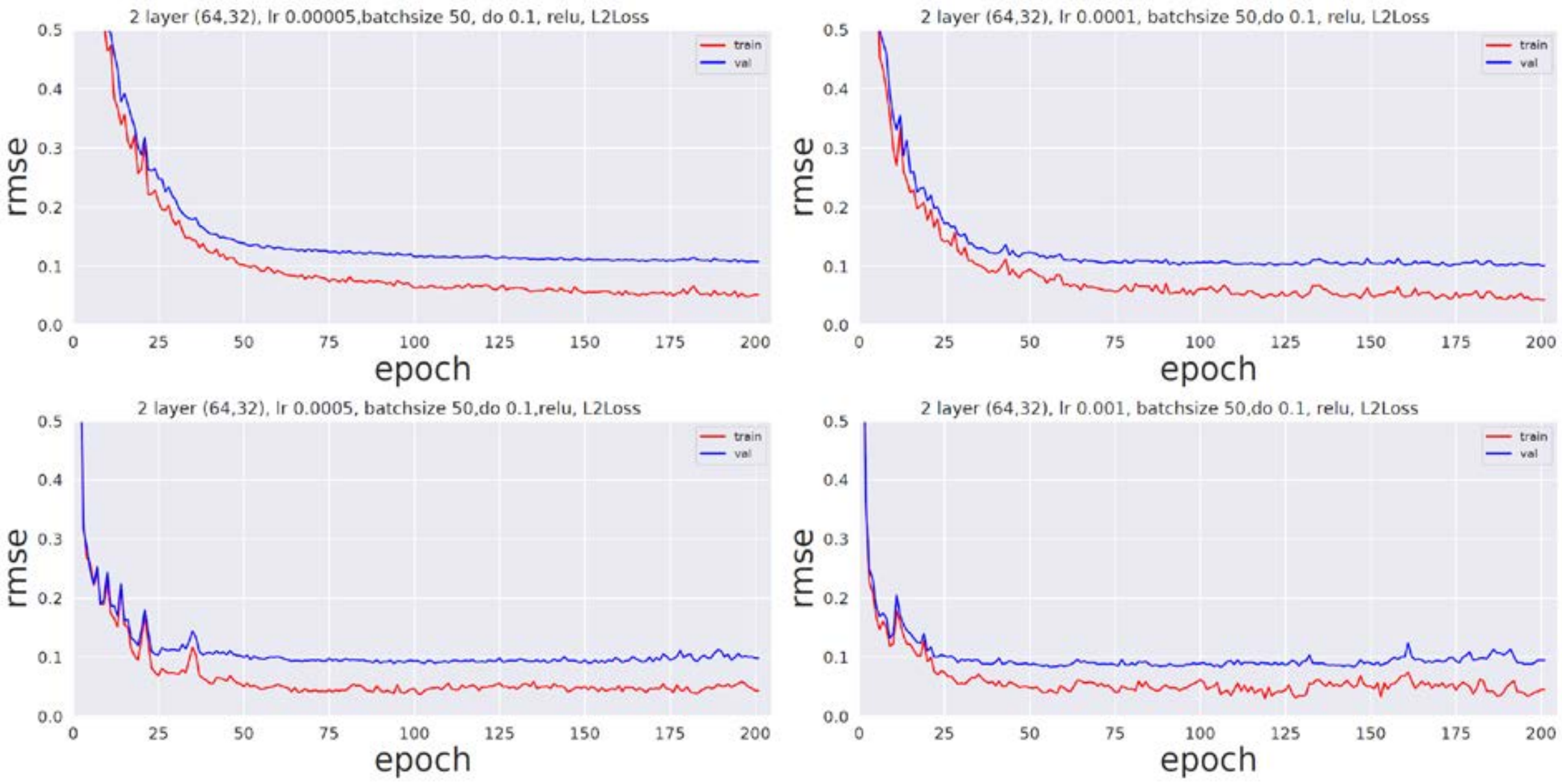

Figure SI1-26. Details on the hyperparameter optimization to find the optimal architecture and setup for the GNN to predict the *A* descriptor. Here, 64 and 32 neurons are included in the two hidden layers and the learning rate was varied. ReLU and L2Loss functions were set to allow a direct comparison of learning rates. Note that we tested more learning rates than depicted here.

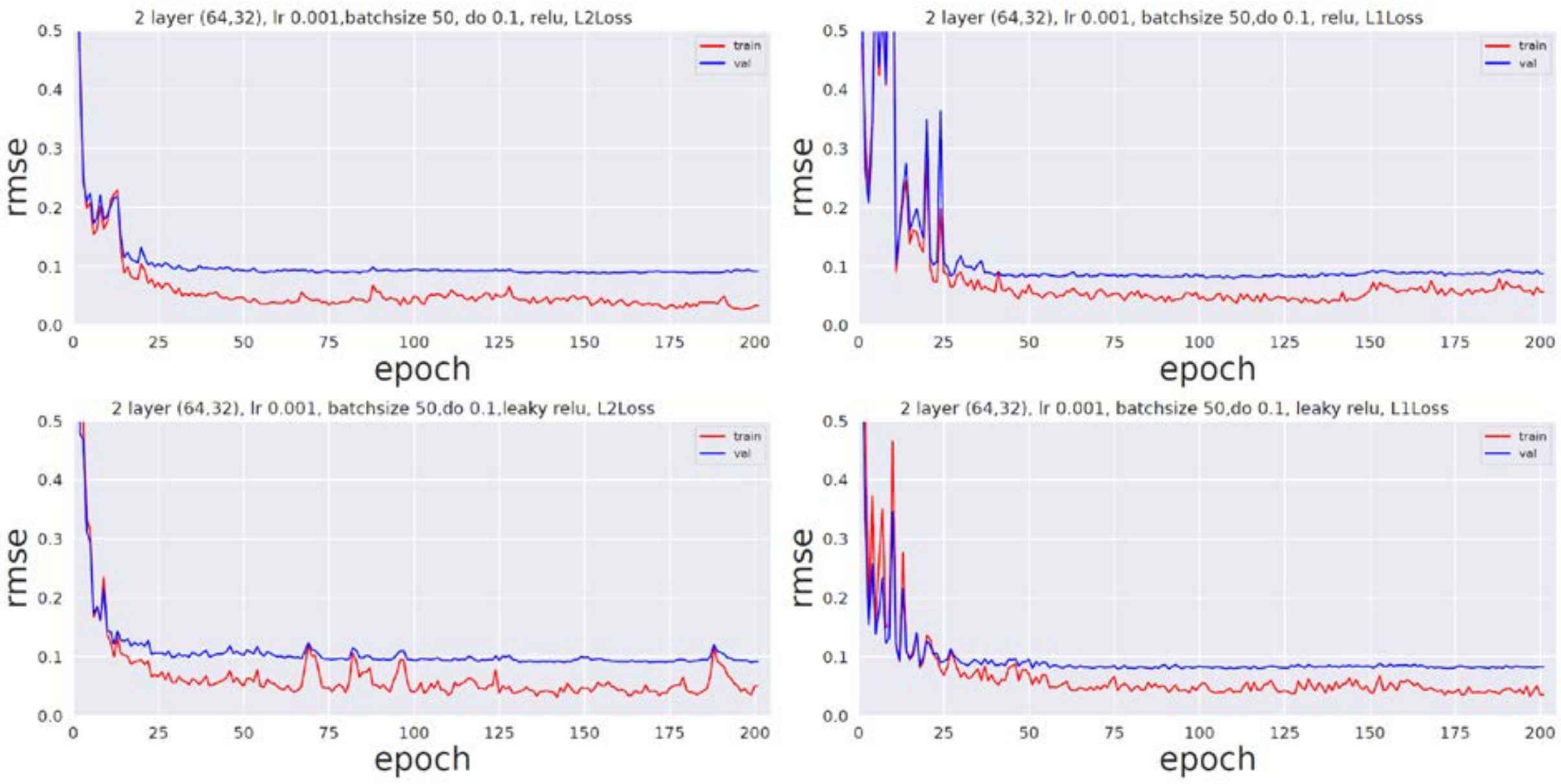

Figure SI1-27. Details on the hyperparameter optimization to find the optimal architecture and setup for the GNN to predict the *A* descriptor. For the GNN including 64 and 32 neurons per layer, respectively, and the identified optimal learning rate of 0.001, different activation and loss functions were tested.

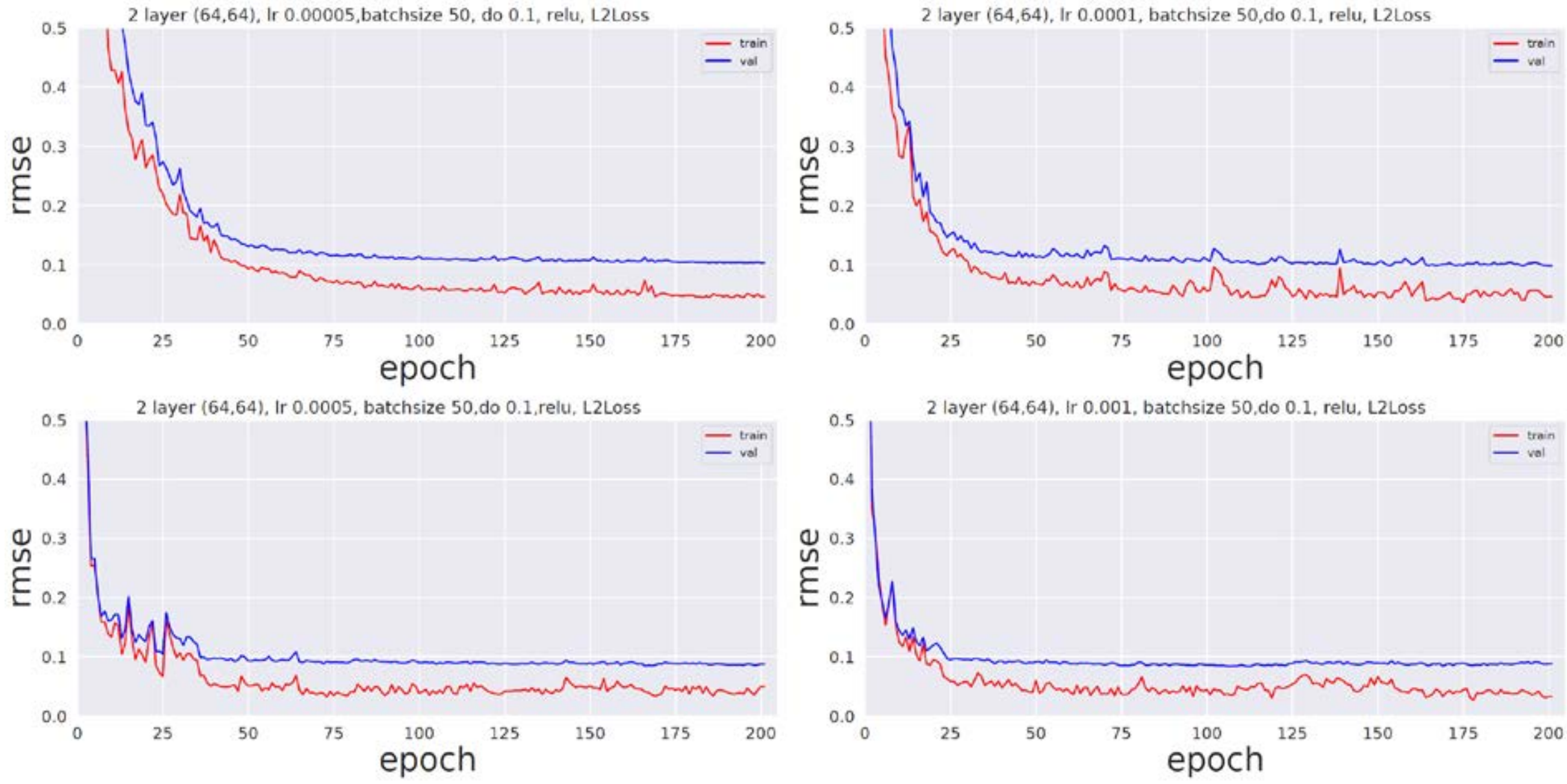

Figure SI1-28. Details on the hyperparameter optimization to find the optimal architecture and setup for the GNN to predict the *A* descriptor. Here, 64 neurons are included per layer and the learning rate was varied. ReLU and L2Loss functions were set to allow a direct comparison of learning rates. Note that we tested more learning rates than depicted here.

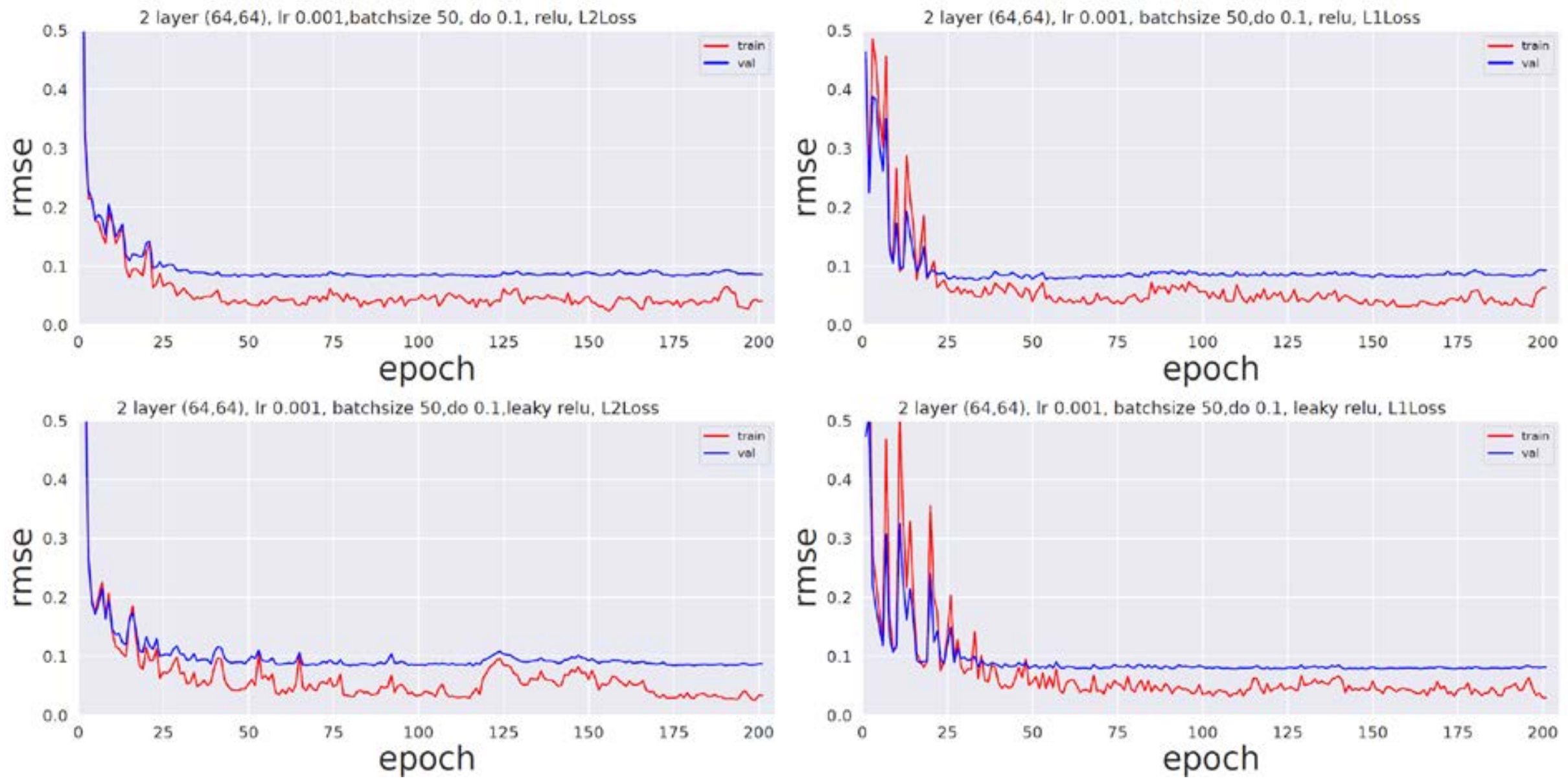

Figure SI1-29. Details on the hyperparameter optimization to find the optimal architecture and setup for the GNN to predict the *A* descriptor. For the GNN including 64 neurons per layer and the identified optimal learning rate of 0.001 different activation and loss functions were tested.

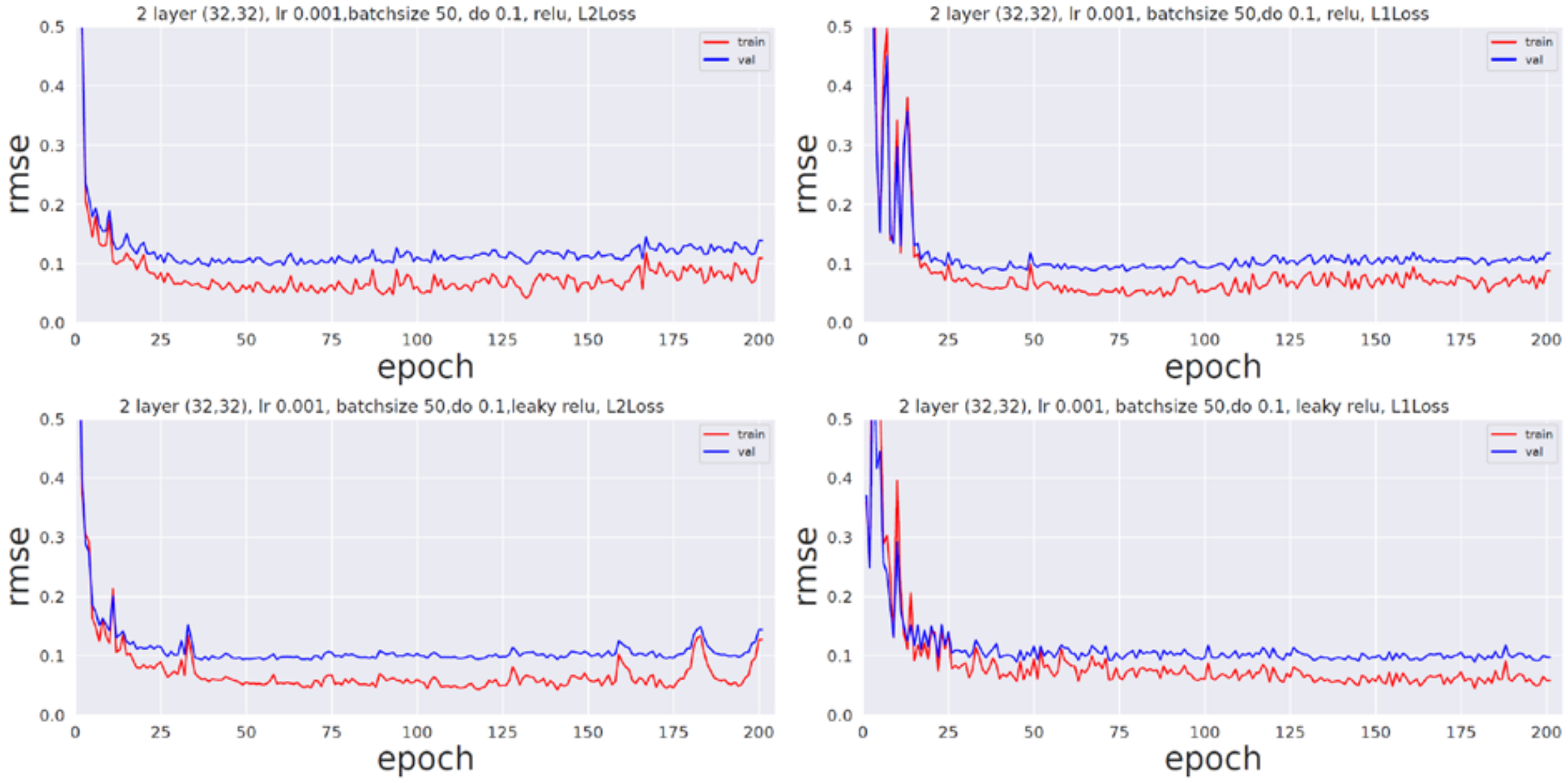

Figure SI1-30. Details on the hyperparameter optimization to find the optimal architecture and setup for the GNN to predict the *E* descriptor. For the GNN including 32 neurons per layer and the identified optimal learning rate of 0.001 different activation and loss functions were tested.

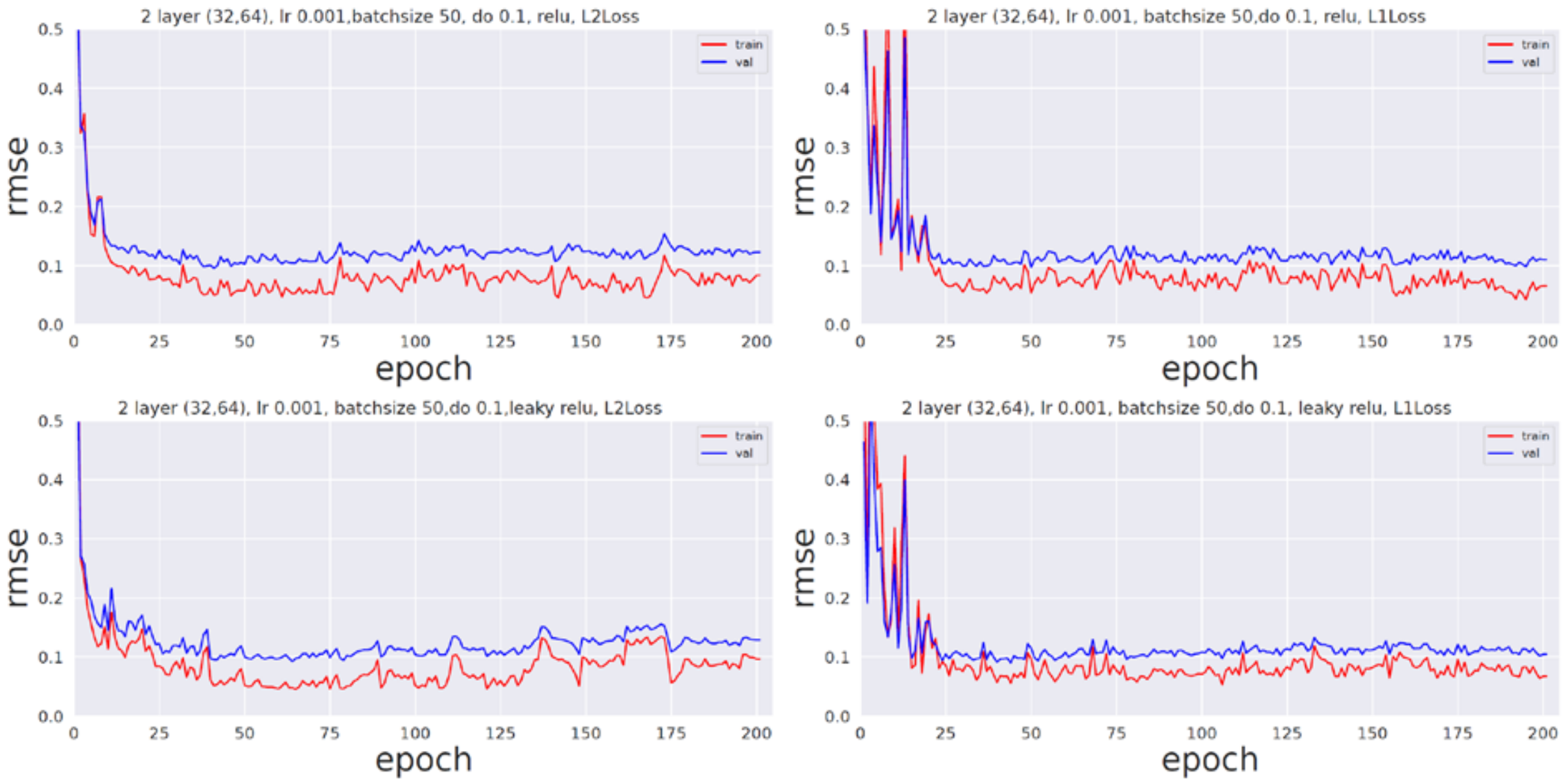

Figure SI1-31. Details on the hyperparameter optimization to find the optimal architecture and setup for the GNN to predict the *E* descriptor. For the GNN including 32 and 64 neurons per layer, respectively, and the identified optimal learning rate of 0.001, different activation and loss functions were tested.

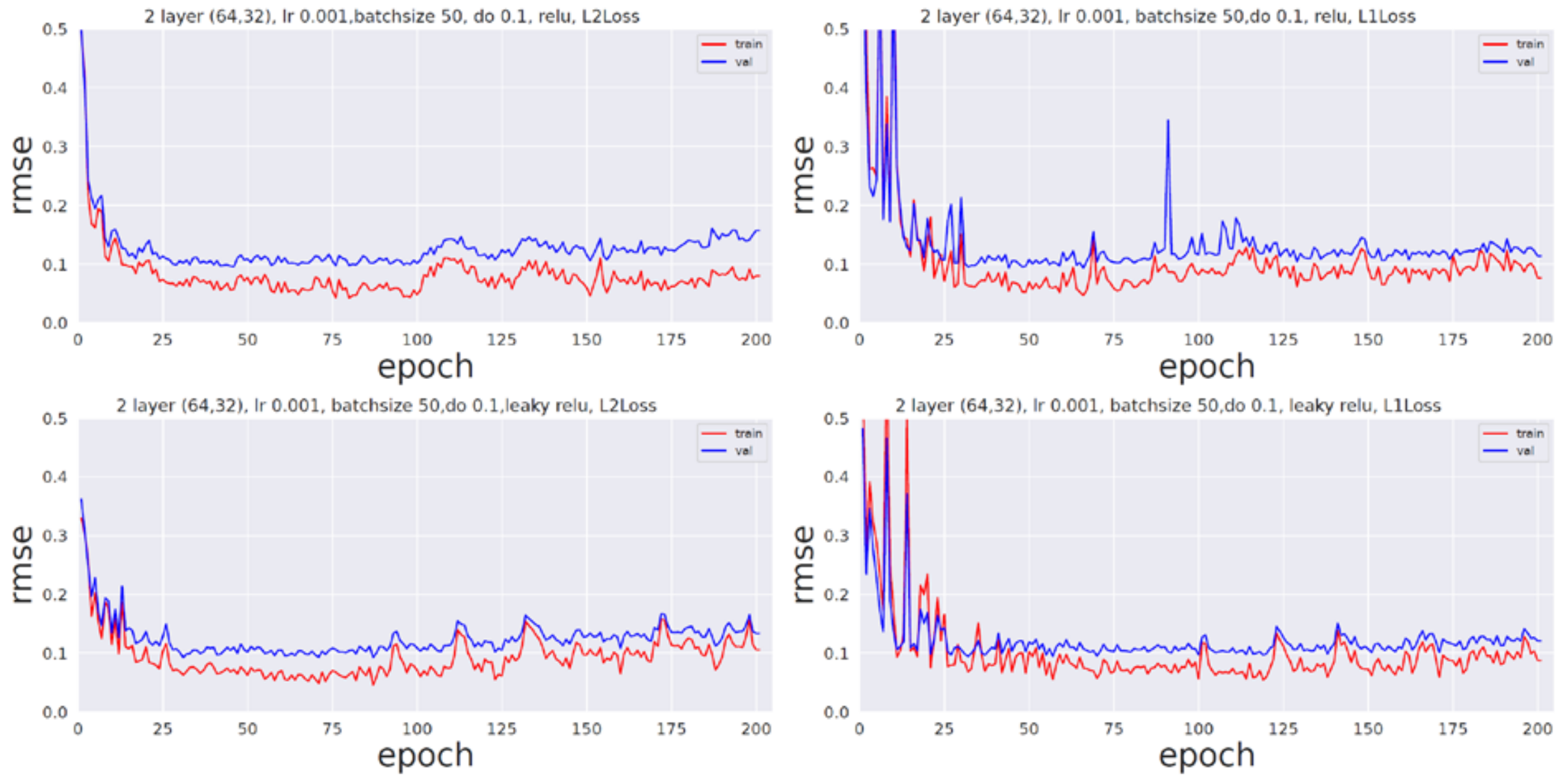

Figure SI1-32. Details on the hyperparameter optimization to find the optimal architecture and setup for the GNN to predict the *E* descriptor. For the GNN including 64 and 32 neurons per layer, respectively, and the identified optimal learning rate of 0.001, different activation and loss functions were tested.

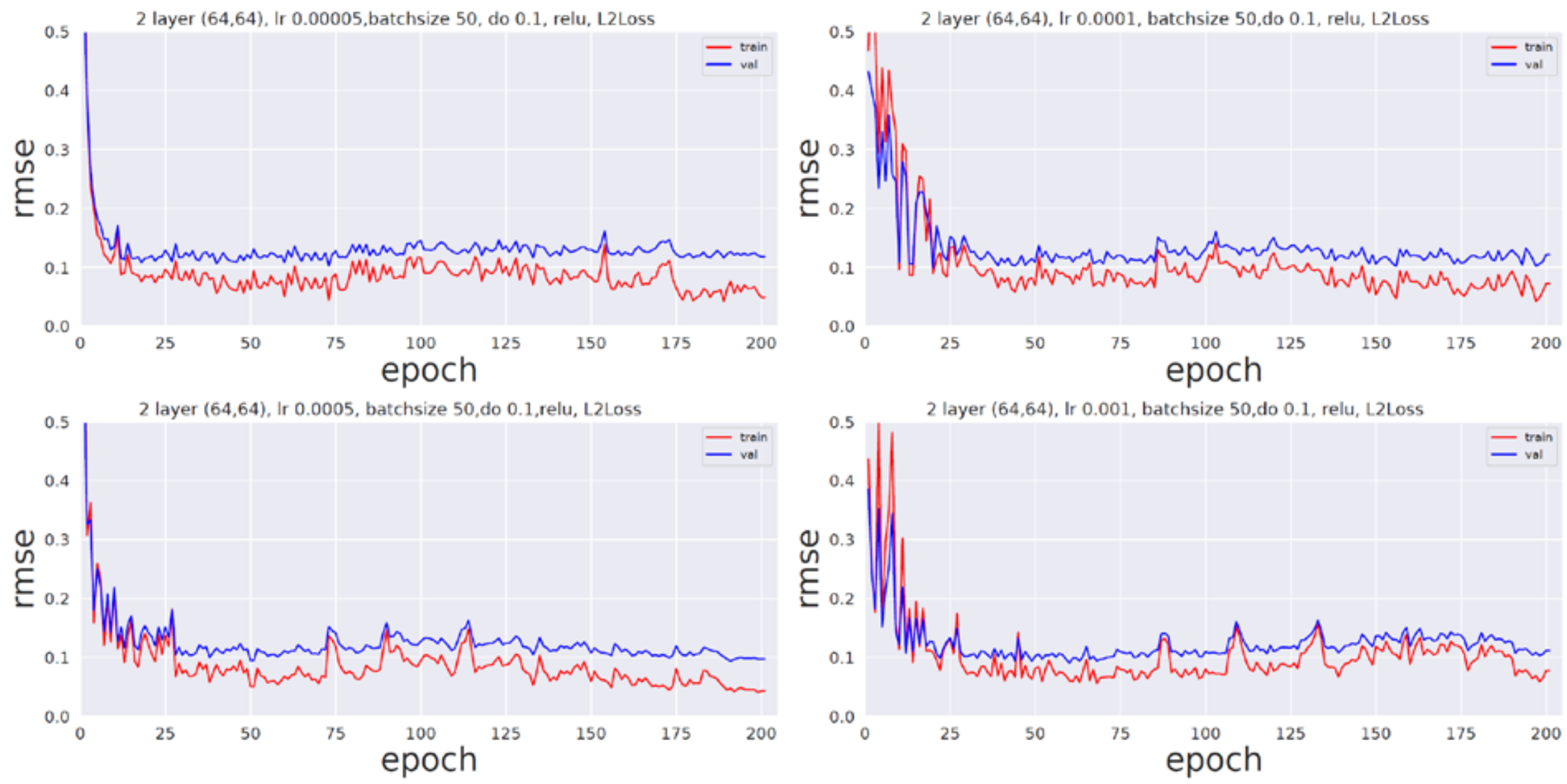

Figure SI1-33. Details on the hyperparameter optimization to find the optimal architecture and setup for the GNN to predict the *E* descriptor. For the GNN including 64 neurons per layer and the identified optimal learning rate of 0.001 different activation and loss functions were tested.

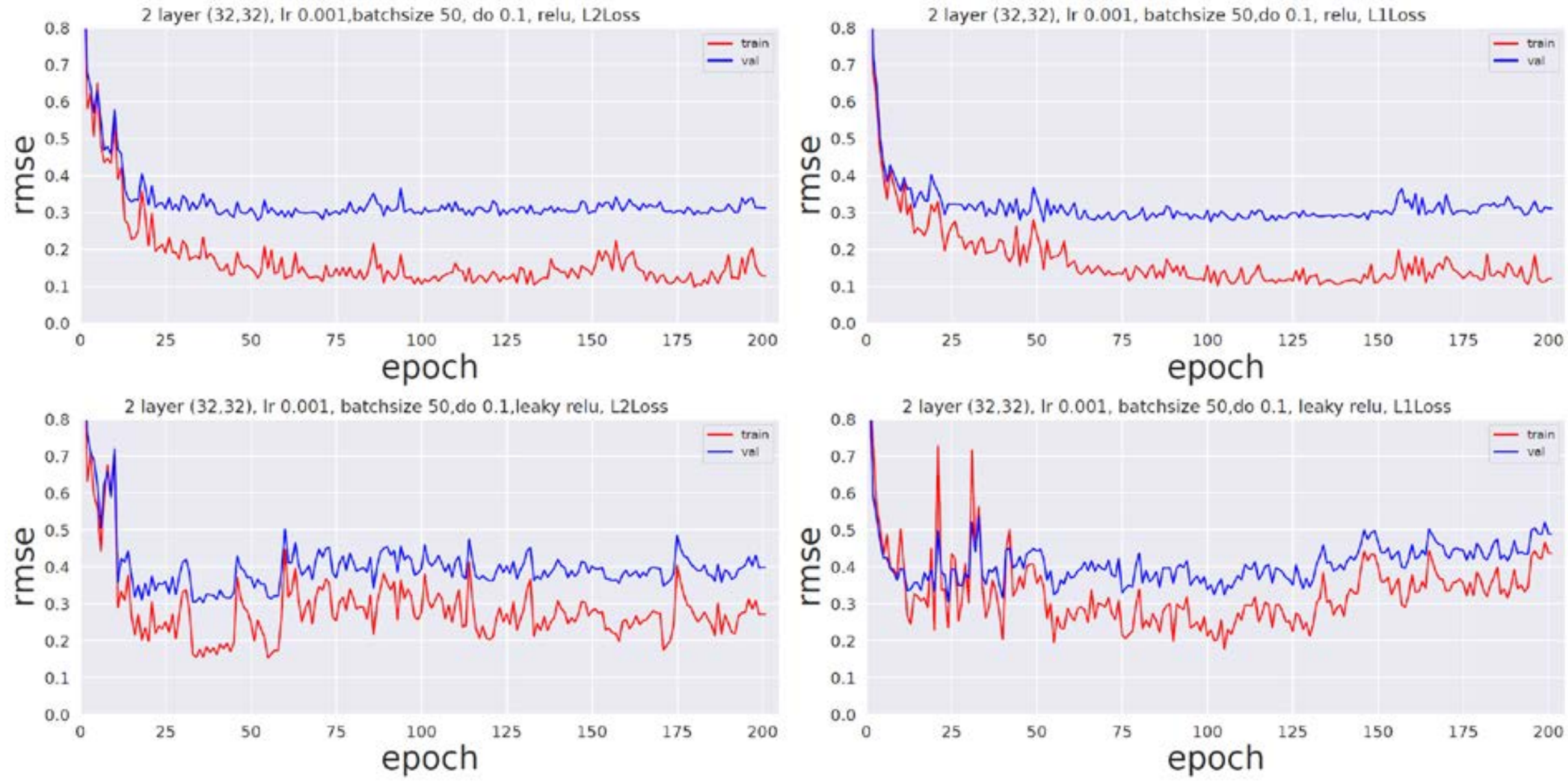

Figure SI1-34. Details on the hyperparameter optimization to find the optimal architecture and setup for the GNN to predict the *L* descriptor. For the GNN including 32 neurons per layer and the identified optimal learning rate of 0.001 different activation and loss functions were tested.

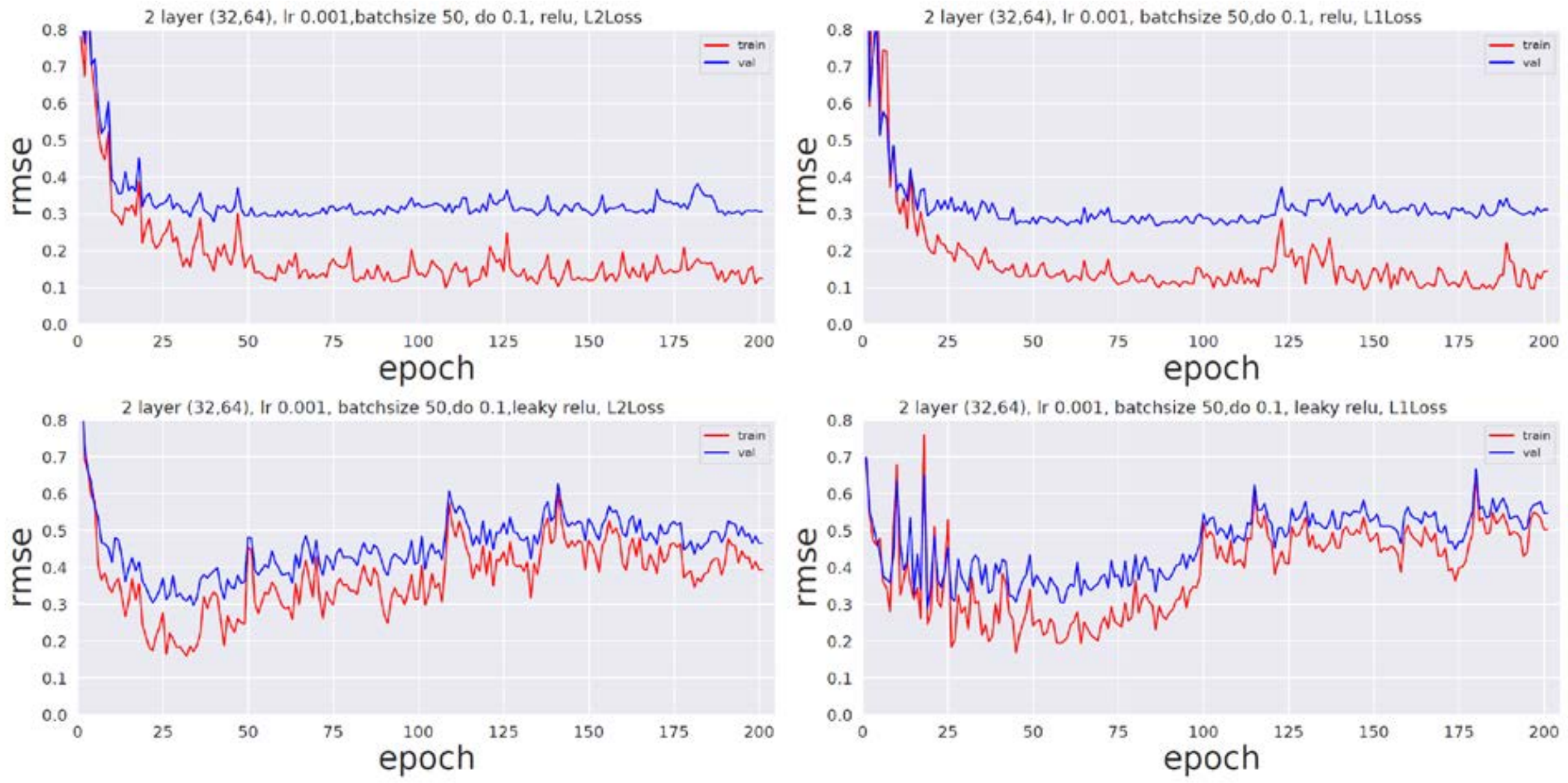

Figure SI1-35. Details on the hyperparameter optimization to find the optimal architecture and setup for the GNN to predict the *L* descriptor. For the GNN including 32 and 64 neurons per layer, respectively, and the identified optimal learning rate of 0.001, different activation and loss functions were tested.

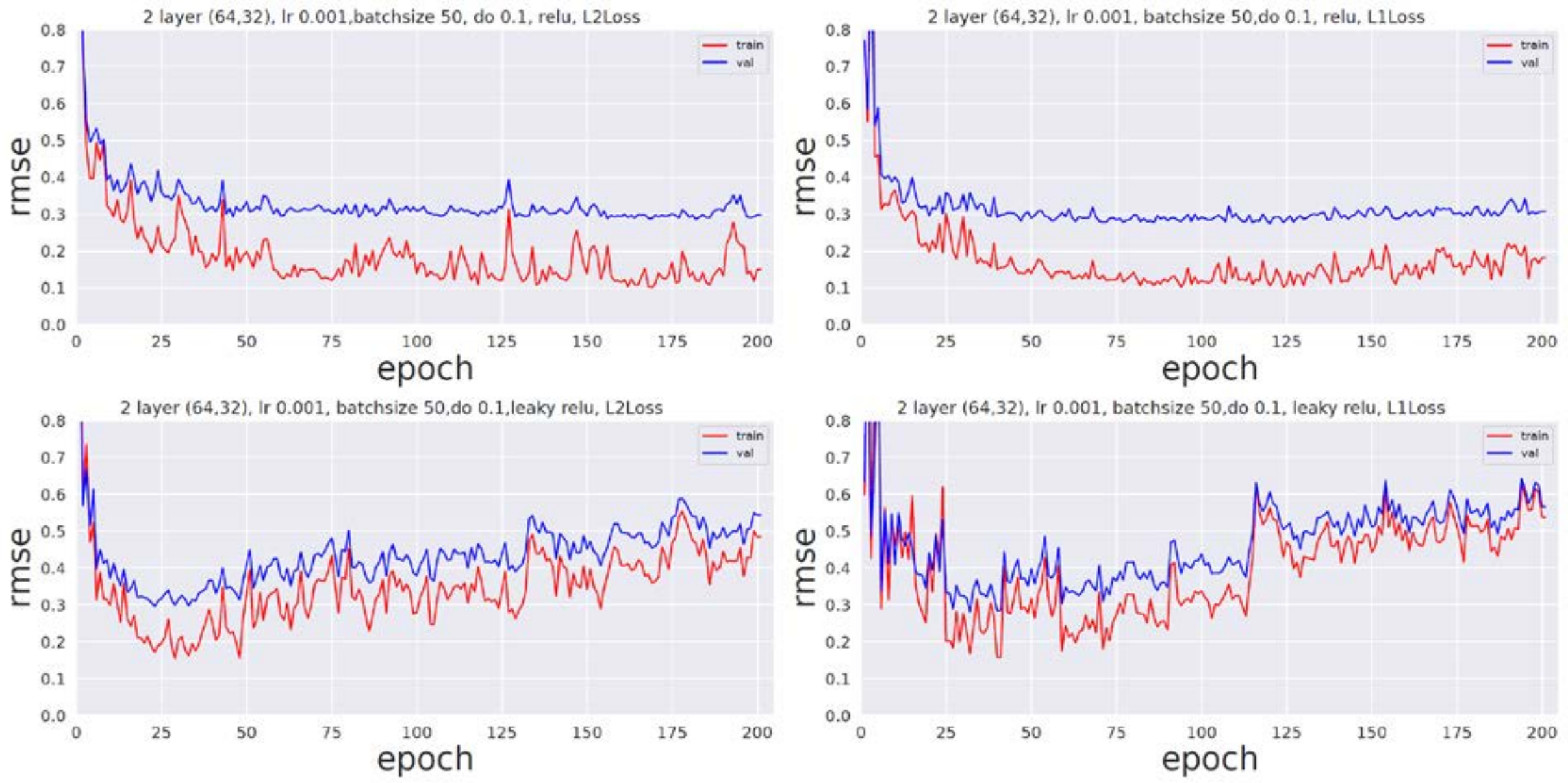

Figure SI1-36. Details on the hyperparameter optimization to find the optimal architecture and setup for the GNN to predict the *L* descriptor. For the GNN including 64 and 32 neurons per layer, respectively, and the identified optimal learning rate of 0.001, different activation and loss functions were tested.

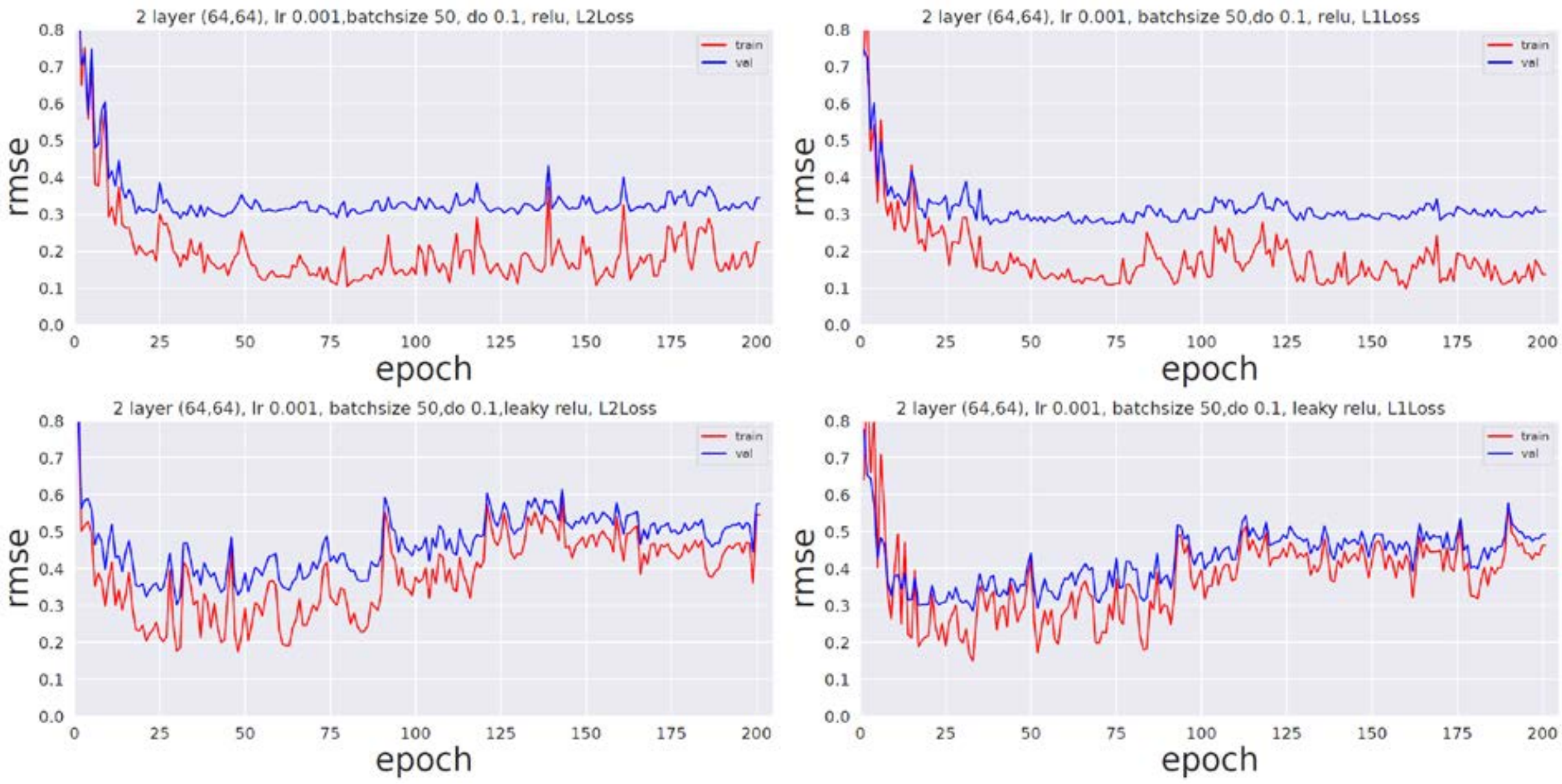

Figure SI1-37. Details on the hyperparameter optimization to find the optimal architecture and setup for the GNN to predict the *L* descriptor. For the GNN including 64 neurons per layer and the identified optimal learning rate of 0.001 different activation and loss functions were tested.

SI1-4. Detailed investigation of the GNN model predictions of the *A* descriptor.

As the experimental dataset for the *A* solute descriptor is imbalanced and includes many chemicals that cannot act as H-bond donors, we investigated the effect of the imbalance on our developed GNN model. We, therefore, checked whether the class $A = 0$ and the class $A \neq 0$ are predicted correctly, by setting the threshold to 0.01 (as we are applying a regression model here). As shown in the violin plot SI1-38, the model clearly identifies H-bond donors (false zeros for 3 chemicals, true zeros for 345 chemicals, whereas true-positive *A* values are predicted for 254 chemicals and false-positive zeroes are predicted for 33 chemicals). Note that there are only six chemicals for which an $A > 0.1$ was predicted. We investigated these cases in detail. For 1-[(4-phenylphenyl)methyl]pyridin-4-one, an *A* of 0.35 was predicted. This might not be entirely wrong, as a corresponding tautomeric form does include a hydroxy group. The same holds for 1,4-naphthoquinone, where an *A* of 0.40 was predicted. Other positive predicted cases are bornane-2,3-dione (A = 0.18) and 5'beta-rotenone (A = 0.13), where the corresponding tautomeric forms with hydroxy groups might explain the difference. Further, the *A* descriptor of 2,4,6-trimethylaniline is predicted to be 0.16, which is plausible for the structure. The last chemical with a predicted *A* of 0.11 is 2-hydroxybenzophenone, as there is a hydroxy group present in its structure. This might be plausible as well. For these examples, there might be an error in the experimental data. It should be noted that in the original Absolv solver files of M. Abraham, some of the *A* values are set to zero before the corresponding solute descriptors are determined, which might also introduce errors in the experimental dataset (especially for diones).

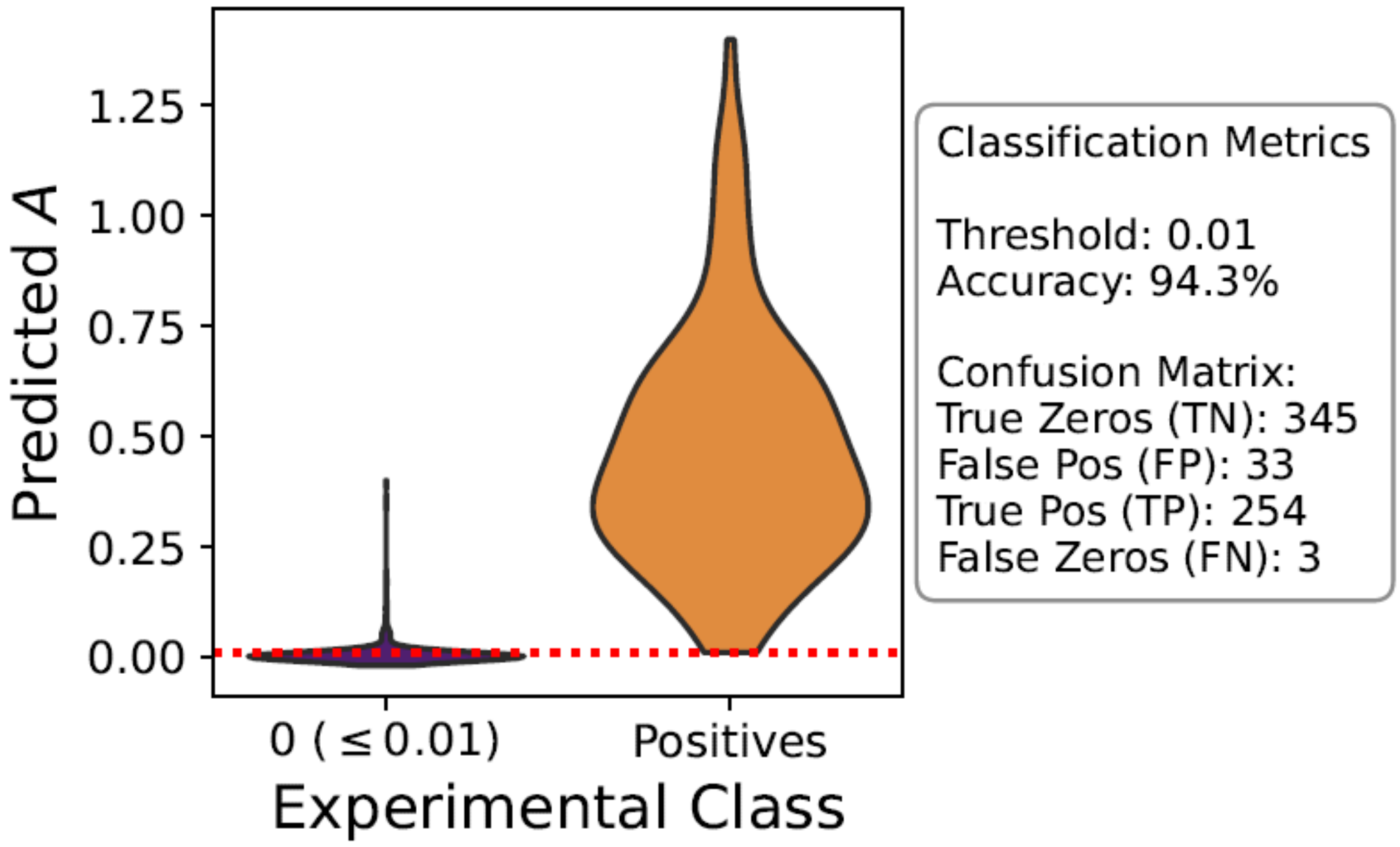

Figure **SI1-38**. Violin plot showing the test set predictions for the *A* descriptor. We investigated whether the imbalance of the training set impacted the corresponding performance of the GNN model.

SI1-5. Detailed analysis of test set predictions: correlation of solute descriptors.

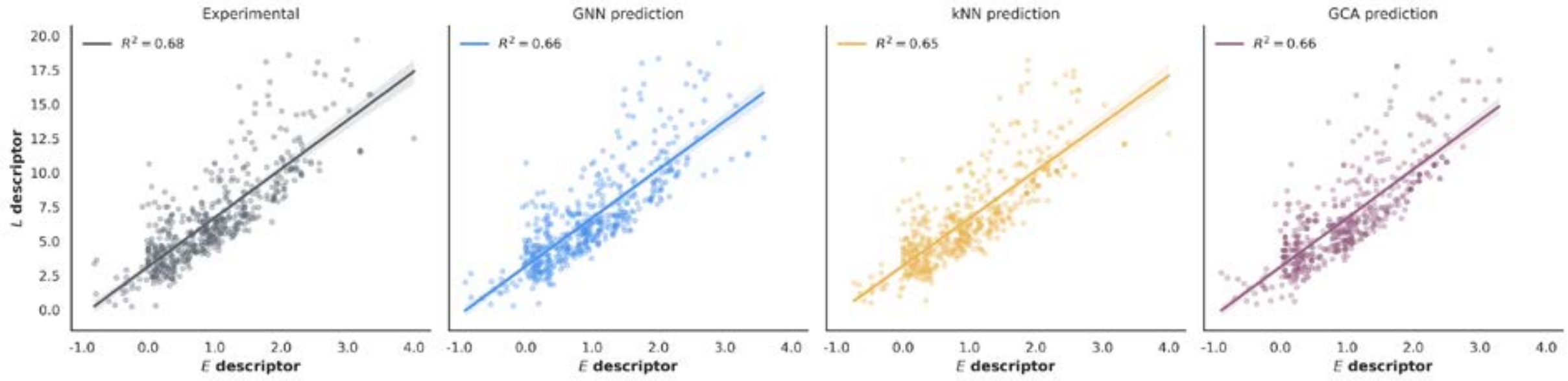

Figure SI1-39. Correlation of *L* and *E* descriptors of the test set chemicals are depicted for the experimental data and the three models. R^2 values of 0.65 to 0.68 indicate that the trend seen in the experimental data is captured correctly.

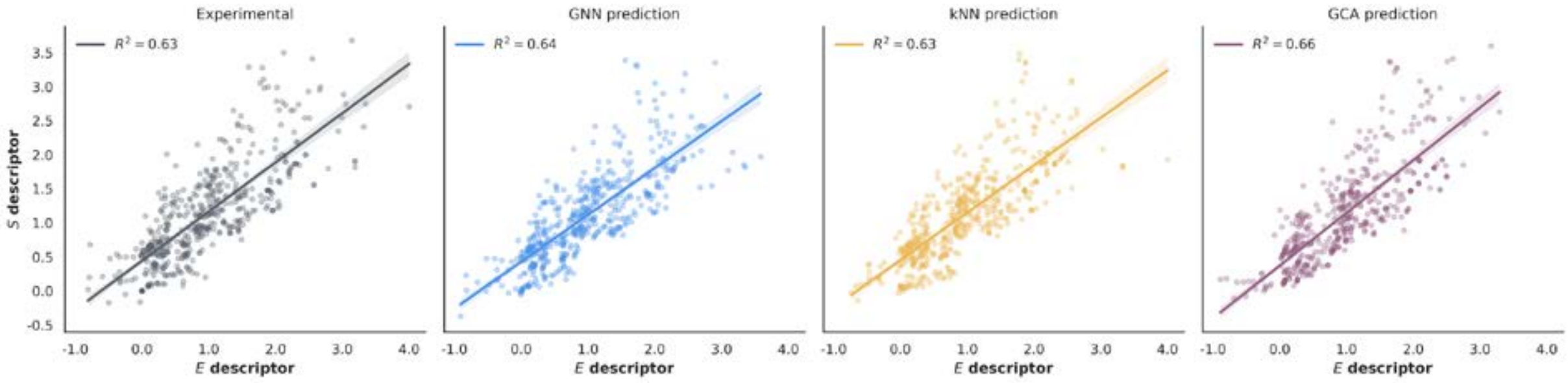

Figure SI1-40. Correlation of *S* and *E* descriptors for the test set chemicals are depicted for the experimental data and the three models. R^2 values of 0.63 to 0.66 indicate that the trend seen in the experimental data is captured correctly.

SI1-6. Detailed analysis of test set predictions: trends in *A* and *B* descriptors for specific functional groups.

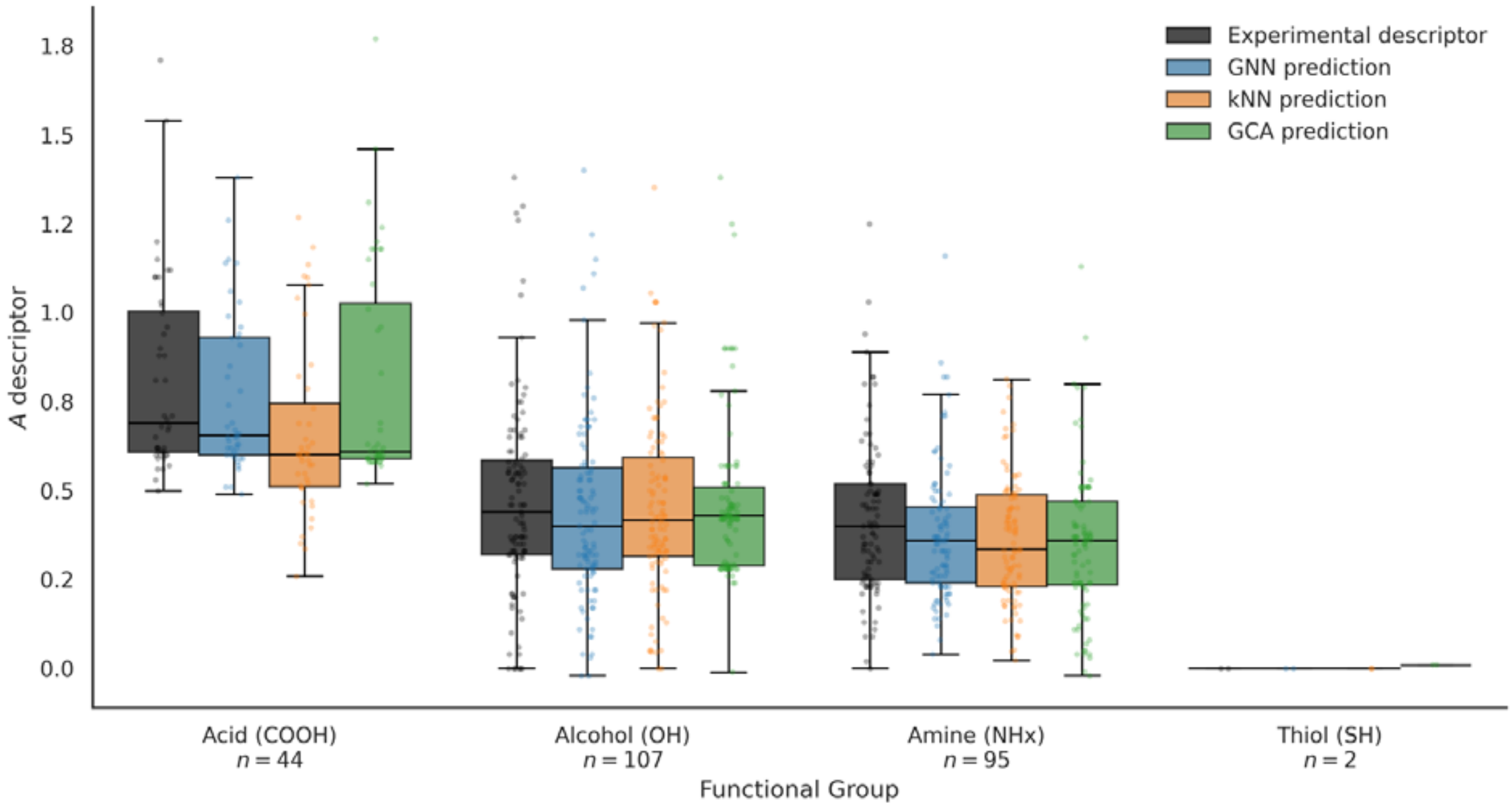

Figure SI1-41. Trends in the *A* descriptor for chemicals with different functional groups.

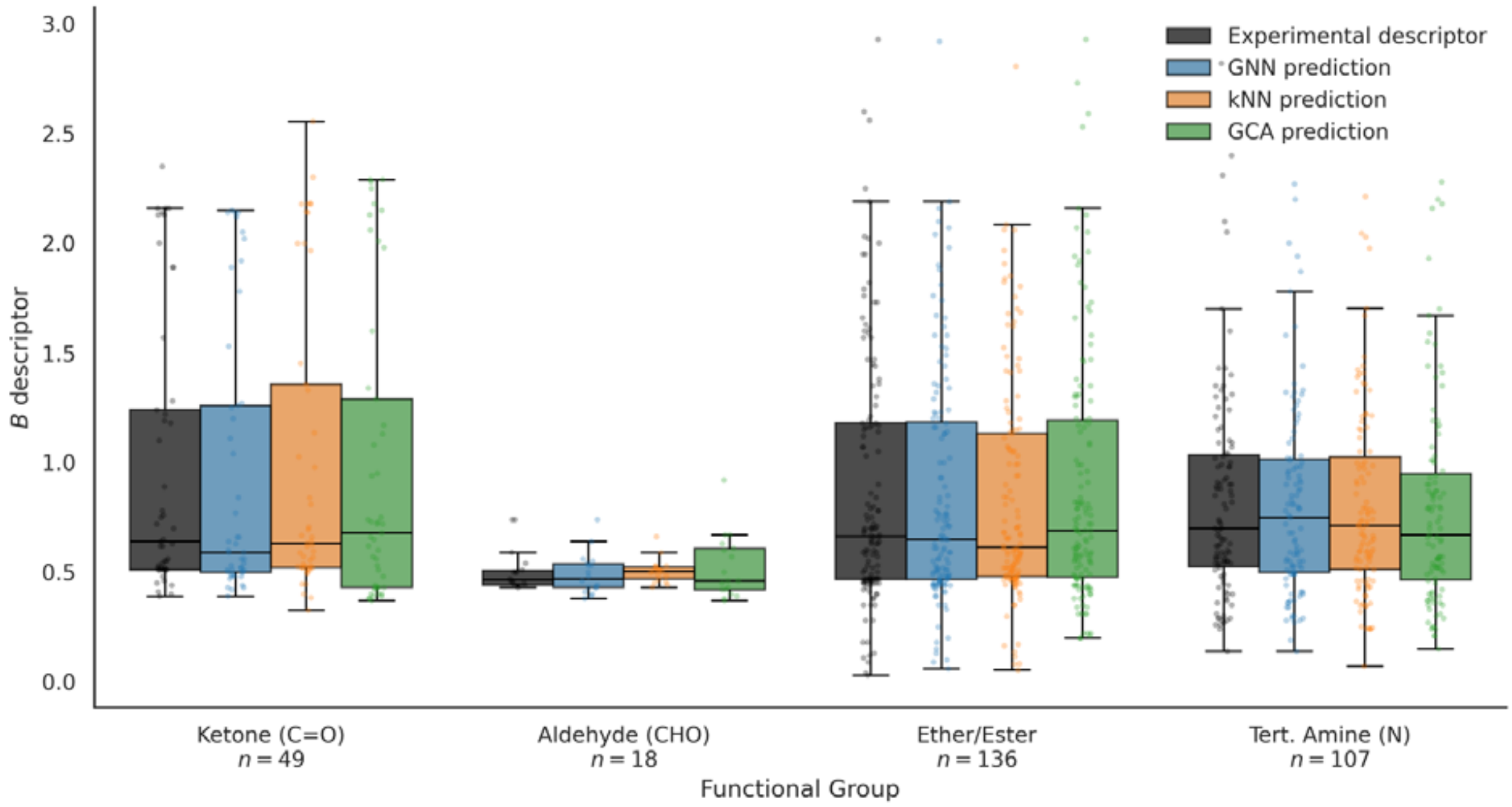

Figure SI1-42. Trends in the *B* descriptor for chemicals with different functional groups.

SI1-7. Detailed analysis of test set predictions: trends for homologous series for *L* and *E* descriptor.

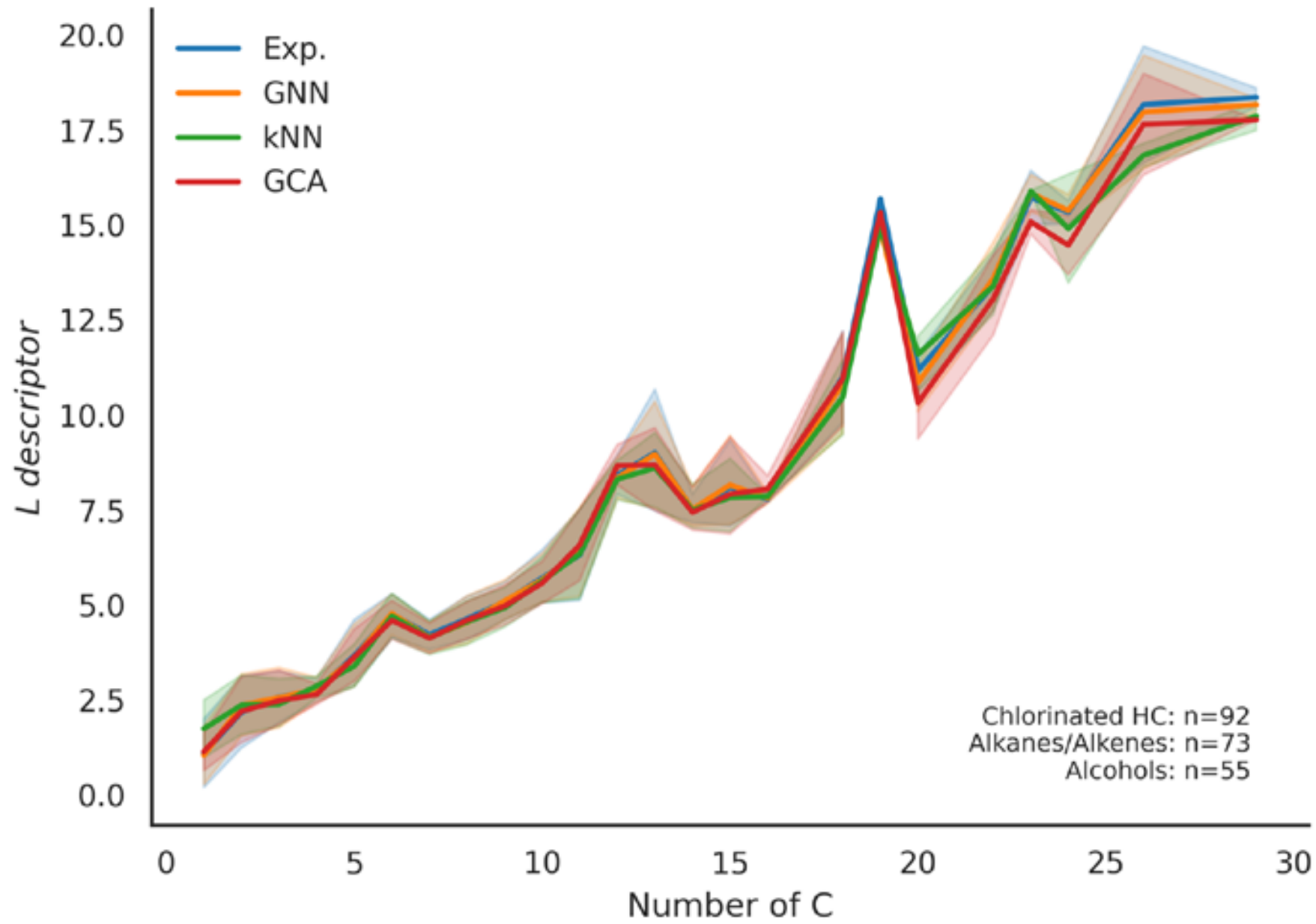

Figure SI1-43. Trend analysis in the *L* descriptor for homologous series of chlorinated hydrocarbons, alkanes, alkenes, and alcohols. The *L* descriptor increases with the number of carbons in the corresponding chemical structure for the experimental data and all models. Therefore, all models correctly depict the trend.

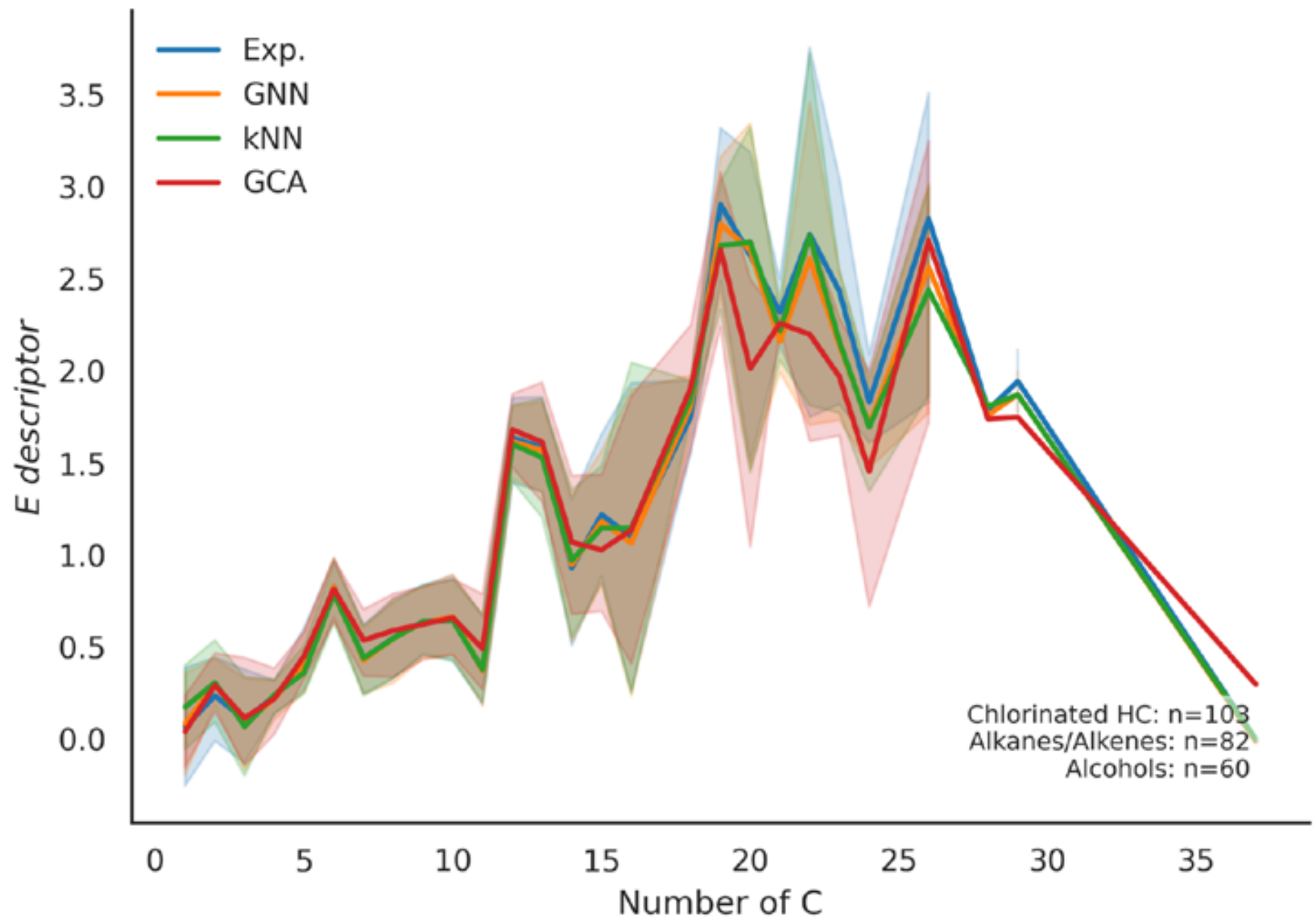

Figure SI1-44. Trend analysis in the *E* descriptor for homologous series of chlorinated hydrocarbons, alkanes, alkenes, and alcohols. The *E* descriptor increases with the number of carbons in the corresponding chemical structure for the experimental data and all models up to a carbon number of ~20, and then stagnates up to a carbon number of ~30. Again, the trend in the experimental data is correctly captured by the three models.

SI1-8. Consensus approach for the prediction of solute descriptors.

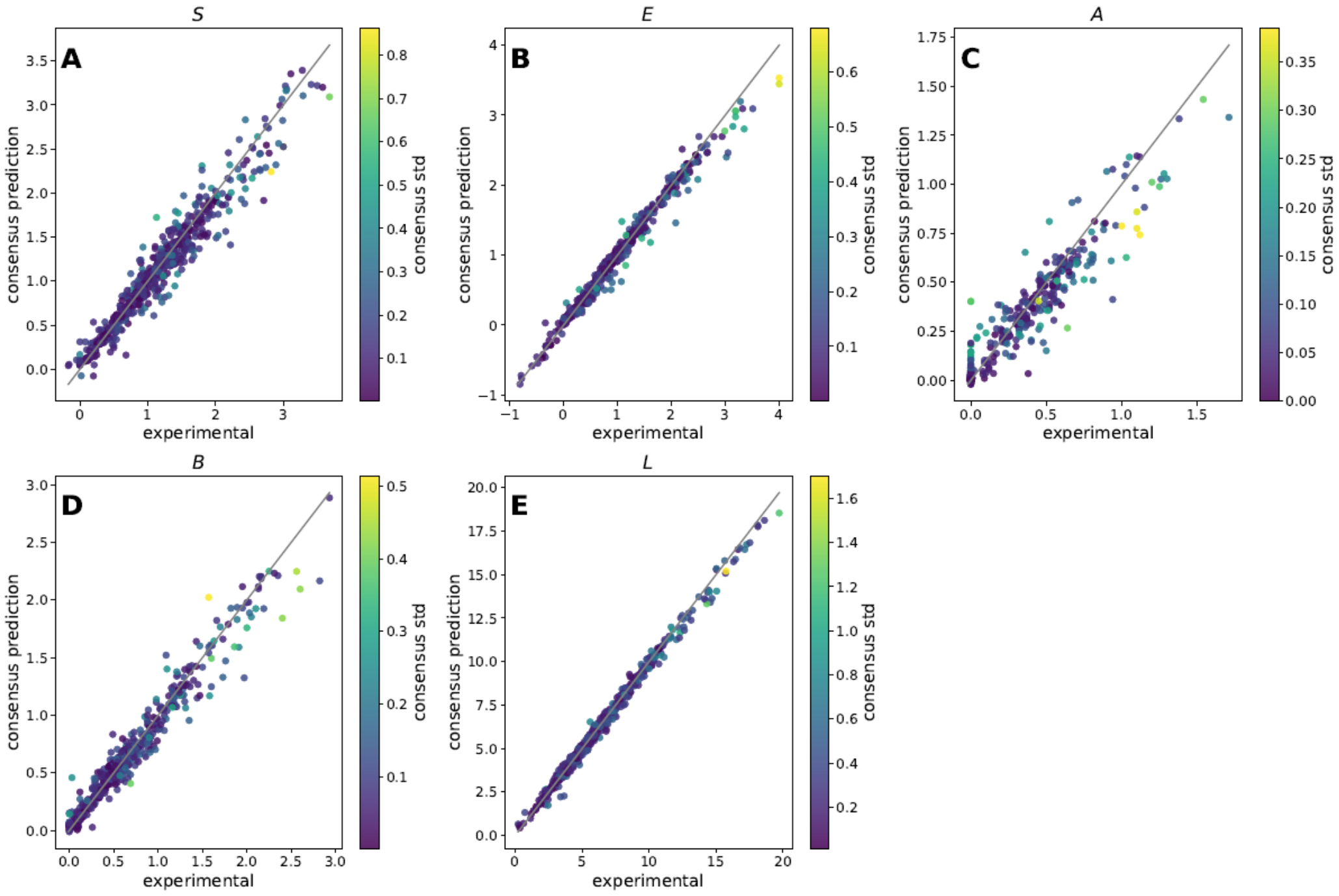

Figure SI1-45. Consensus predictions for the solute descriptors (A) *S*, (B) *E*, (C) *A*, (D) *B*, and (E) *L* of the independent test sets. The SD for each value is marked according to the color scale.

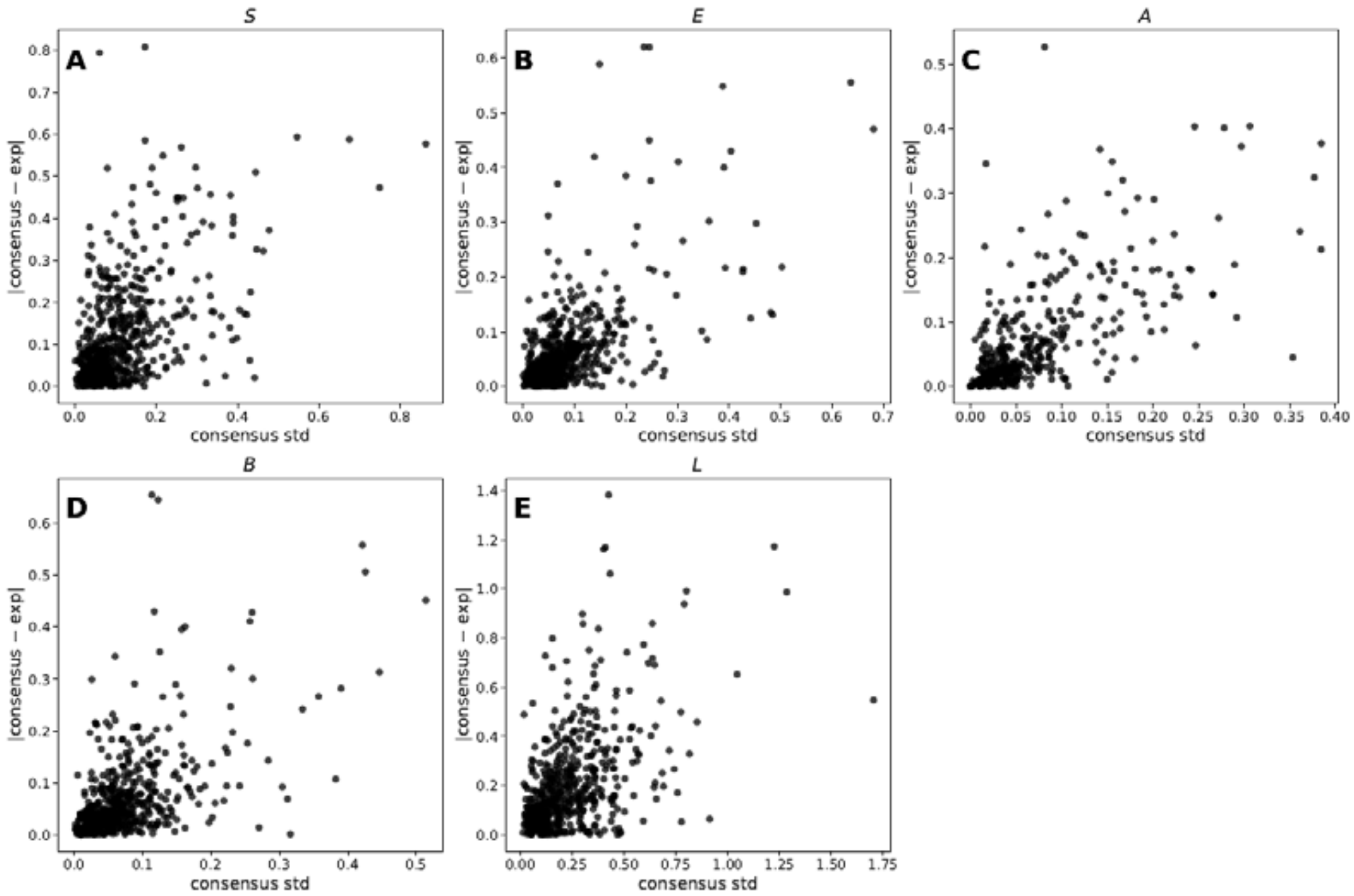

Figure SI1-46. Consensus predictions for the solute descriptors (A) *S*, (B) *E*, (C) *A*, (D) *B*, and (E) *L* of the independent test sets. The absolute difference between the predicted consensus value is plotted against the SD.

References

(1) Ulrich, N.; Ebert, A. Can deep learning algorithms enhance the prediction of solute descriptors for linear solvation energy relationship approaches? *Fluid Phase Equilibr* **2022**, *555*, 113349. DOI: https://doi.org/10.1016/j.fluid.2021.113349.
